# QSAR Model of Indeno[1,2-*b*]indole Derivatives and Identification of *N*-isopentyl-2-methyl-4,9-dioxo-4,9-Dihydronaphtho[2,3-*b*]furan-3-carboxamide as a Potent CK2 Inhibitor

**DOI:** 10.3390/molecules25010097

**Published:** 2019-12-26

**Authors:** Samer Haidar, Christelle Marminon, Dagmar Aichele, Abdelhamid Nacereddine, Wael Zeinyeh, Abdeslem Bouzina, Malika Berredjem, Laurent Ettouati, Zouhair Bouaziz, Marc Le Borgne, Joachim Jose

**Affiliations:** 1 Institut für Pharmazeutische und Medizinische Chemie, PharmaCampus, Westfälische Wilhelms-Universität Münster, Corrensstr. 48, 48149 Münster, Germany; shaid_01@uni-muenster.de (S.H.); daich_01@uni-muenster.de (D.A.); 2 Faculty of Pharmacy, 17 April street, Damascus University, Damascus P.O. Box 9411, Syria; 3 Faculté de Pharmacie—ISPB, EA 4446 Bioactive Molecules and Medicinal Chemistry, SFR Santé Lyon-Est CNRS UMS3453—INSERM US7, Université de Lyon, Université Claude Bernard Lyon 1, 8 Avenue Rockefeller, F-69373 Lyon CEDEX 8, France; christelle.marminon-davoust@univ-lyon1.fr (C.M.); a.nacereddine@gmail.com (A.N.); wael.zeinyeh@univ-lyon1.fr (W.Z.); bouzinaabdeslem@yahoo.fr (A.B.); laurent.ettouati@univ-lyon.fr (L.E.); zouhair.bouaziz@univ-lyon1.fr (Z.B.); marc.le-borgne@univ-lyon1.fr (M.L.B.); 4 Laboratory of Applied Organic Chemistry, Synthesis of Biomolecules and Molecular Modelling Group, Badji-Mokhtar—Annaba University, Box 12, Annaba 23000, Algeria; malika.berredjem@univ-annaba.org

**Keywords:** QSAR, cancer, CK2, indeno[1,2-*b*]indole, naphtho[2,3-*b*]furan-4,9-dione

## Abstract

Casein kinase II (CK2) is an intensively studied enzyme, involved in different diseases, cancer in particular. Different scaffolds were used to develop inhibitors of this enzyme. Here, we report on the synthesis and biological evaluation of twenty phenolic, ketonic, and *para*-quinonic indeno[1,2-*b*]indole derivatives as CK2 inhibitors. The most active compounds were 5-isopropyl-1-methyl-5,6,7,8-tetrahydroindeno[1,2-*b*]indole-9,10-dione **4h** and 1,3-dibromo-5-isopropyl-5,6,7,8-tetrahydroindeno[1,2-*b*]indole-9,10-dione **4w** with identical IC_50_ values of 0.11 µM. Furthermore, the development of a QSAR model based on the structure of indeno[1,2-*b*]indoles was performed. This model was used to predict the activity of 25 compounds with naphtho[2,3-*b*]furan-4,9-dione derivatives, which were previously predicted as CK2 inhibitors via a molecular modeling approach. The activities of four naphtho[2,3-*b*]furan-4,9-dione derivatives were determined in vitro and one of them (*N*-isopentyl-2-methyl-4,9-dioxo-4,9-dihydronaphtho[2,3-*b*]furan-3-carboxamide) turned out to inhibit CK2 with an IC_50_ value of 2.33 µM. All four candidates were able to reduce the cell viability by more than 60% after 24 h of incubation using 10 µM.

## 1. Introduction

Human CK2 (casein kinase II) is an S/T protein kinase, able to phosphorylate the bovine casein in vitro. By this means, it was one of the first phosphorylating enzymes discovered (in 1954) [1]. CK2 is a heterotetrameric enzyme, composed of two catalytic subunits (α and/or α′) and two regulatory subunits (β). It is involved in different intracellular signaling pathways and is also known to be an apoptosis-suppressor in cancer cells [2,3]. CK2 was shown to be overexpressed in cells of different cancer types, including prostate, breast, and colon tumors [4,5,6]. The overexpression of CK2 is not only related to cancer, but also to the development of other diseases, such as autoimmune diseases, cardiac hypertrophy, and central nervous system (CNS) diseases [7,8,9,10]. Up to now, the most common strategy to inhibit CK2 was to design small molecules that target the ATP-binding catalytic site [11]. A large number of compounds with different scaffolds have been described as active CK2 inhibitors [11,12,13,14], including compounds with an indeno[1,2-*b*]indole scaffold [15,16,17,18,19,20]. Among all known CK2 inhibitors, only one compound, 5-(3-chloro-phenylamino)-benzo[*c*][2,6]naphthyridine-8-carboxylic acid (CX-4945, Silmitasertib), has entered into clinical trial (phase II) as an orally available and selective inhibitor of protein kinase CK2 for the treatment of different kinds of cancer [21,22]. Different CK2 inhibitors were discovered via molecular modeling approaches (QSAR, docking, database mining, etc.), such as ellagic acid and quinalizarin [23]. The application of in silico techniques in discovering new CK2 inhibitors was reviewed recently in detail [23]. QSAR studies can be considered one of the main tools for drug discovery and design. This approach is based on the fact that biological activities of the tested compounds can be related to their structures. For this purpose, molecular descriptors representing the molecule’s characteristics are predicted and correlated with the activity in order to develop such QSAR models. Recently, we developed a structure-based pharmacophore model using indeno[1,2-*b*]indoles designed and tested as CK2 inhibitors [24]. The model was validated and subsequently used for the discovery of new inhibitors of CK2 using database mining. By screening the ZINC database [25], 55 candidates were initially selected; among them, the natural compound bikaverin turned out to be a potent inhibitor with an IC_50_ value of 1.24 µM [24,26]. Twenty-five compounds out of the 55 initially selected were naphtho[2,3-*b*]furan-4,9-dione derivatives, which had not been tested on kinase inhibition before [24]. In the present study, 20 indeno[1,2-*b*]indole derivatives were synthesized and their inhibitory activity was evaluated toward the target enzyme. Then, a QSAR model was developed and validated depending on the inhibitory activity of 30 indeno[1,2-*b*]indoles (10 were described earlier) on CK2 and used to predict the activity of 25 naphtho[2,3-*b*]furan-4,9-diones. The inhibitory activity of four naphtho[2,3-*b*]furan-4,9-diones namely 1-(3-(2-methyl-4,9-dioxo-4,9-dihydronaphtho[2,3-*b*]furan-3-carboxamido)propyl)-1*H*-imidazol-3-ium, 2-methyl-4,9-dioxo-*N*-(pyridin-3-ylmethyl)-4,9-dihydronaphtho[2,3-*b*]furan-3-carboxamide, *N*-isopentyl-2-methyl-4,9-dioxo-4,9-dihydronaphtho[2,3-*b*]furan-3-carboxamide, and 2-methyl-3(morpholine-4-carbonyl)naphto[2,3-*b*]furan-4,9-dione were tested using a CK2 holoenzyme. Additionally, the effect of the four naphtho[2,3-*b*]furan-4,9-dione compounds on breast cancer cells MCF-7 was investigated by determining the cell viability using an MTT assay.

## 2. Results and Discussion

A QSAR study aims to find a correlation between physicochemical properties of a list of compounds and their biological activities. A reliable QSAR model should be able to predict the activities of compounds not included in the training set [27]. Appropriate estimation of such a model is performed by using a statistical model and by determining an acceptable correlation coefficient (r^2^), as well as a correlation coefficient of the cross validation (q^2^) [28]. MOE software [29] can predict the pIC_50_s (pIC_50_ = −logIC_50_) and the predicted values will usually be closer to the actual values using less descriptors [30]. In this work, twenty active indeno[1,2-*b*]indoles with IC_50_ values between 25 and 4100 nM were used as a training set (Table 1) to develop a reliable QSAR model. This model was challenged against a test set of ten identically active indeno[1,2-*b*]indole derivatives, having IC_50_ values between 140 and 4160 nM (Table 2). The separation of compounds into training and test sets was done by selecting compounds with similar inhibitory activity and dividing those equally into both sets. In this way, compounds with different inhibitory activities ranging from highly active to moderately active were present equally in both sets. This strategy was applied because the compounds used in this study shared the same backbone and exhibited a high degree of chemical similarity. Consequently, compounds were divided into training and test sets randomly, while ensuring an identical range of biological activity. It is important to note that there are different methods for distributing the compounds into training and test sets, which have been intensively discussed in the literature [31,32]. Each separation method has its advantages and limitations. As a matter of fact, the random method used in this work is the most common method presented in the literature despite its disadvantages. Golbraikh and Tropsha discussed this point in detail [33,34]. They suggested that “the external test set must contain at least five compounds, representing the whole range of both descriptors and activities of compounds included into the training set,” which was accomplished in this work. The compounds used in the current study for both the training and test sets are structurally very similar, and the representative points in both sets were very close. As a result of the above-mentioned point, we believe that using the random method in the separation of the two sets was able to avoid bias in the results. The statistical parameters obtained were able to confirm the reliability of this model; however, this does not rule out that other separation methods might lead to a better robustness. For this work, twenty indeno[1,2-*b*]indole derivatives, namely **4d**, **4h**–**j**, **4p**–**s**, **4w**, **5a**, **5c**, **5f**, **5h**, **5g**, and **6a**–**f**, were synthesized as new derivatives. The synthesis procedure, as well as the analytical data, are given in the supporting information. In contrast, the following ten compounds used for the training and test sets, namely **4e**, **4f**, **4g**, **4v**, **4x**, **4y**, **5d**, **5j**, **5k**, and **6g**, which have been published before [16,17,35]. The inhibitory activity of all used indeno[1,2-*b*]indoles was tested on recombinant human CK2 and the inhibition results are shown in Table 1 and Table 2. The chemical structures of all compounds are given in SMILES formatin Table S1 in the supplementary information.

**Figure 1:**
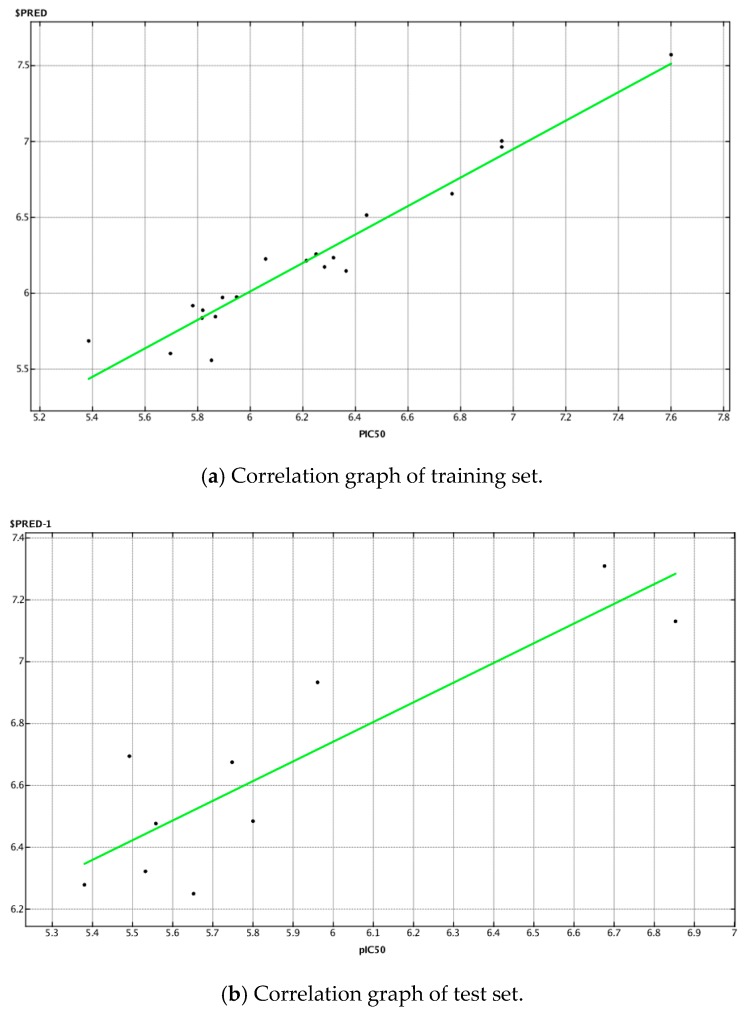
Correlation plot between the actual and the predicted pIC_50_ values of the compounds in the (**a**) training set and correlation plot between the actual and the predicted pIC_50_ values of the compounds in the test set (**b**).

In the present model, we calculated all 2D descriptors (206) that MOE offers, and selected the most important parameters using the statistical application functions “contingency” and “relative importance of descriptors” integrated in the software. Finally, 10 descriptors were selected to develop the current QSAR model using the PLS (partial least squares) method, which is a standard chemometric technique integrated into the software to determine the parameters of the linear model. The correlation coefficient r^2^ and the cross-validation coefficient q^2^ were 0.94 and 0.72, respectively. This model was able to predict the test set compounds with r^2^ equal to 0.77. Figure 1 presents the plot showing the difference between the actual and the predicted pIC_50_ values of the compounds in the training set and the test set. The equation for the current QSAR model with the ten necessary descriptors was as follows:

IC_50_ = 16.00545 + 0.06444 × PEOE_VSA+1 − 0.03995 × Q_VSA_HYD − 0.03312 × Q_VSA_PNEG − 0.06251 × SlogP_VSA7 − 0.03254 × SlogP_VSA8 + 0.03023 × SlogP_VSA9 + 0.03672 × SMR_VSA5 +0.00160 × weinerPath − 0.01269 × vdw_vol + 0.01078 × PEOE_VSA + 0.

The selected descriptors belong to the descriptors that depend on the partial charge of each atom of a chemical structure, subdivided into surface areas, adjacency, distance matrix descriptors, and physical properties. The subdivided surface areas of the descriptors are based on an approximate accessible van der Waals surface area (in Å^2^) calculated for each atom, where “SlogP” is the log of the octanol/water partition coefficient. This property is an atomic contribution model that calculates logP from the given structure, where the following descriptors SlogP_VSA7, SlogP_VSA8, SlogP_VSA9, and SMR_VSA5 belong to this group. The partial equalization of orbital electro negativities (PEOE) is a method for calculating atomic partial charges in which charge is transferred between bonded atoms until equilibrium. Descriptors prefixed with “Q_” use the partial charges stored with each structure in the database. Therefore, the descriptor “Q_VSA_HYD” is the total hydrophobic van der Waals surface area, “Q_VSA_PNEG” is the total negative polar van der Waals surface area, and “PEOE_VSA + 0 and PEOE_VSA + 1” use the sum of partial charges in different ranges. The Wiener path number is half the sum of all the distance matrix entries. Finally, the van der Waals volume (Å^3^) is an important physical property parameter [29]. These calculated parameters are given in Tables S2 and S3.

In the next step, we used our validated QSAR model to predict the pIC_50_ values of the 25 naphtho[2,3-*b*]furan-4,9-dione-based compounds that were selected earlier using in silico screening as possible hits for the target enzyme [24]. As it is clear from Table 3, many of the compounds were predicted to be highly active inhibitors. Most of them fit well in the active site of the enzyme, as confirmed by the docking study and the S scores obtained for all hits (Table 3). The above QSAR prediction and the docking results encouraged us to test the activity of these compounds in vitro. Unfortunately only four compounds were available to us and were tested for their in vitro CK2 activity, namely: **01893208**: 1-(3-(2-methyl-4,9-dioxo-4,9-dihydronaphtho[2,3-*b*]furan-3-carboxamido)propyl)-1*H*-imidazol-3-ium, **37867960**: 2-methyl-4,9-dioxo-*N*-(pyridin-3-ylmethyl)-4,9-dihydronaphtho[2,3-*b*]furan-3-carboxamide, **00082235**: *N*-isopentyl-2-methyl-4,9-dioxo-4,9-dihydronaphtho[2,3-*b*]furan-3-carboxamide, and **01236034**: 2-methyl-3(morpholine-4-carbonyl)naphto[2,3-*b*] furan-4,9-dione. The compounds were tested for inhibition with purified human protein kinase CK2 expressed in *Escherichia coli*, as described before [36], using a capillary electrophoresis-based activity assay. The in vitro results are depicted in Table 4. Among the tested compounds, only compound **00082235** showed a good CK2 inhibitory activity (75% at a concentration of 10 µM) with an IC_50_ value of 2.33 ± 0.25 µM, while the other three compounds were able to inhibit the target enzyme by around 35–45% at a concentration of 10 µM. The IC_50_ value was determined in three independent biological replicates with similar results (2.046, 2.527, and 2.423 µM). The mean value was determined. It is important to mention that compounds **01893208** and **00082235** were included recently in a patent claimed by Yang et al. and were evaluated for the treatment of proliferative and infectious diseases [37].

**Figure 2:**
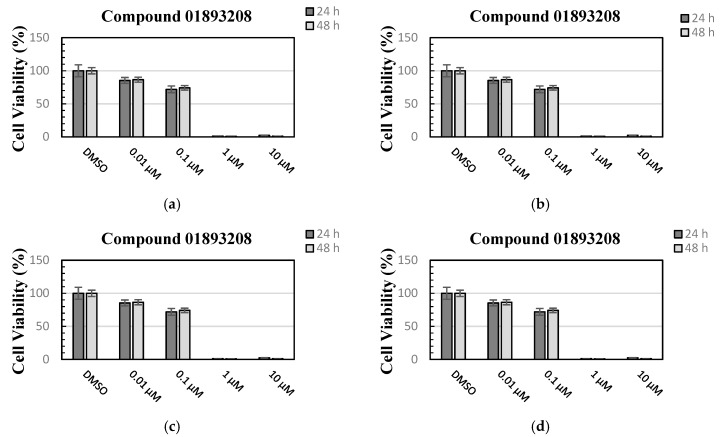
Cell viability was evaluated using an MTT assay with MCF-7 cells after 24 h and 48 h treatments with the compounds at 0.01, 0.1, 1 and 10 µM concentrations: (**a**) compound **01893208**, (**b**) compound **00082235**, (**c**) compound **37867960**, and (**d**) compound **01236034**. Bars represent the mean values of three measurements (*n* = 3). Error bars illustrate the corresponding standard deviations (± SD).

The effect of the four naphtho[2,3-*b*]furan-4,9-dione derivatives on cell viability was evaluated using an MTT assay with MCF-7 cells (human breast adenocarcinoma cell line). As it is shown in Figure 2, compounds **01893208**, **37867960**, and **00082235** were able to reduce the cell viability by more than 90% after 24 h and 48 h of incubation using 10 µM, while compounds **01893208** and **37867960** were able to reduce the cell viability by around 90% after 24 h and 48 h of incubation using 1 µM. In contrast, compound **01236034** was less cytotoxic and was able to reduce the cell viability to 60% after 48 h of incubation using 10 µM. These results indicate that the tested compounds showed a good effect on cell viability. It is important to note that some compounds with a naphtho[2,3-*b*]furan-4,9-dione backbone are known to have anticancer effects and are highly cytotoxic [38,39], and other natural naphtho[2,3-*b*]furan-4,9-dione derivatives, which are found in the lapacho (*Tabebuia*) tree, are known to have different biological activities, such as antibacterial, antifungal, anti-inflammatory, and antitumor activities [40].

**Figure 3:**
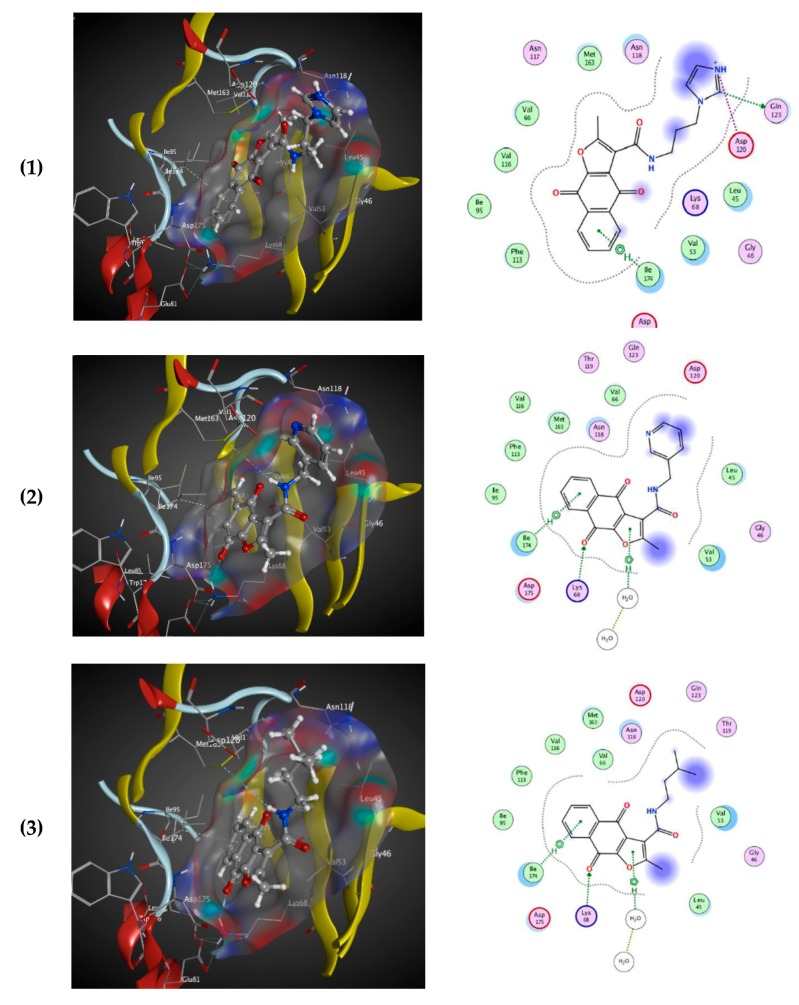

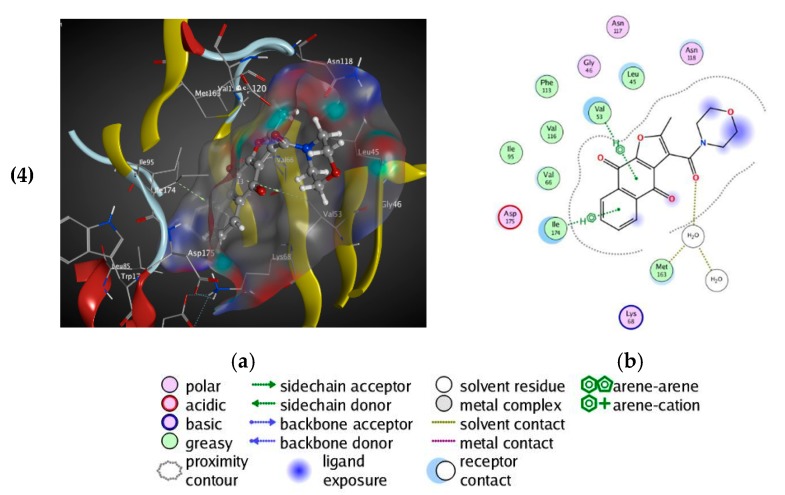
3D binding mode of the four tested compounds with the ATP binding site of CK2 with surface map (**a**,**b**) the 2D binding mode. (**1**) Compound **00082235**; (**2**) Compound **01893208**; (**3**) Compound **37867960**, (**4**) Compound **01236034**.

All naphtho[2,3-*b*]furan-4,9-dione compounds in this study were docked in the ATP binding site of the crystal structure of CK2, as mentioned above using PDB ID: 3C13 from the Protein Data Bank (PDB) [41], having a resolution of 1.95 Å. A conformational search for the 25 selected compounds was carried out using MOE, and all the resulting 219 conformations were used for the docking study. The conformations of all structures were docked in human CK2 enzyme using MOE and sorted according to their S score (energy-based scoring method implemented in MOE) to rank the best ligand in terms of the orientation and binding to the active site. The four tested compounds were selected and visual 2D and 3D inspections were carried out to exclude “false positives” due to assumptions and shortcomings in the docking methods and scoring function [42]. The active site was analyzed to determine the protein–ligand interaction to show exactly which atoms could interact. Figure 3 shows the 3D and 2D interactions of the four tested compounds and it is clear that they fit well in the ATP active site of CK2. The four docked compounds were inserted into the narrow ATP binding site of CK2 (Figure 3) and all of them were able to create a π–π interaction between the aromatic ring of the naphtho[2,3-b]furan-4,9-dione and the side-chain residues of Ile174. Compound **01893208** was also able to create direct bonding with Asp120 and Glu123. Compound **37867960** and compound **00082235** showed direct bonding with Lys68 and via a water molecule with Glu81. Compound **01236034** had direct bonding with Val53 and via a water molecule with Glu81 as well. Emodin in the crystal structure created a π–π interaction with Phe113 and direct bonding with Lys68.

Several studies have been performed to develop an active CK2 inhibitor using different in silico techniques [23]; however, few “ligand-based drug design” (LBDD) techniques were able to put forward a successful candidate [23]. Our database of indeno[1,2-*b*]indoles was not only used to find a list of candidates via a pharmacophore approach, but also exploited to develop a new reliable QSAR model, which we used to predict the activity of these compounds. We demonstrated that some of the tested naphtho[2,3-*b*]furan-4,9-dione compounds were active using both in vitro evaluation and cellular assays. Nevertheless, we were not able to correlate our predicted IC_50_ values to the tested IC_50_ values of naphtho[2,3-*b*]furan-4,9-dione compounds since only four compounds were available to us.

As a matter of fact, the moderate inhibitory activity of some tested naphtho[2,3-*b*]furan-4,9-dione derivatives was not a surprise for us, as a variation between predicted and tested inhibition is expected in such QSAR studies. This was also the case in some compounds in the test set, where the r^2^ for the test set was 0.77. Unfortunately, it was not possible to correlate the predicted and tested values for all naphtho[2,3-*b*]furan-4,9-dione derivatives presented in this study, since only four of them were tested. It is important to emphasize that the aim of this work was to describe the inhibitory activity of 20 novel indeno[1,2-*b*]indoles and to show that compounds with naphtho[2,3-*b*]furan-4,9-dione backbone were appropriate for developing CK2 inhibitors, which was proved by compound **00082235**. The future step will be optimizing this compound by synthesizing a series of compounds with different substituents on a naphtho[2,3-*b*]furan-4,9-dione backbone and performing a SAR study with the aim of finding a highly active CK2 inhibitor.

## 3. Materials and Methods

### 3.1. Chemistry

All indeno[1,2-*b*]indole derivatives **1**–**30** used in this study were synthesized by us. The procedures for the synthesis of indeno[1,2-*b*]indoles have already been described for compounds **4e** [35], **4f** [35], **4g** [17], **4v** [35], **4x** [16], **4y** [16], **5d** [17], **5j** [16], **5k** [16], and **6g** [16]. For the compounds **4d**, **4h**–**j**, **4p**–**s**, **4w**, **5a**, **5c**, **5f**, **5h**, **5g**, and **6a**–**f**, the chemistry is described in the Supporting Information (Files S1 and S2). Four naphtho[2,3-*b*]furan-4,9-dione derivatives were purchased from Life Chemicals (Woodbridge, CT, USA) and Enamine (Kyiv, Ukraine).

### 3.2. Computational Methods

#### 3.2.1. Computational Study

Molecular Operating Environment software package (MOE, 2016.01, Chemical Computing Group, Montreal, QC, Canada) was used to perform this study [29], running on an Intel Core, i5-6500CPU, 3.20 GHz processor.

#### 3.2.2. Data Set for QSAR

The data for the QSAR study (IC_50_ values) was created by using the above-mentioned compounds. The compounds were divided into a training set with 20 compounds having IC_50_ values between 25 and 4100 nM, and a test set with 10 compounds having IC_50_ values between 140 and 4160 nM. The compounds were divided into training and test sets randomly but maintained the same range of biological activity. All compounds were sketched using a building function integrated in MOE, converted to 3D, and their energy was minimized using an MMFF94 force field. Both the training and test sets were stored as a dataset in mdb files, and the pIC_50_ value for each compound was calculated and added manually.

#### 3.2.3. Molecular Descriptors

MOE offers a wide range of 2D and 3D molecular descriptors in order to calculate the molecular properties of compounds. In this work, all 2D descriptors (total 206) that MOE offers were calculated for all compounds in the training set. Then, the contingency (which is a statistical application) function integrated in the software was used to select the appropriate descriptors for this set of compounds. In the second step, we used the function “relative importance of descriptors,” and all descriptors with less than 0.1 were removed.

#### 3.2.4. QSAR Study

The QSAR model was developed by selecting the activity as “dependent variable” and the descriptors as model fields. After performing the regression analysis for the training set, the root mean square error (RMSE) and r^2^ values of the fit were calculated and the model was saved as a QSAR fit model, which was then used for the prediction of activities of the test data set. The QSAR fit was then used for the validation and cross-validation. Plotting the tested pIC_50_ values (X-axis) versus the predicted (PRED) values (Y-axis) was performed to assess the predictive ability of the model. The correlation coefficient (r^2^) was determined for the test set using the QSAR fit model and was used to predict the activity of the naphtho[2,3-*b*]furan-4,9-dione derivatives as well. The correlation coefficient r^2^ lies between 0 and 1, where 1 corresponds to an ideal fit.

#### 3.2.5. Crystal Structure from PDB

Three dimensional structure of the CK2 complex with emodin was obtained from the Protein Data Bank (PDB) using PDB ID: (3C13) with a resolution of 1.95 Å [41]. The structure was optimized by using the QuickPrep function implemented in the MOE software. Then, water molecules were removed from the structure and 3D protonation was done to change the state into an ionization level. In the second step, energy minimization was performed using default parameters, where the force field was Amber 10.

#### 3.2.6. Database Generation

The selected compounds from the ZINC database [23] (naphtho[2,3-*b*]furan-4,9-dione derivatives) were rebuilt with the MOE building option implemented in the software. The compounds were optimized by adding hydrogen atoms using the option found in the MOE software. The energy of the compounds was minimized using the following parameters gradient: 0.05, Force Field: MMFF94X, Chiral constraint, and Current Geometry. The conformation methodology was used to develop low energy conformations for each compound, applying the LowModMD method with an RMS gradient of 0.05, and all other parameters were used as their default. All the compounds and their conformations were saved in an mdb database and later employed for docking studies.

#### 3.2.7. Docking Study

The docking of the naphtho[2,3-*b*]furan-4,9-dione derivatives into the active site of the CK2 enzyme (3C13) was achieved using MOE-Dock implemented in MOE. The docking parameters were set as Rescoring 1: London dG, Placement: triangle matcher, Retain 30, Refinement Force field, and Rescoring 2: GBVI/WSA dG, Retain 30. The docking part of MOE can give the correct conformation of the ligand to obtain a minimum energy structure. The top conformation for each compound was selected based on the S score, and visual inspection in 2D and 3D was carried out using MOE. Prior to docking, the initial ligand from the complex structure was extracted. For the scoring function, lower scores indicated more favorable poses. The unit for the scoring function is kcal/mol, and the S score refers to the final score, which was the score of the last stage that was not set to None. The Lig X function in MOE was used for conducting interactive ligand modification and energy minimization in the active site of the receptor.

### 3.3. Biological Assays

#### 3.3.1. Capillary Electrophoresis-Based Assay for Testing the Inhibitors of the Human CK2

The available naphtho[2,3-*b*]furan-4,9-diones were tested for their inhibitory activity toward the human CK2 holoenzyme following the procedure described earlier [36]. Briefly, the synthetic peptide RRRDDDSDDD was used as the substrate, which is reported to be most efficiently phosphorylated by CK2. The purity of the CK2 holoenzyme was greater than 99%. For initial testing, inhibition was determined relative to the controls at inhibitor concentrations of 10 μM in DMSO as a solvent. The reaction with the pure solvent without an inhibitor was used as a positive control and set to 100% inhibition. Reactions without CK2 were used as a negative control and were taken as 0% inhibition. IC_50_ values were determined by measuring the CK2 inhibition at eight different concentrations ranging from 0.001 to 100 µM at appropriate intervals and calculated from the resulting dose-response curve [43].

#### 3.3.2. Cell Viability Assay

The effect of CK2 inhibitors on the viability of MCF-7 cells was evaluated using an MTT assay [44]. MCF-7 breast cancer cells (kindly provided by the Department of Clinical Radiology of the University Hospital Muenster, Germany) were cultured in RPMI 1640 medium containing GlutaMax (Life Technologies, Darmstadt, Germany) and 10% fetal calf serum. The MTT assay was performed in 96-well plates. Cells were seeded at a density of 1 × 10^5^ cells per well, then incubated for 24 or 48 h at 37 °C in a humidified atmosphere (5% CO_2_). After overnight incubation, the seeding medium was removed and replaced with fresh medium containing the inhibitor at 0.01, 0.1, 1, and 10 μM. DMSO at a final concentration of 1% served as a control. Afterward, the MTT reagent (Sigma Aldrich, Darmstadt, Germany) was added at a final concentration of 0.5 mg/mL. After incubation for 2 h at 37 °C, the medium was discarded and 200 µL DMSO was added for the solubilization of the formazan. After mixing, the absorption was determined at 570 nm with a reference wavelength of 630 nm using a microplate reader. CK2 inhibitors were tested in triplicate, and the experiments were repeated three times.

## 4. Conclusions

In this work, we described a set of indeno[1,2-*b*]indoles as CK2 inhibitors, together with their in vitro activities, and further presented a detailed description of a QSAR study built from these indeno[1,2-*b*]indoles. The obtained QSAR model was exploited to predict the activity of a group of naphtho[2,3-*b*]furan-4,9-diones that have never been tested as CK2 inhibitors before. Through our current results, we can confirm that a naphtho[2,3-*b*]furan-4,9-dione backbone is an appropriate skeleton for the development of novel inhibitors for this kinase target. More compounds having this backbone with different functional groups should be designed with the aim of finding highly active CK2 inhibitors.

## Figures and Tables

**Table 1:** Chemical structures of the indeno[1,2-*b*]indoles with their tested and predicted IC_50_ and pIC_50_ values used for the training set.

Nr.	Chemical Structure	Tested (IC_50_ nM) pIC_50_	Pred. (IC_50_ nM) pIC_50_	Nr.	Chemical Structure	Tested (IC_50_ nM) pIC_50_	Pred. (IC_50_ nM) pIC_50_
**4e**	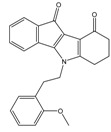	(1400) 5.8540	(2745) 5.5610	**4y**	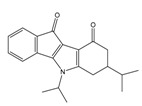	(610) 6.2150	(600) 6.2178
**4f**	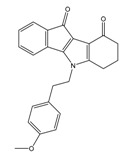	(4100) 5.3870	(2050) 5.6888	**5d**	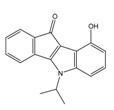	(2000) 5.6980	(2490) 5.6058
**4g**	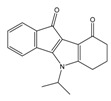	(360) 6.4440	(304) 6.5177	**5g**	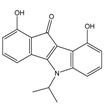	(560) 6.2520	(549) 6.2605
**4h**	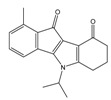	(110) 6.9580	(98) 7.0060	**5h**	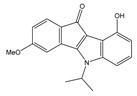	(480) 6.3180	(580) 6.2371
**4p**	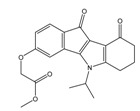	(1520) 5.8180	(1490) 5.8394	**5j**	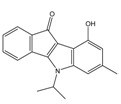	(1270) 5.8960	(1060) 5.9747
**4r**	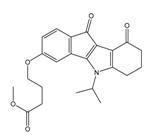	(520) 6.2840	(666) 6.1762	**5k**	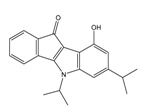	(1650) 5.8380	(1230) 5.9210
**4s**	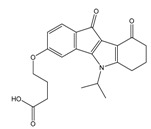	(1510) 5.8210	(1285) 5.8909	**6b**	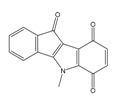	(1120) 5.950	(1050) 5.9779
**4v**	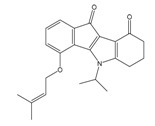	(25) 7.6020	(27) 7.5749	**6d**	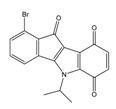	(1350) 5.8690	(1417) 5.8485
**4w**	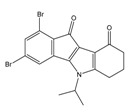	(110) 6.9580	(108) 6.9667	**6f**	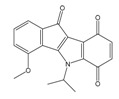	(870) 6.0600	(590) 6.2289
**4x**	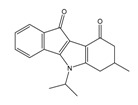	(170) 6.7690	(220) 6.6584	**6g**	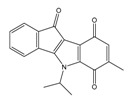	(430) 6.3660	(708) 6.1497

**Table 2:** Chemical structures of the indeno[1,2-*b*]indoles with their tested and predicted IC_50_ and pIC_50_ values used for the test set.

Nr.	Chemical Structure	Tested (IC_50_ nM) pIC_50_	Pred. (IC_50_ nM) pIC_50_	Nr.	Chemical Structure	Tested (IC_50_ nM) pIC_50_	Pred. (IC_50_ nM) pIC_50_
**4d**	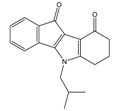	(1090) 5.9620	(116) 6.9346	**5c**	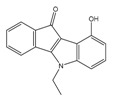	(2760) 5.559	(333) 6.4781
**4i**	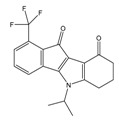	(210) 6.6770	(48) 7.3107	**5f**	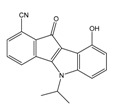	(2930) 5.5330	(475) 6.3235
**4j**	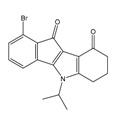	(140) 6.8540	(74) 7.1318	**6a**	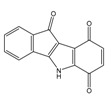	(1780) 5.749	(210) 6.6764
**4q**	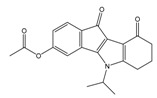	(3210) 5.4930	(201) 6.6959	**6c**	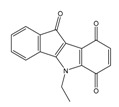	(1580) 5.801	(325) 6.4858
**5a**	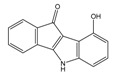	(2220) 5.6530	(560) 6.2513	**6e**	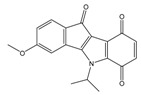	(4160) 5.3810	(524) 6.2800

**Table 3:**
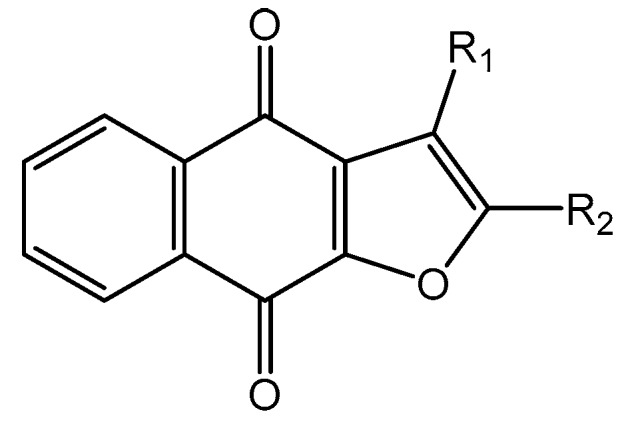
Chemical structures of the (hit) compounds from 3D mining of the ZINC database using the pharmacophore features of the indeno[1,2-*b*]indole as CK2 inhibitors, together with the predicted IC_50_s, pIC_50_s, and S score.

ZINC Code	Chemical Structure	Pred. (IC_50_ nM)/pIC_50_	S Score	ZINC Code	Chemical Structure	Pred. (IC_50_ nM)/pIC_50_	S Score
**01893208**	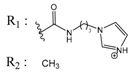	(849) 6.0711	−7.0598	**002700665**	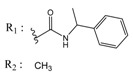	(118.10^4^) 2.9347	−6.9187
**37867960**	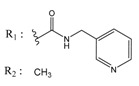	(4650) 5.3323	−7.0005	**00449156**	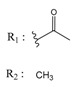	(71) 7.1470	−5.8886
**02700659**	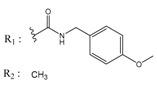	(292) 6.5347	−7.0460	**0079314**	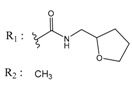	(61) 7.2195	−6.8687
**01214105**	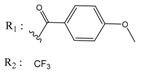	(2930) 5.5334	−6.5439	**04776566**	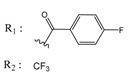	(46600) 4.3312	−6.2294
**00082235**	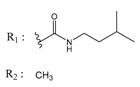	(1145) 5.9413	−6.7446	**00720964**	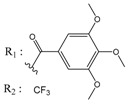	(19) 7.7102	−6.6344
**00978111**	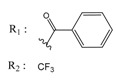	(245500) 3.6018	−6.0406	**10034949**	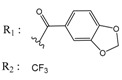	(21) 7.6758	−6.7250
**53166374**	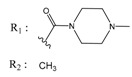	(82) 7.0870	−6.4533	**10034950**	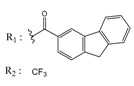	(35.10^5^) 2.4537	−6.2979
**02700667**	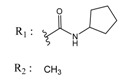	(8550) 5.0684	−6.6746	**00065262**	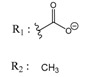	(11) 7.9146	−5.9976
**01236034**	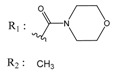	(0.08) 10.0672	−6.4042	**01214623**	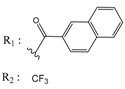	(16.10^6^) 1.7974	−6.4351
**01236036**	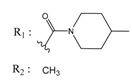	(3900) 5.4007	−6.3467	**05776381**	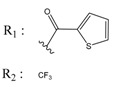	(8550) 5.0608	−6.2530
**01236042**	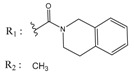	(5700) 5.2432	−6.8031	**10034955**	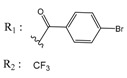	(108000) 3.9677	−6.1499
**01236043**	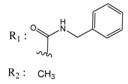	(28500) 4.5408	−6.8501	**00721112**	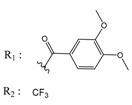	(450) 6.333	−6.6130
**01236046**	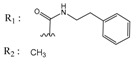	(9.10^5^) 3.0440	−7.0466				

**Table 4:** Chemical structures of the tested compounds together with the in vitro inhibitory activity toward CK2.

ZINC Code	Chemical Structure	% Inhibitory ^a^ (IC_50_^b^) nM	Pred. (IC_50_ nM)/pIC_50_
**01893208**	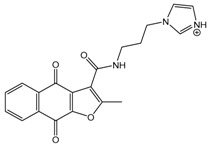	39 (12000) ^c^	(845) 6.0711
**37867960**	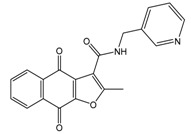	35 (14000) ^c^	(4650) 5.3323
**00082235**	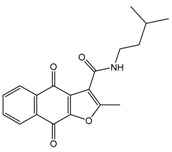	75 (2330)	(1145) 5.9413
**01236034**	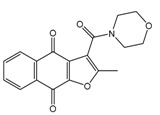	45 (11000) ^c^	(0.08) 10.0672

^a^ The percentage of inhibition of CK2 activity was determined for each compound at a fixed concentration of 10 µM. ^b^ The IC_50_ value was determined only for compounds with an initial inhibitory activity above 50% at a concentration of 10 µM. Here, the concentration was varied to precisely determine the IC_50_ value. ^c^ A rough estimation was obtained for less potent compounds from the experimental inhibition produced at 10 µM.

## Supplementary Materials

File S1. Chemical synthesis of all new indeno[1,2-*b*]indoles. File S2. The NMR and MS data for all new indeno[1,2-*b*]indoles. Table S1: Chemical structures of the indeno[1,2-b]indoles used for the training set and the test set in the SMILES format. Tables S2 and S3: calculated physicochemical parameters of the training set and the test set.
