# Supplementary material for: QSAR Model of Indeno[1,2-b]indole Derivatives and Identification of N-isopentyl-2-methyl-4,9-dioxo-4,9-Dihydronaphtho[2,3-b]furan-3-carboxamide as a Potent CK2 Inhibitor
Source: Molecules. 2019 Dec 26;25(1):97. doi: 10.3390/molecules25010097 (PMC6982966; doi:10.3390/molecules25010097)

QSAR model of indeno[1,2-*b*]indole derivatives and identification of *N*-isopentyl-2-methyl-4,9-dioxo-4,9-dihydronaphtho[2,3-*b*]furan-3-carboxamide as potent CK2 inhibitor

Samer Haidar[a,b,] , Christelle Marminon[c], Dagmar Aichele[a], Abdelhamid Nacereddine[c], Wael Zeinyeh[c], Abdeslem Bouzina[c,d], Malika Berredjem[d], Laurent Ettouati[c], Zouhair Bouaziz[c], Marc Le Borgne[c], Joachim Jose[a]*

NMR and HR-MS spectra

5-Isobutyl-5,6,7,8-tetrahydroindeno[1,2-*b*]indole-9,10-dione (4d)

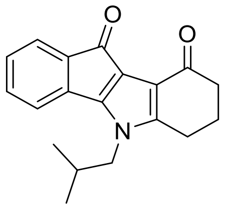

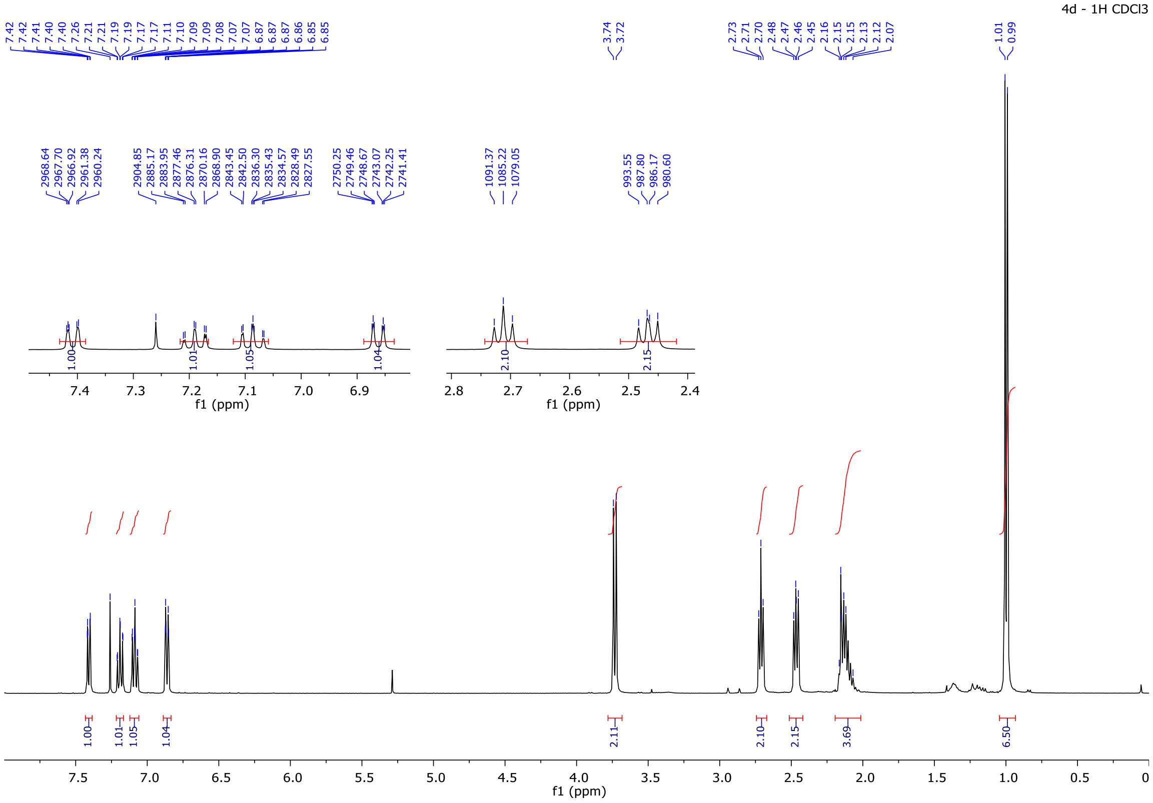

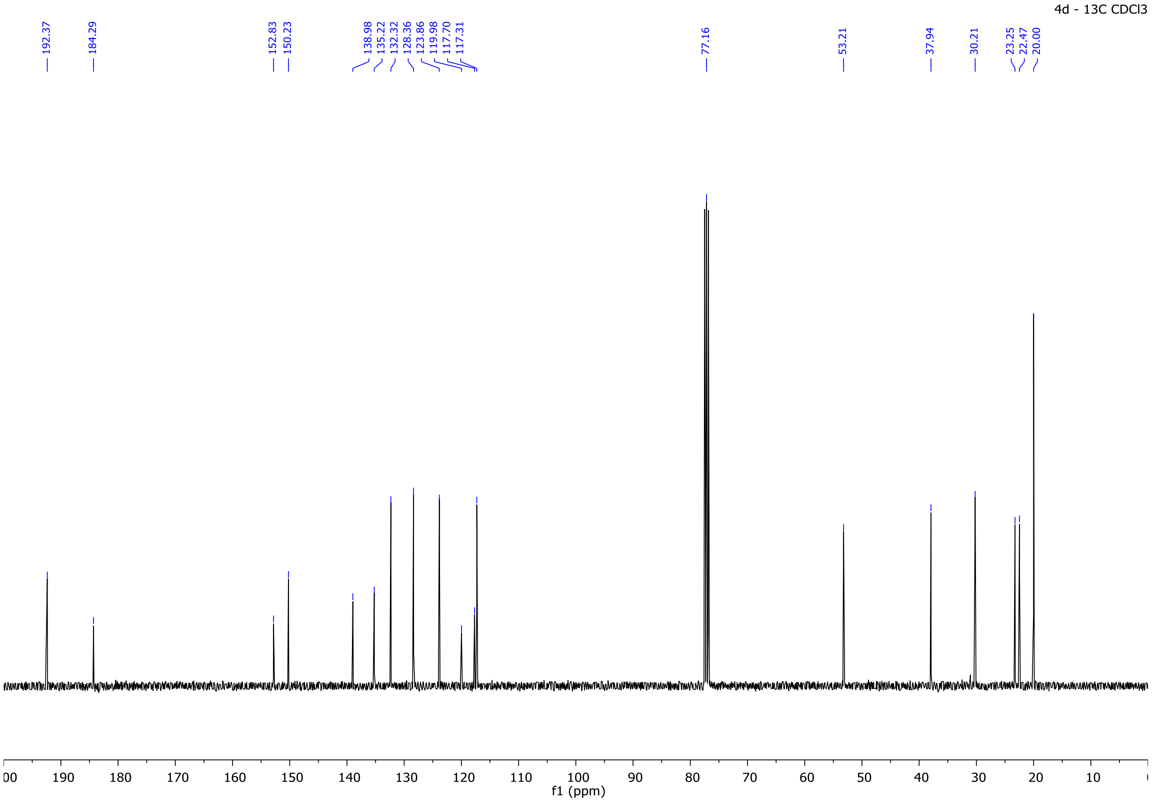

Analysis Info

Analysis Name	QTOF_120712_07_AP03.d		
Method	MS_inf_TL_50_1000_Pos_CCSM_2.m	Acquisition Date	7/12/2012 4:11:36 PM
Comment		Instrument / Ser#	micrOTOF-Q II 10231

Acquisition Parameter

Source Type	ESI	Ion Polarity	Positive	Set Nebulizer	0.4 Bar
Focus	Active	Set Capillary	1500 V	Set Dry Heater	200 °C
Scan Begin	50 m/z	Set End Plate Offset	-500 V	Set Dry Gas	4.0 l/min
Scan End	1000 m/z	Set Collision Cell RF	50.0 Vpp	Set Divert Valve	Waste

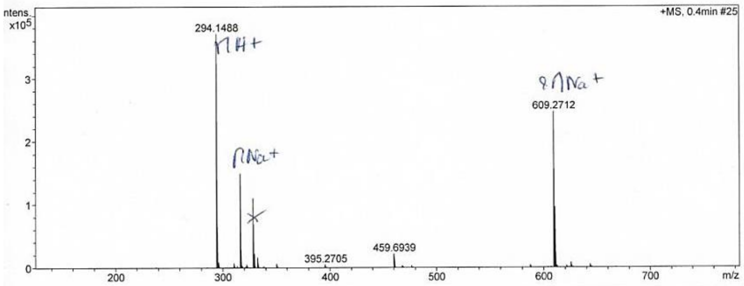

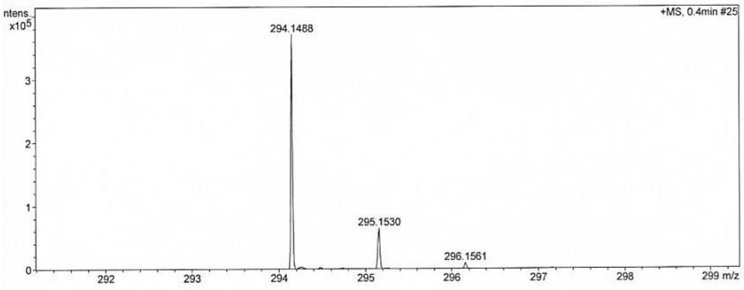

Meas. m/z	Formula	m/z	err [ppm]	mSigma
294.1488	C 19 H 20 N O 2	294.1489	0.1	20.4

5-Isopropyl-1-methyl-5,6,7,8-tetrahydroindeno[1,2-*b*]indole-9,10-dione (4h)

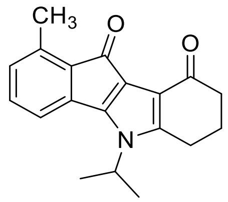

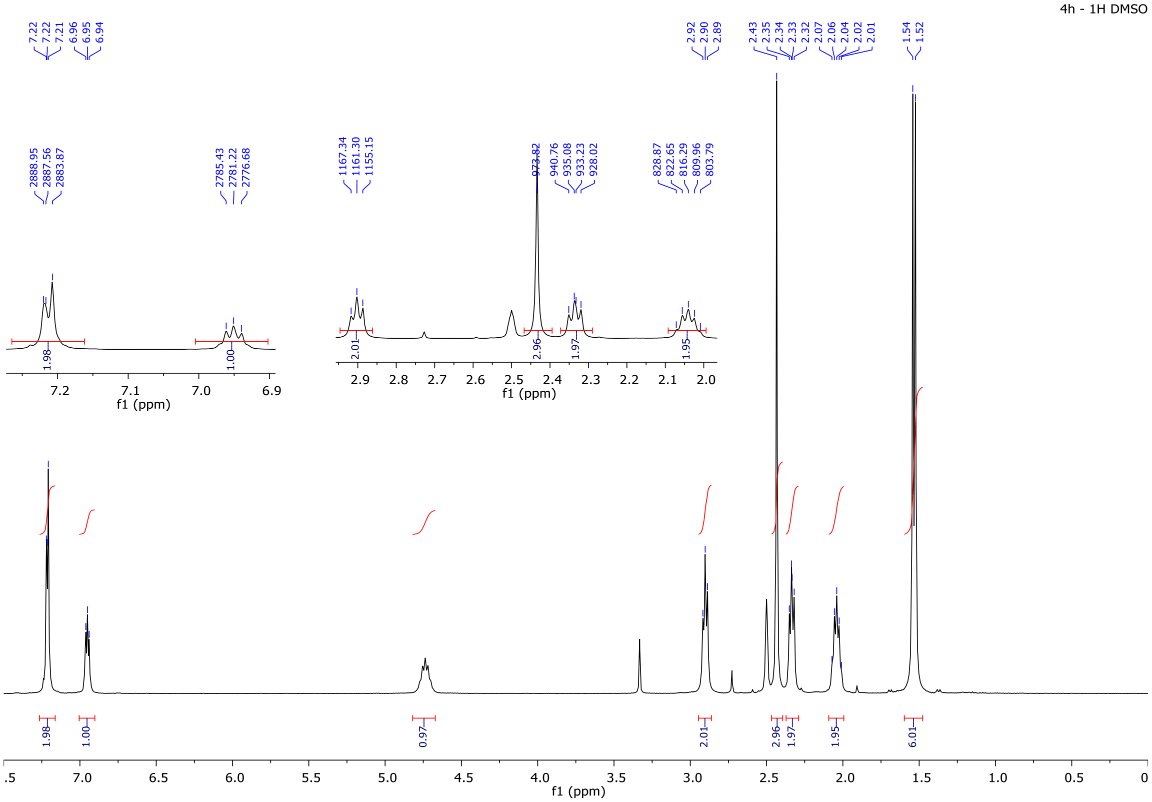

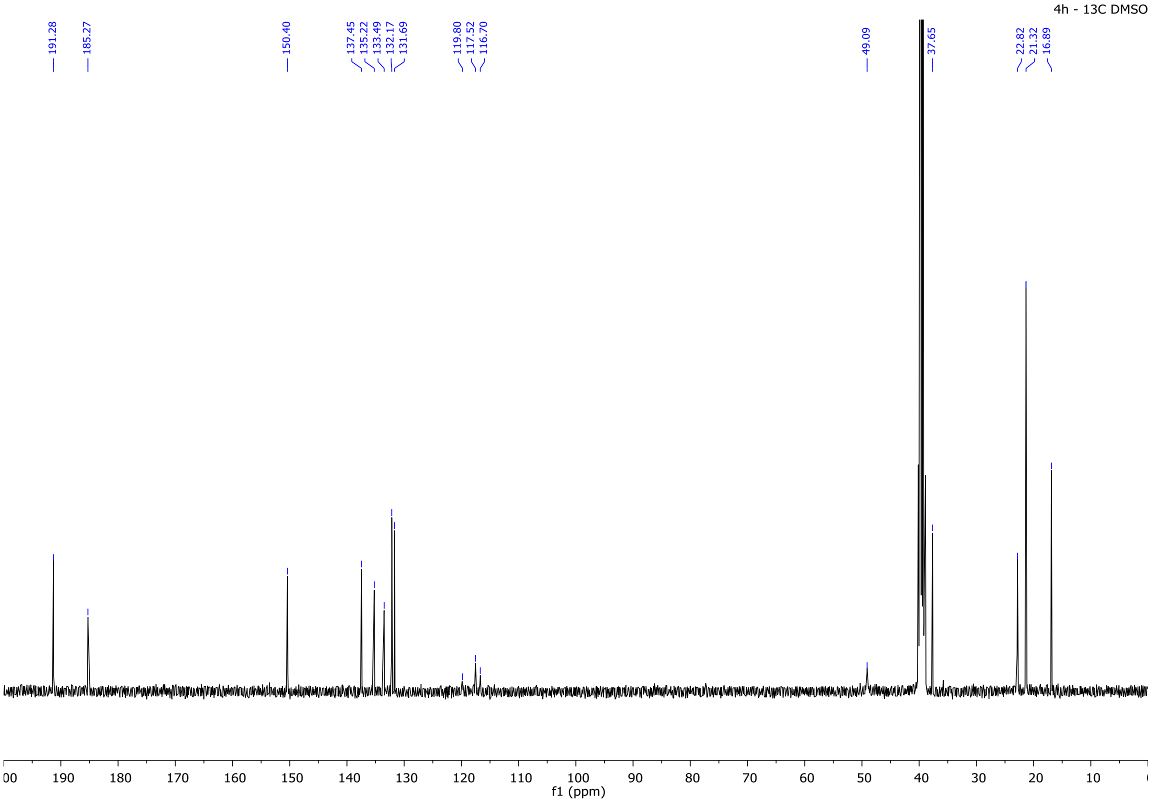

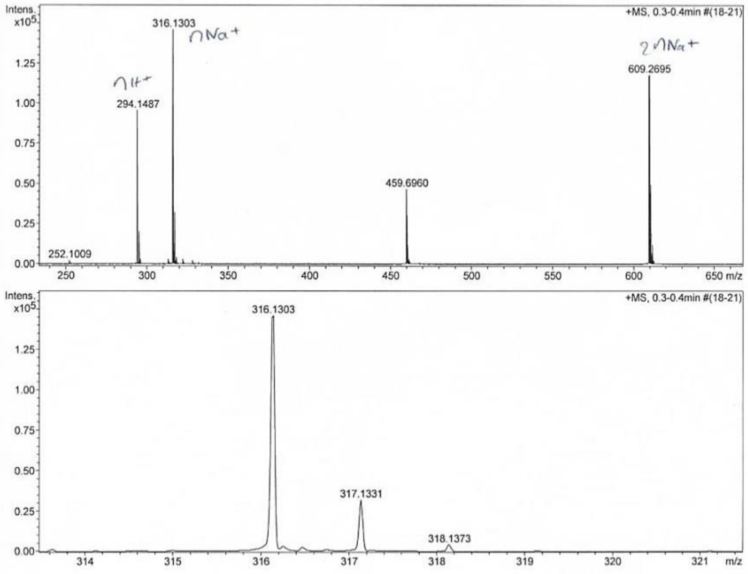
Analysis Info

Analysis Name	QTOF_140418_14_CM3146B.d		
Method	MS_inf_TL_50_1000_Pos_CCSM_2.m	Acquisition Date	4/18/2014 2:22:43 PM
Comment		Instrument / Ser#	micrOTOF-Q II 10231

Acquisition Parameter

Source Type	ESI	Ion Polarity	Positive	Set Nebulizer	0.6 Bar
Focus	Not active	Set Capillary	1500 V	Set Dry Heater	200 °C
Scan Begin	50 m/z	Set End Plate Offset	-500 V	Set Dry Gas	4.0 l/min
Scan End	1000 m/z	Set Collision Cell RF	10.0 Vpp	Set Divert Valve	Waste

Meas. m/z	Formula	m/z	err [ppm]	mSigma
316.1303	C 19 H 19 N Na O 2	316.1308	1.5	6.7

5-Isopropyl-1-(trifluoromethyl)-5,6,7,8-tetrahydroindeno[1,2-*b*]indole-9,10-dione (4i)

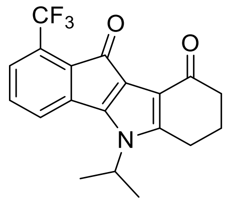

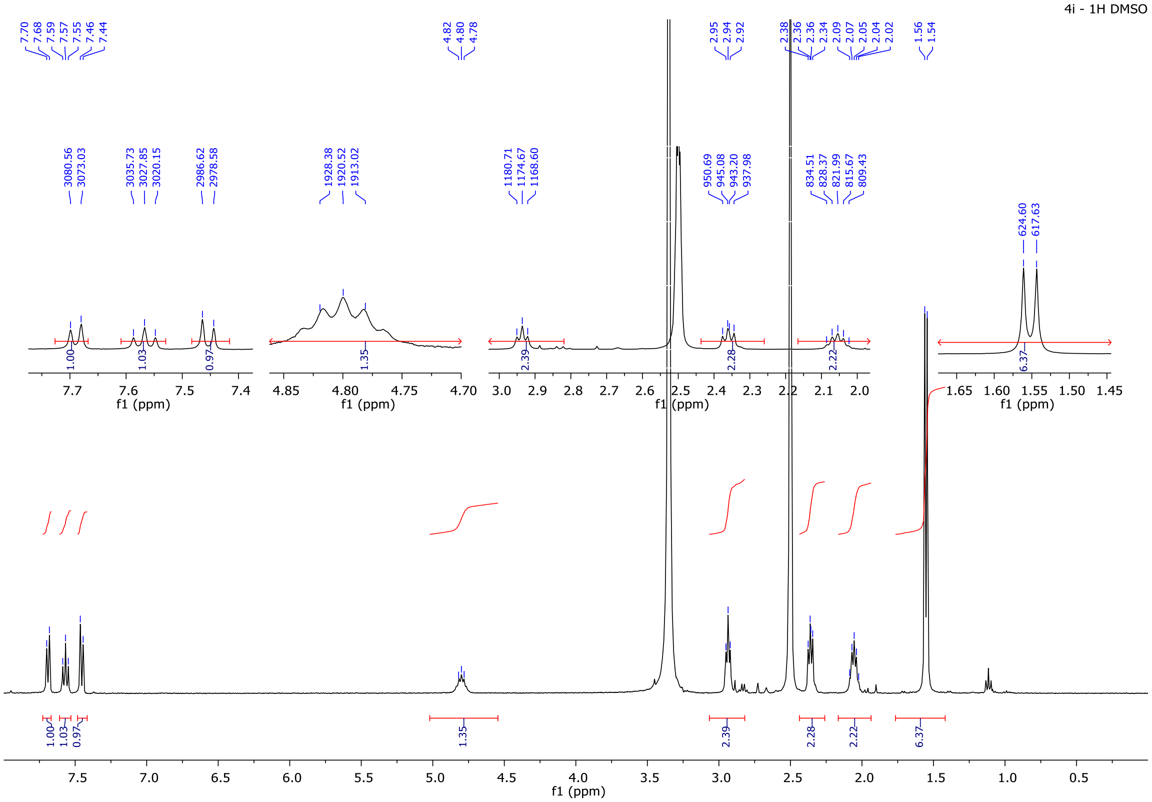

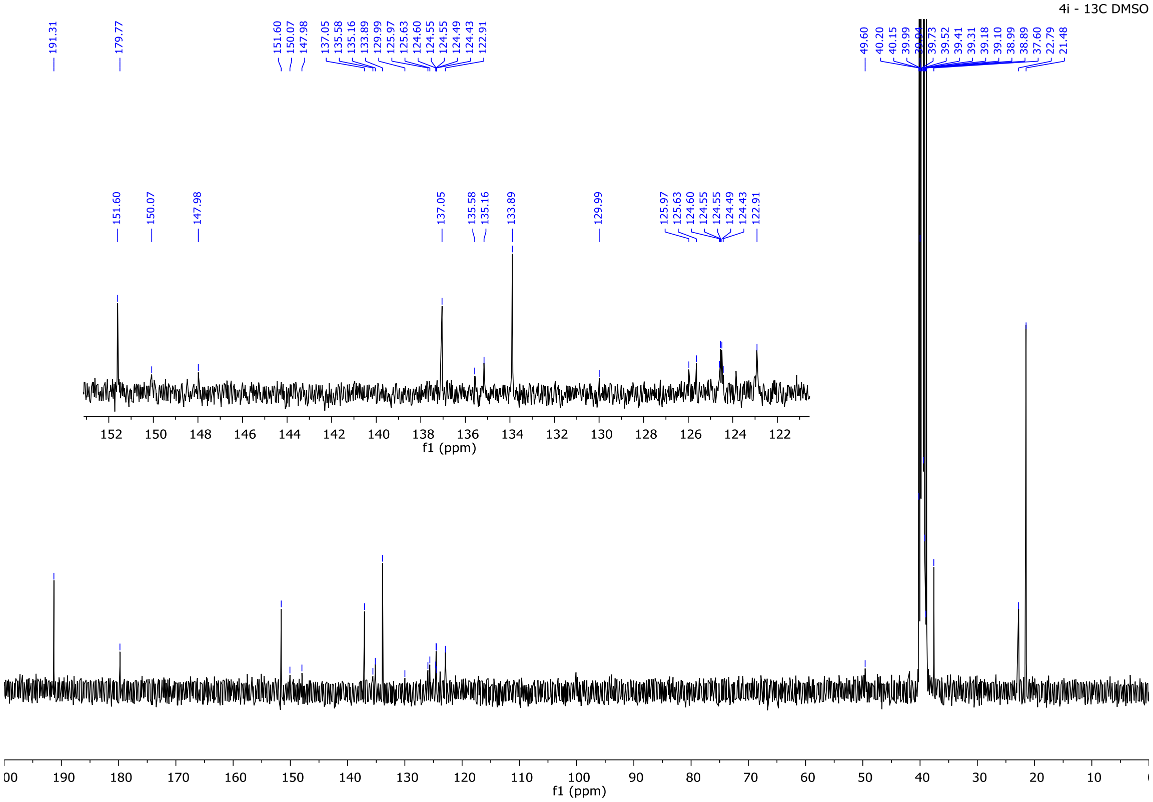

Analysis Info

Analysis Name	Impact2_190708_07_CM3148B.d		
Method	Tune_pos_Standard.m	Acquisition Date	7/8/2019 3:56:23 PM
Comment		Instrument / Ser#	impact II 1825265.10081

Acquisition Parameter

Source Type	ESI	Ion Polarity	Positive	Set Nebulizer	0.3 Bar
Focus	Active	Set Capillary	2000 V	Set Dry Heater	200 °C
Scan Begin	50 m/z	Set End Plate Offset	-500 V	Set Dry Gas	4.0 l/min
Scan End	1200 m/z	Set Collision Cell RF	750.0 Vpp	Set Divert Valve	Source

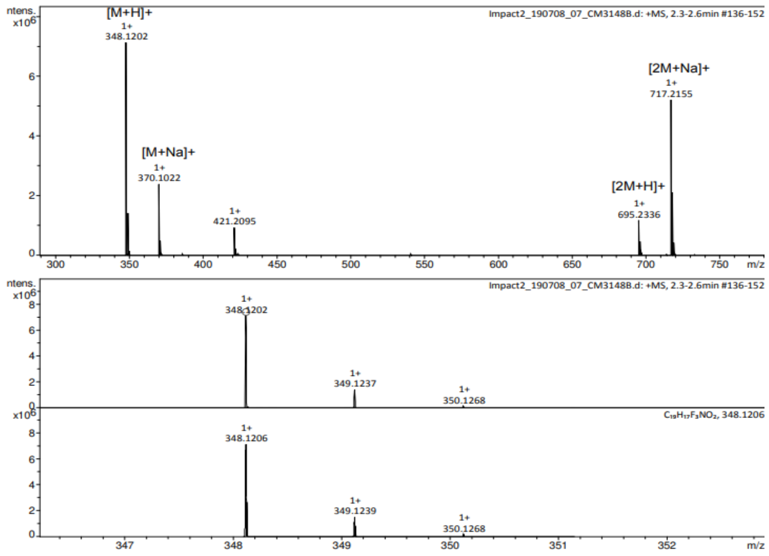

Meas. m/z	Ion Formula	m/z	err [ppm]	mSigma
348.1202	C19H17F3NO2	348.1206	1.1	7.0
370.1022	C19H16F3NNaO2	370.1025	1.0	5.8
695.2336	C38H33F6N2O4	695.2339	0.4	16.0
717.2155	C38H32F6N2NaO4	717.2158	0.5	11.1

1-Bromo-5-isopropyl-5,6,7,8-tetrahydroindeno[1,2-*b*]indole-9,10-dione (4j)

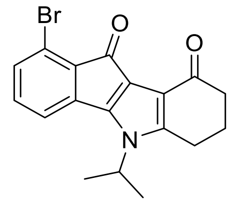

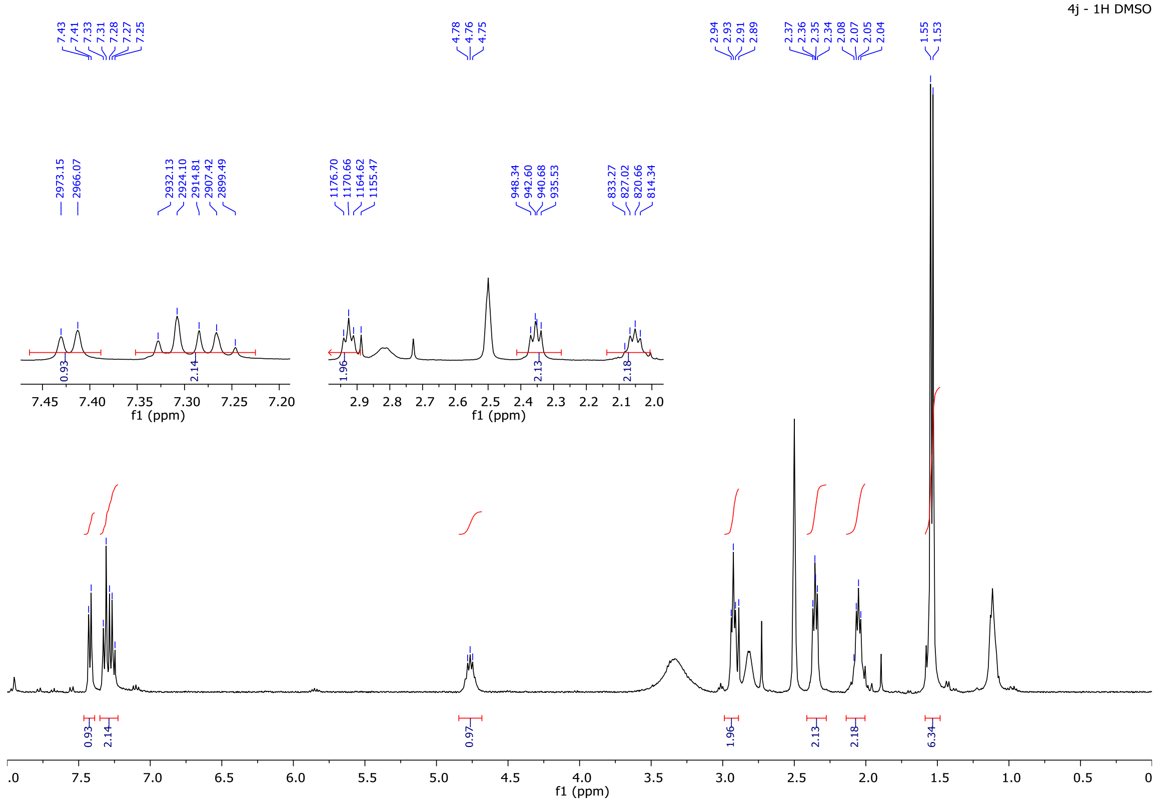

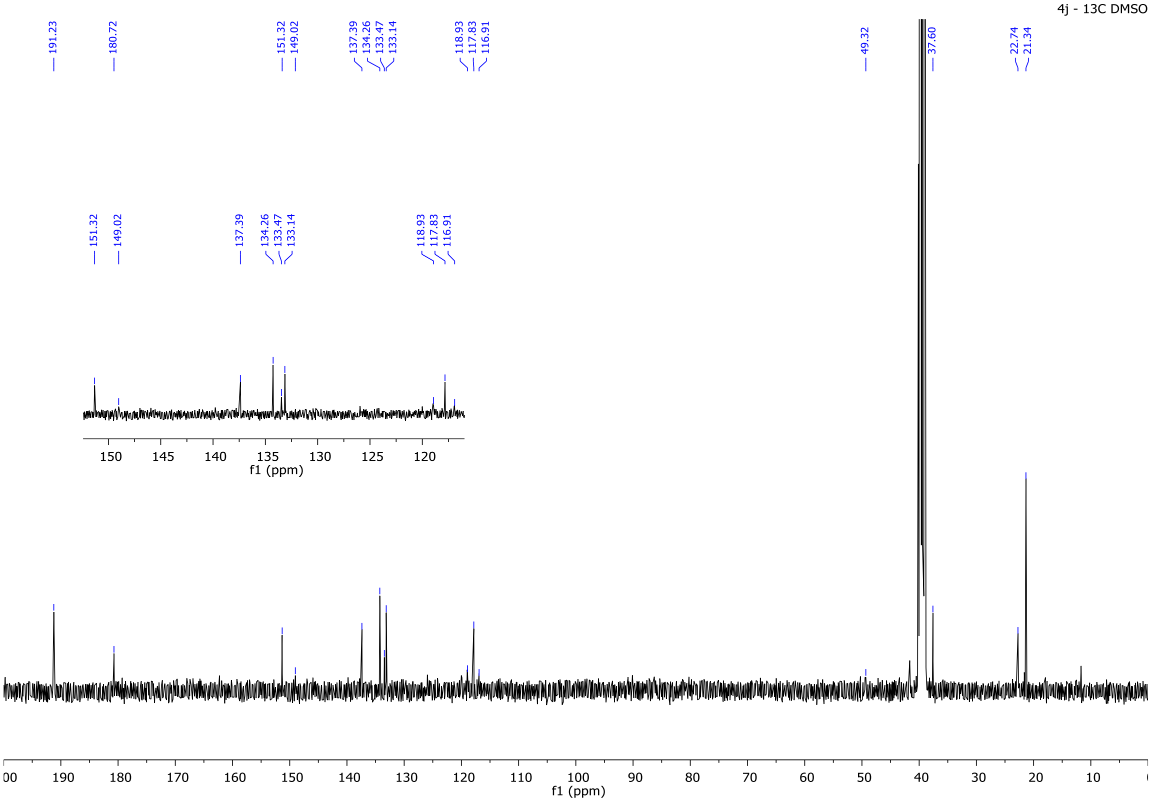
4j - 13C DMSO
191.23
180.72
151.32
149.02
137.39
134.26
133.47
133.14
118.93
117.83
116.91
49.32
37.60
22.74
21.34
f1 (ppm)

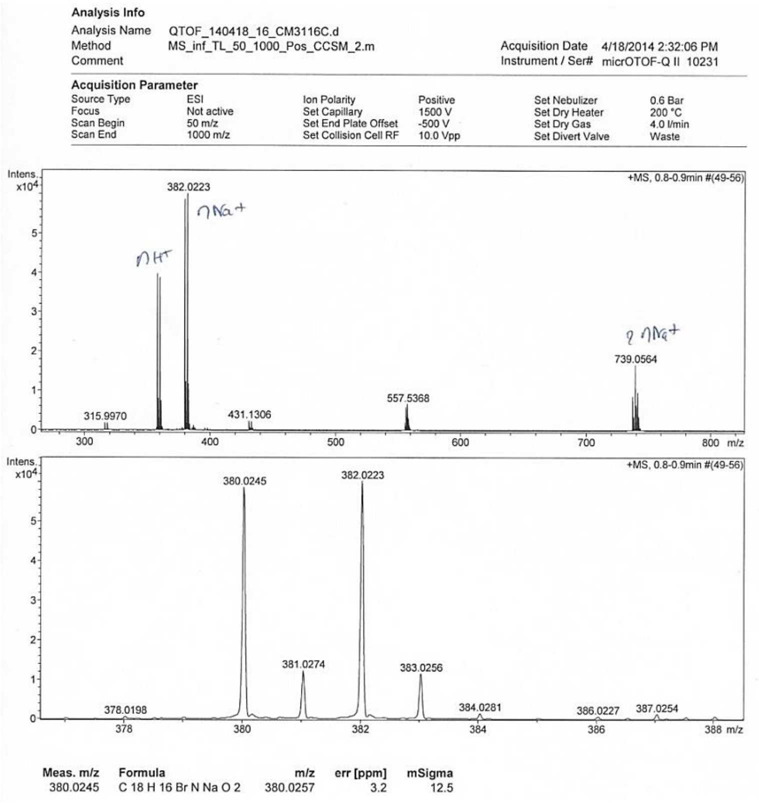
Analysis Info
Analysis Name QTOF_140418_16_CM3116C.d
Method MS_inf_TL_50_1000_Pos_CCSM_2.m
Comment
Acquisition Date 4/18/2014 2:32:06 PM
Instrument / Ser# micrOTOF-Q II 10231
Acquisition Parameter
Source Type ESI
Focus Not active
Scan Begin 50 m/z
Scan End 1000 m/z
Ion Polarity Positive
Set Capillary 1500 V
Set End Plate Offset -500 V
Set Collision Cell RF 10.0 Vpp
Set Nebulizer 0.6 Bar
Set Dry Heater 200 °C
Set Dry Gas 4.0 l/min
Set Divert Valve Waste
+MS, 0.8-0.9min #(49-56)
382.0223
315.9970
431.1306
557.5368
739.0564
380.0245
381.0274
383.0256
378.0198
384.0281
386.0227
387.0254
Meas. m/z Formula m/z err [ppm] mSigma
380.0245 C 18 H 16 Br N Na O 2 380.0257 3.2 12.5

(5-Isopropyl-9,10-dioxo-5,6,7,8,9,10-hexahydroindeno[1,2-b]indol-3-yloxy)acetic acid methyl ester (4p)

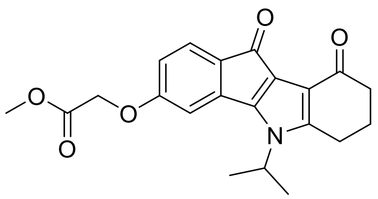

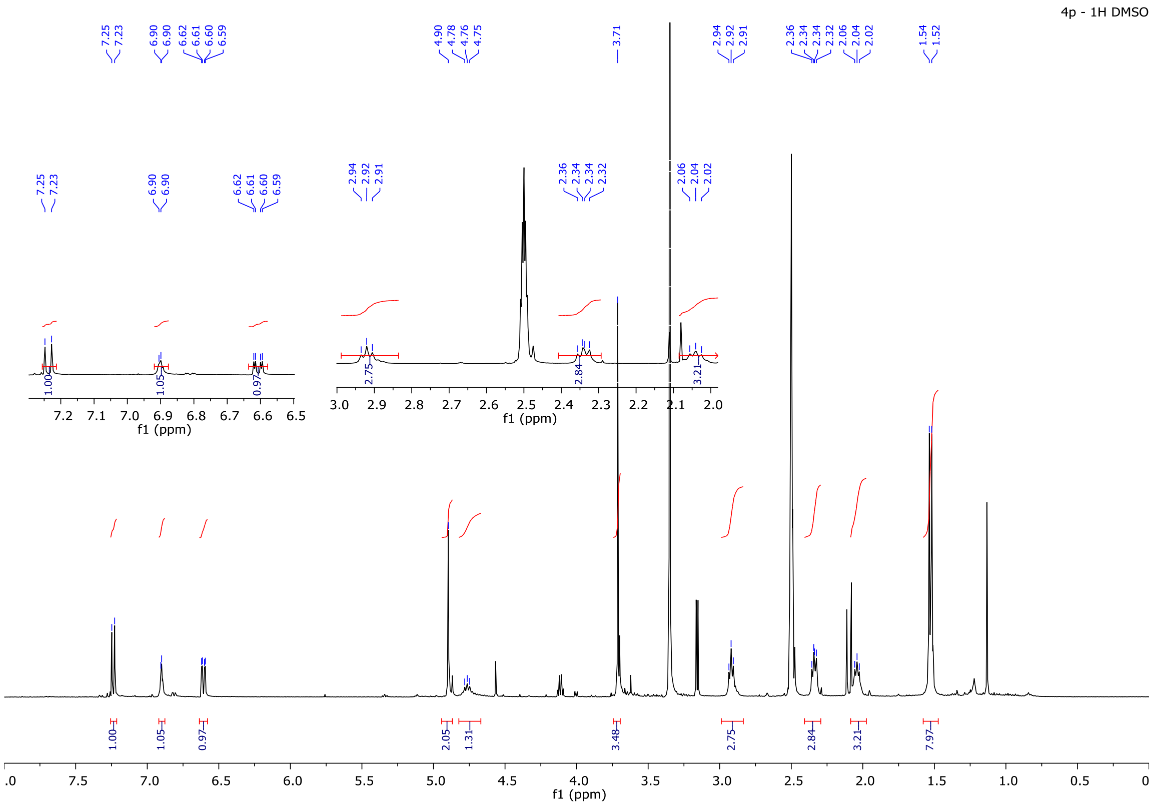

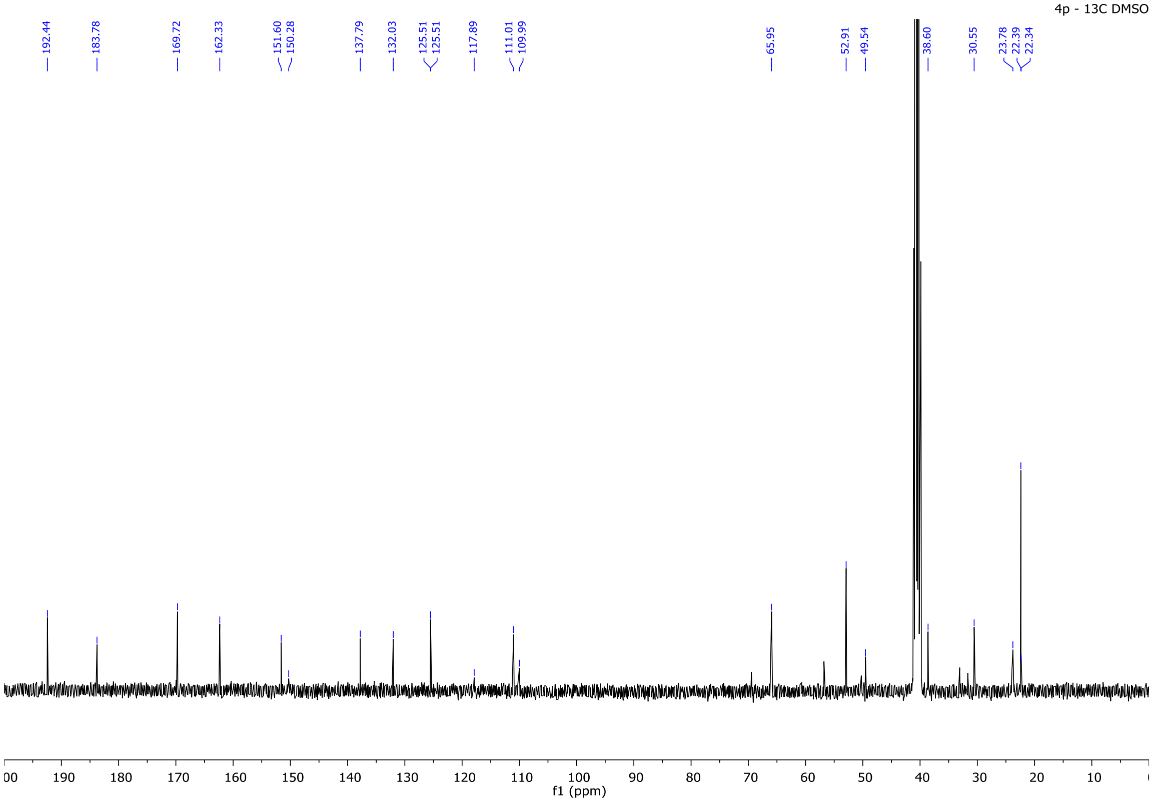

Analysis Info

Analysis Name	QTOF_130619_06_NA20-1.d		
Method	MS_inf_TL_50_1000_Pos_CCSM_2.m	Acquisition Date	6/19/2013 10:54:59 AM
Comment		Instrument / Ser#	micrOTOF-Q II 10231

Acquisition Parameter

Source Type	ESI	Ion Polarity	Positive	Set Nebulizer	0.6 Bar
Focus	Not active	Set Capillary	1500 V	Set Dry Heater	200 °C
Scan Begin	50 m/z	Set End Plate Offset	-500 V	Set Dry Gas	4.0 l/min
Scan End	1000 m/z	Set Collision Cell RF	50.0 Vpp	Set Divert Valve	Waste

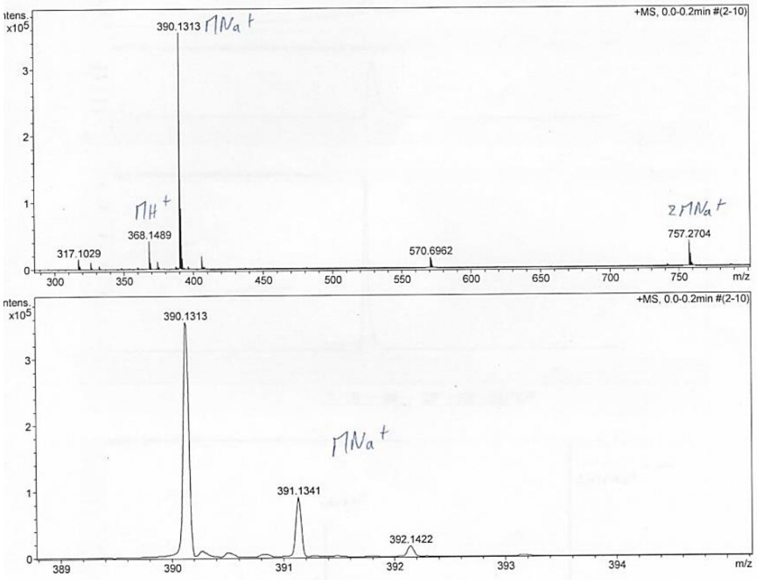

Meas. m/z	Formula	m/z	err [ppm]	mSigma
390.1313	C 21 H 21 N Na O 5	390.1312	-0.2	16.6

5-Isopropyl-9,10-dioxo-5,6,7,8,9,10-hexahydroindeno[1,2-*b*]indol-3-yl acetate (4q)

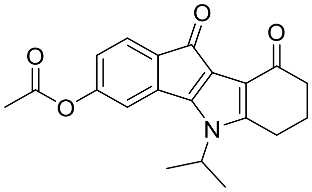

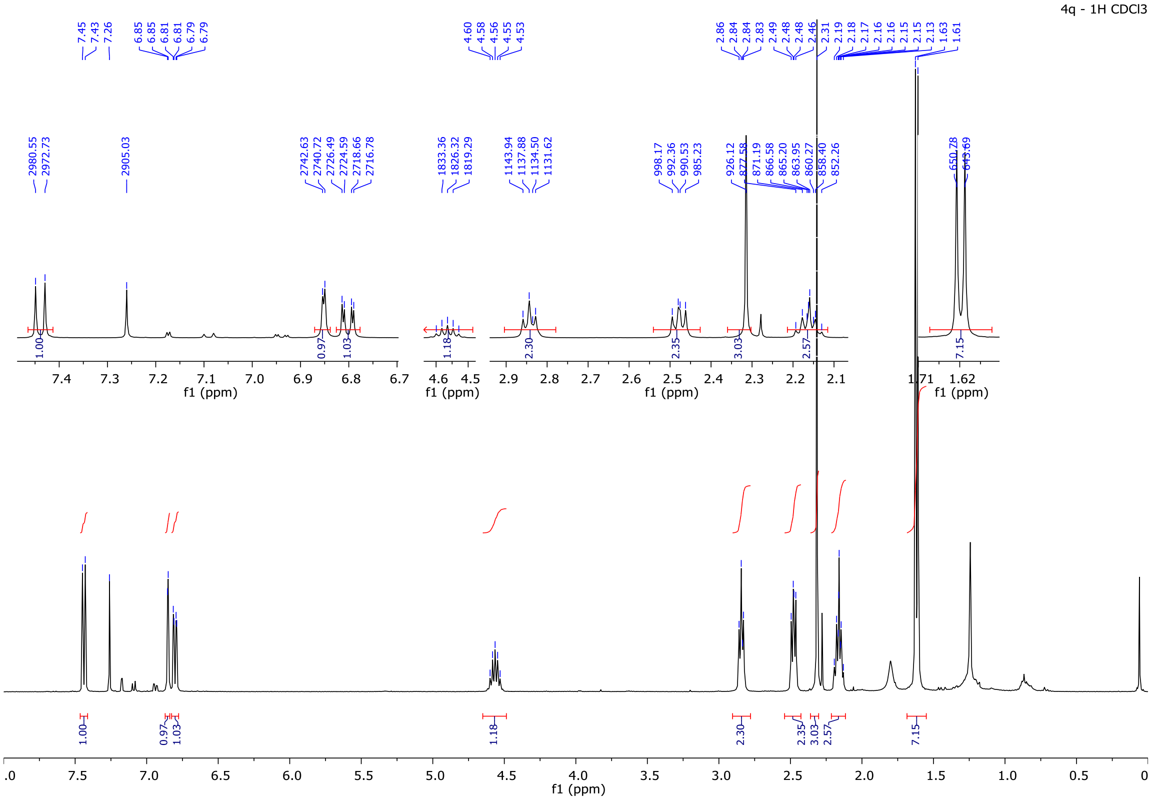

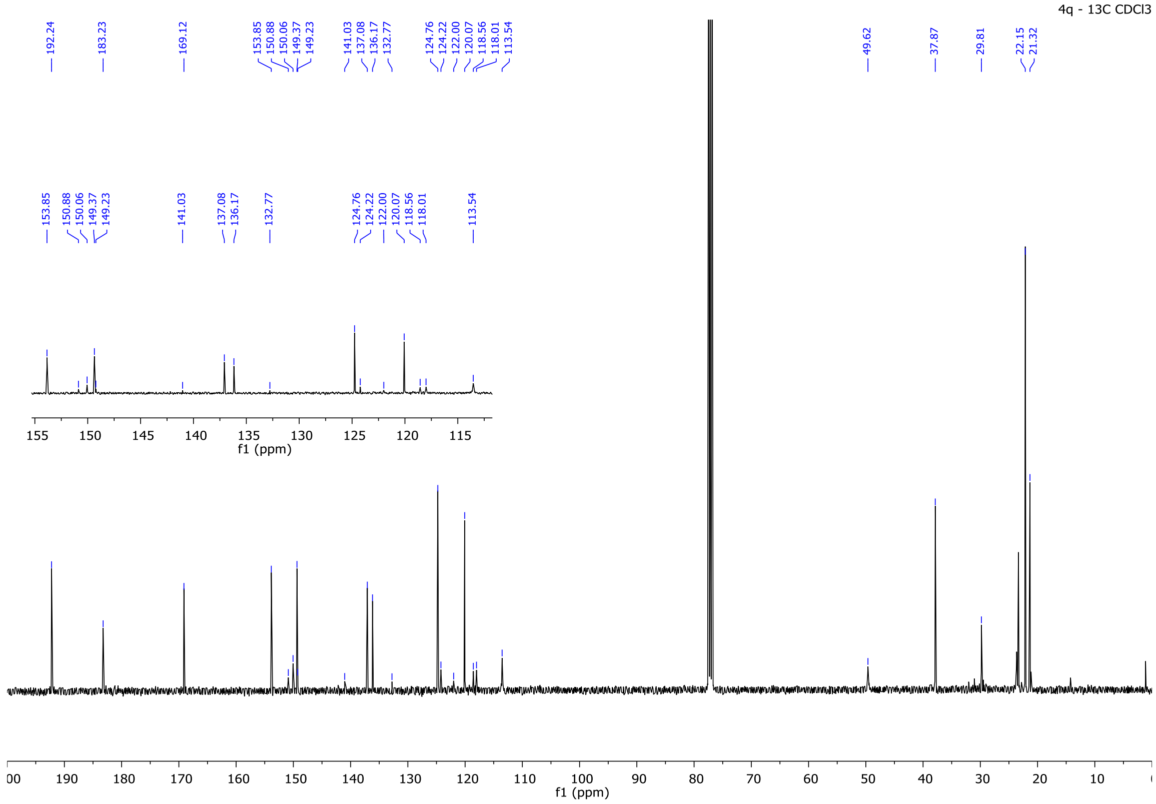
4q - 13C CDCl3
192.24
183.23
169.12
153.85
150.88
150.06
149.37
149.23
141.03
137.08
136.17
132.77
124.76
124.22
122.00
120.07
118.56
118.01
113.54
49.62
37.87
29.81
22.15
21.32
f1 (ppm)

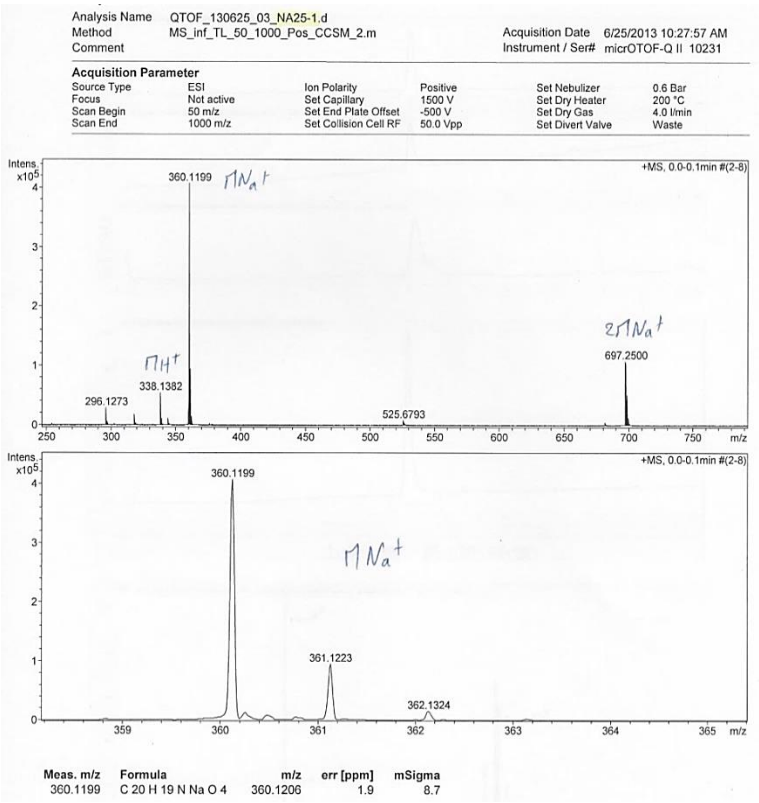
Analysis Name QTOF_130625_03_NA25-1.d
Method MS_inf_TL_50_1000_Pos_CCSM_2.m
Comment
Acquisition Date 6/25/2013 10:27:57 AM
Instrument / Ser# micrOTOF-Q II 10231
Acquisition Parameter
Source Type ESI
Focus Not active
Scan Begin 50 m/z
Scan End 1000 m/z
Ion Polarity Positive
Set Capillary 1500 V
Set End Plate Offset -500 V
Set Collision Cell RF 50.0 Vpp
Set Nebulizer 0.6 Bar
Set Dry Heater 200 °C
Set Dry Gas 4.0 l/min
Set Divert Valve Waste
Intens. x10^5
+MS, 0.0-0.1min #(2-8)
360.1199
MNa+
MH+
338.1382
296.1273
525.6793
2MNa+
697.2500
m/z
361.1223
362.1324
Meas. m/z Formula m/z err [ppm] mSigma
360.1199 C 20 H 19 N Na O 4 360.1206 1.9 8.7

4-(5-Isopropyl-9,10-dioxo-5,6,7,8,9,10-hexahydro-indeno[1,2-*b*]indol-3-yloxy)butyric acid methyl ester (4r)

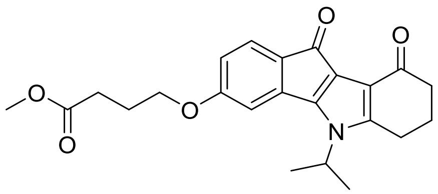

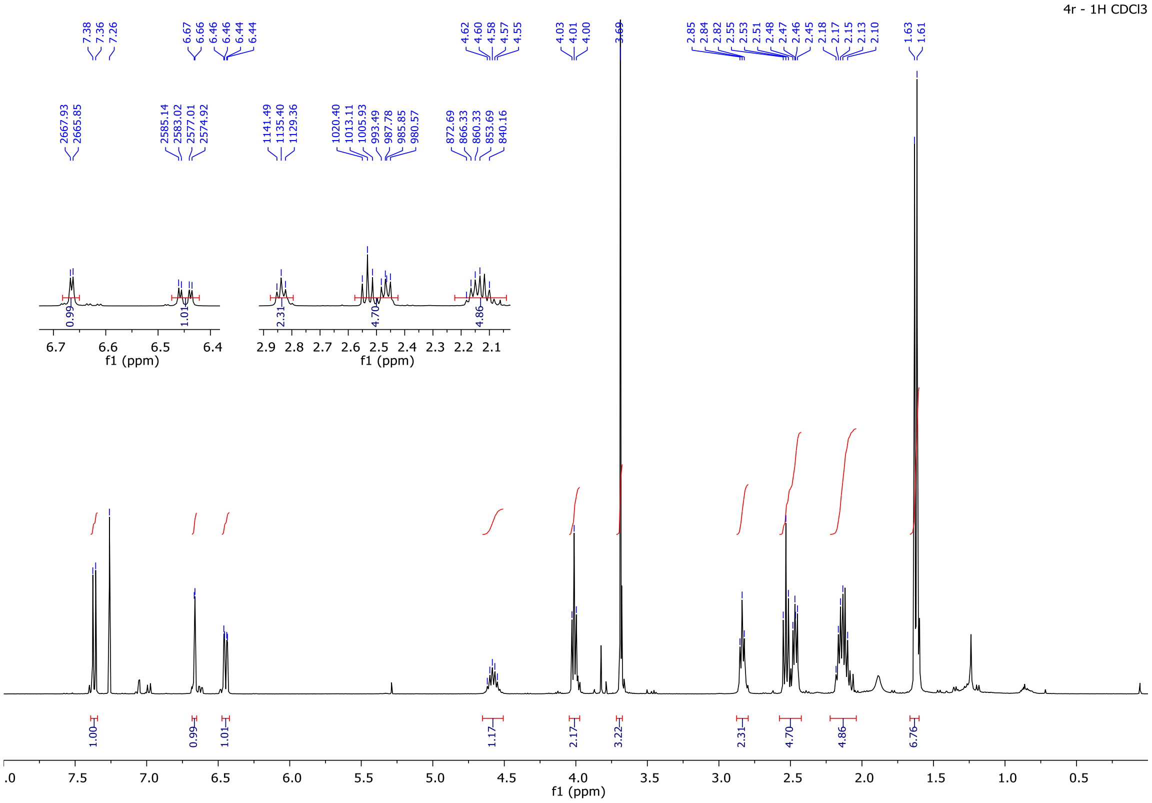

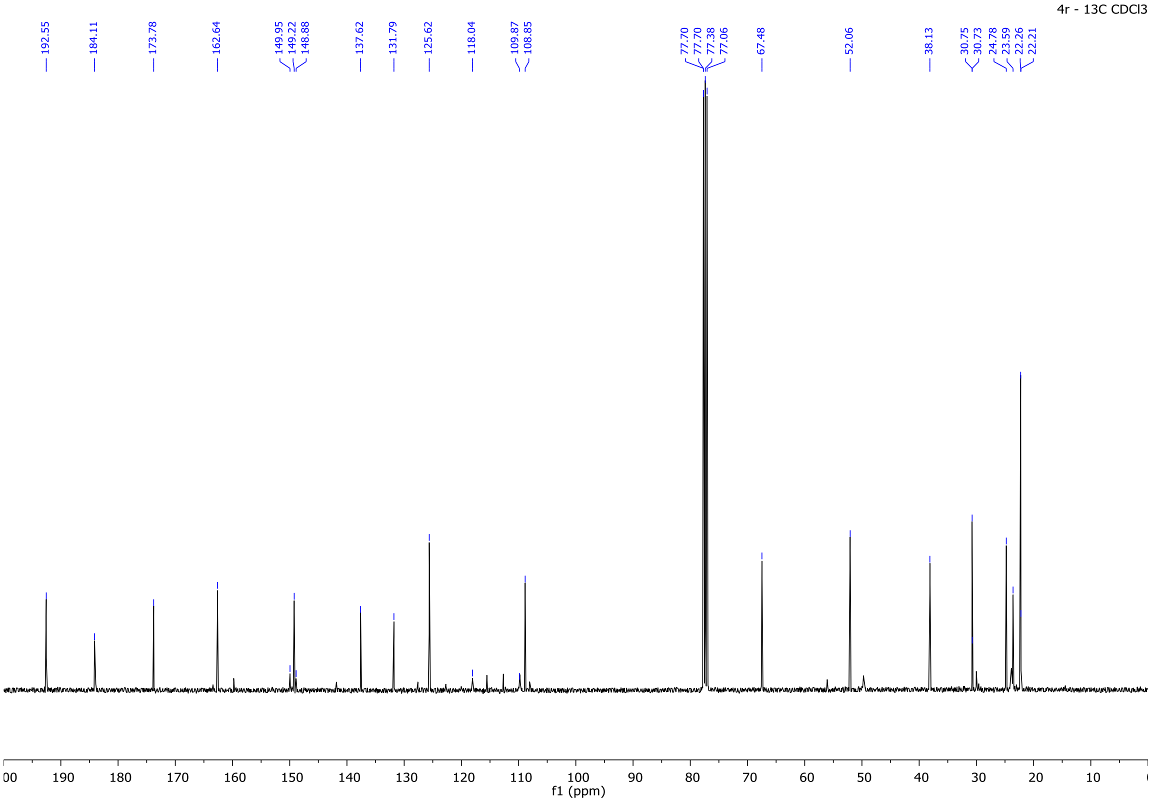
4r - 13C CDCl3
192.55
184.11
173.78
162.64
149.95
149.22
148.88
137.62
131.79
125.62
118.04
109.87
108.85
77.70
77.70
77.38
77.06
67.48
52.06
38.13
30.75
30.73
24.78
23.59
22.26
22.21
f1 (ppm)

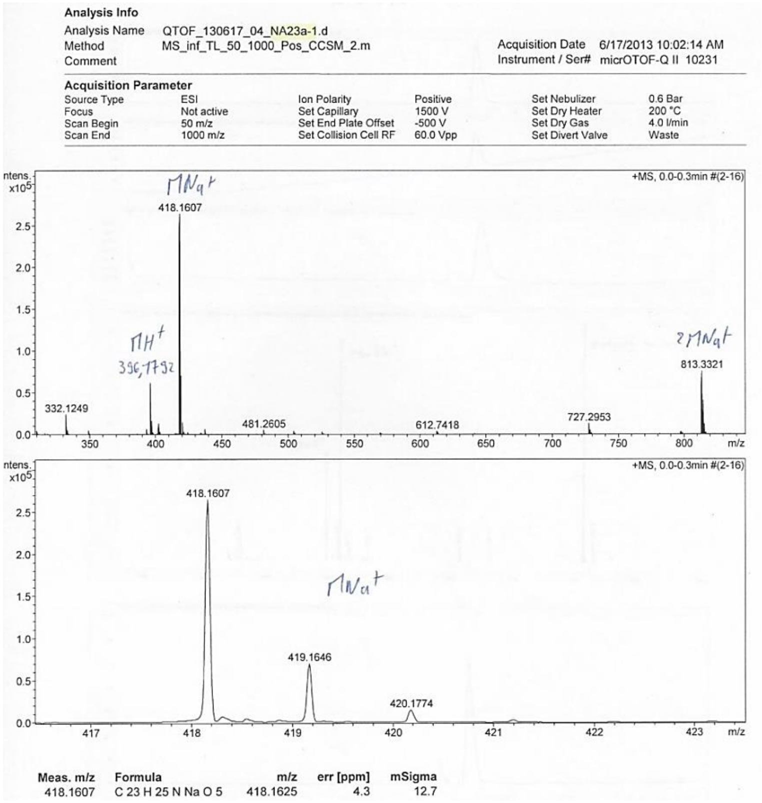
Analysis Info
Analysis Name QTOF_130617_04_NA23a-1.d
Method MS_inf_TL_50_1000_Pos_CCSM_2.m
Comment
Acquisition Date 6/17/2013 10:02:14 AM
Instrument / Ser# micrOTOF-Q II 10231
Acquisition Parameter
Source Type ESI
Focus Not active
Scan Begin 50 m/z
Scan End 1000 m/z
Ion Polarity Positive
Set Capillary 1500 V
Set End Plate Offset -500 V
Set Collision Cell RF 60.0 Vpp
Set Nebulizer 0.6 Bar
Set Dry Heater 200 °C
Set Dry Gas 4.0 l/min
Set Divert Valve Waste
Intens. x10^5
+MS, 0.0-0.3min #(2-16)
MNa+
418.1607
MH+
396,1792
332.1249
481.2605
612.7418
727.2953
2MNa+
813.3321
m/z
+MS, 0.0-0.3min #(2-16)
418.1607
MNa+
419.1646
420.1774
m/z
Meas. m/z Formula m/z err [ppm] mSigma
418.1607 C 23 H 25 N Na O 5 418.1625 4.3 12.7

4-(5-Isopropyl-9,10-dioxo-5,6,7,8,9,10-hexahydroindeno[1,2-*b*]indol-3-yloxy)butyric acid (4s)

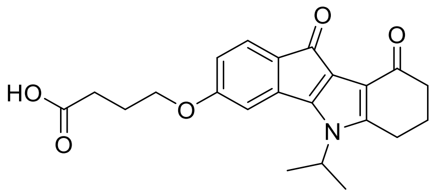

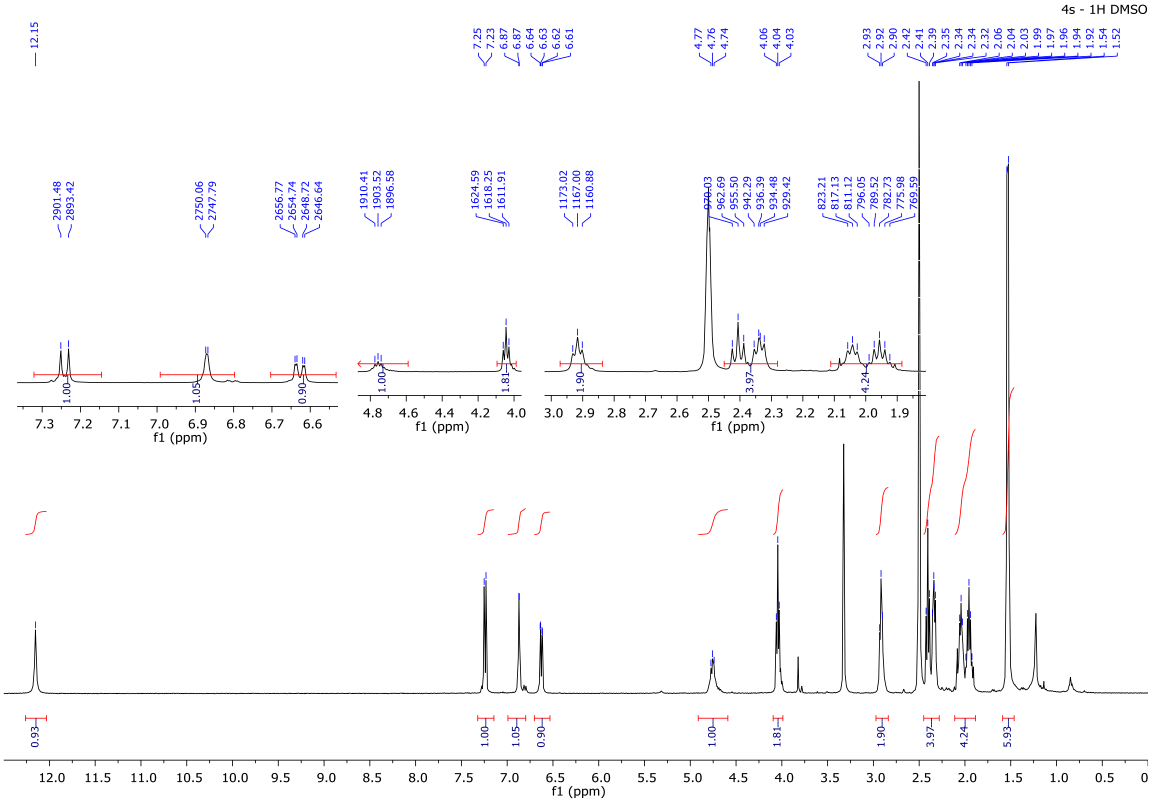

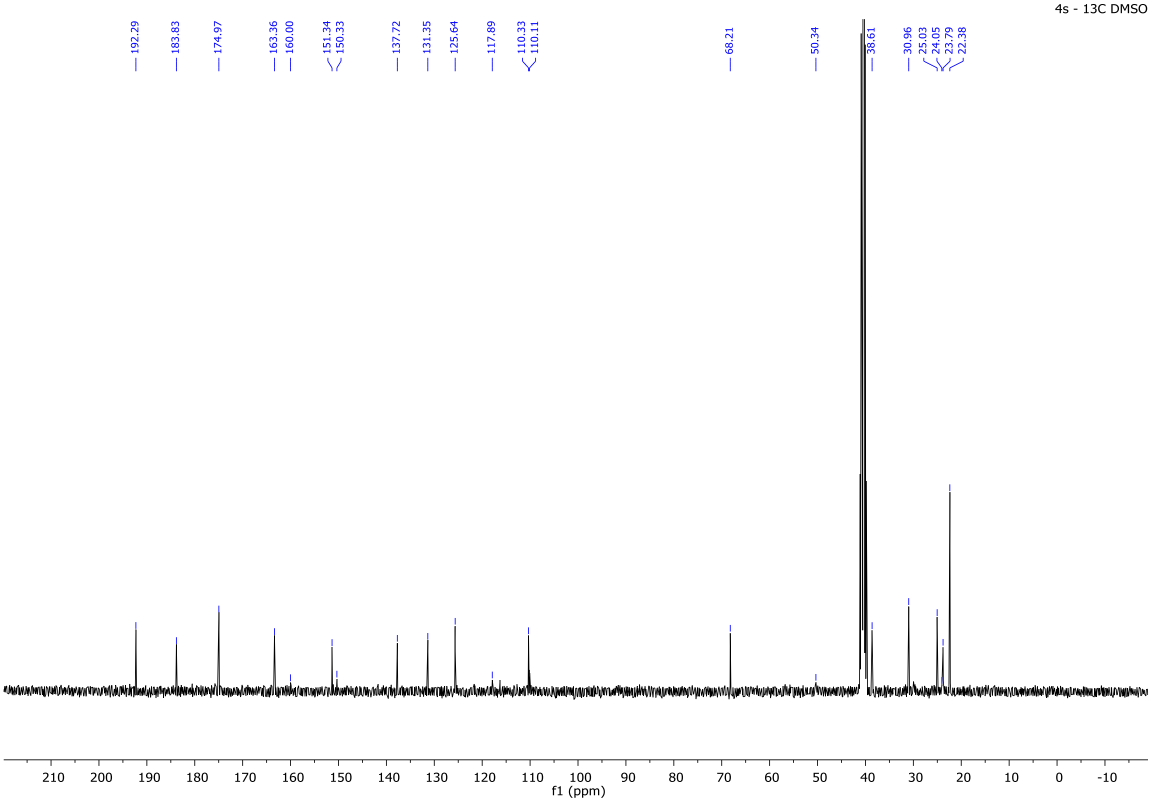

Analysis Info

Analysis Name	QTOF_130625_04_NA29.d		
Method	MS_inf_TL_50_1000_Pos_CCSM_2.m	Acquisition Date	6/25/2013 10:33:19 AM
Comment		Instrument / Ser#	micrOTOF-Q II 10231

Acquisition Parameter

Source Type	ESI	Ion Polarity	Positive	Set Nebulizer	0.6 Bar
Focus	Not active	Set Capillary	1500 V	Set Dry Heater	200 °C
Scan Begin	50 m/z	Set End Plate Offset	-500 V	Set Dry Gas	4.0 l/min
Scan End	1000 m/z	Set Collision Cell RF	50.0 Vpp	Set Divert Valve	Waste

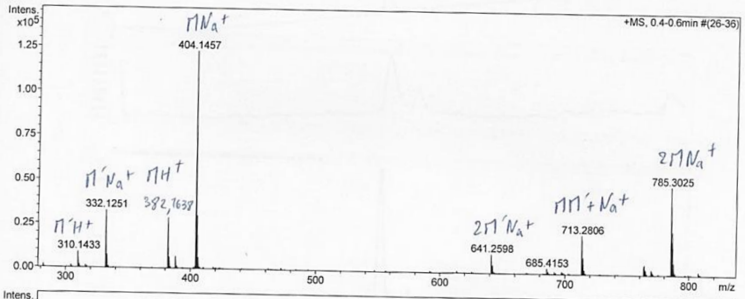

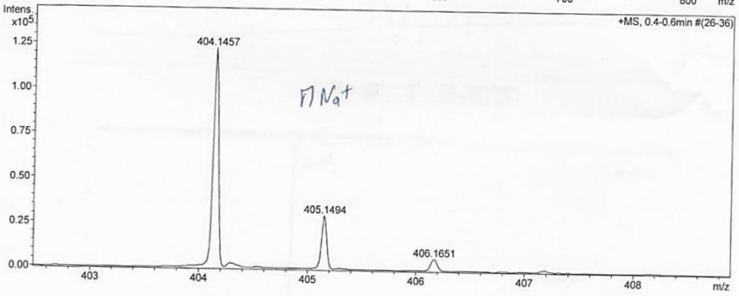

Meas. m/z	Formula	m/z	err [ppm]	mSigma
404.1457	C 22 H 23 N Na O 5	404.1468	2.7	9.6

1,3-Dibromo-5-isopropyl-5,6,7,8-tetrahydroindeno[1,2-*b*]indole-9,10-dione (4w)

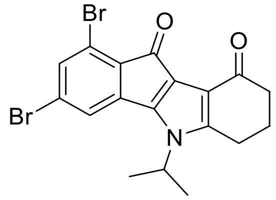

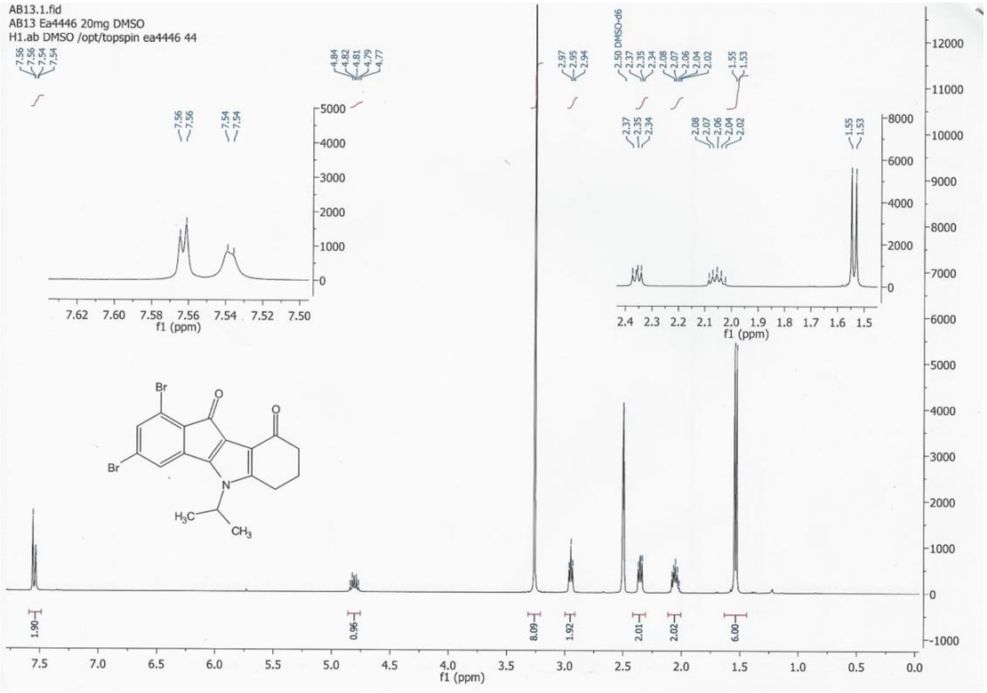

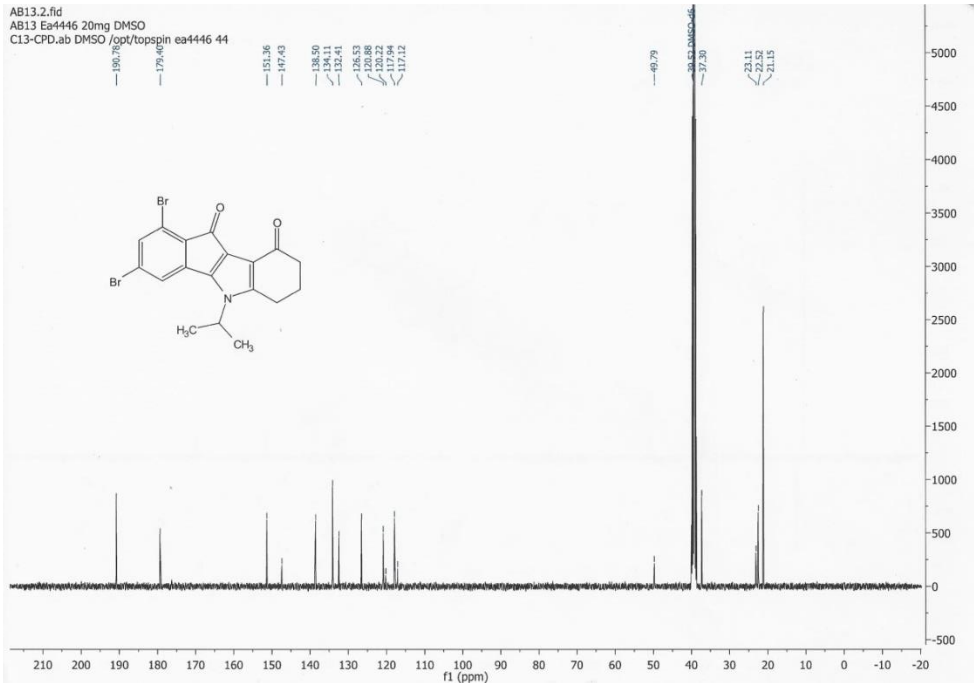

Analysis Info

Analysis Name	QTOF180213_08_AB13.d		
Method	2016_03_17_Infusion_50-1000_pos.m	Acquisition Date	2/13/2018 10:08:17 AM
Comment		Instrument / Ser#	micrOTOF-Q 228888.10231

Acquisition Parameter

Source Type	ESI	Ion Polarity	Positive	Set Nebulizer	0.4 Bar
Focus	Active	Set Capillary	3500 V	Set Dry Heater	200 °C
Scan Begin	50 m/z	Set End Plate Offset	-500 V	Set Dry Gas	4.0 l/min
Scan End	1000 m/z	Set Collision Cell RF	400.0 Vpp	Set Divert Valve	Waste

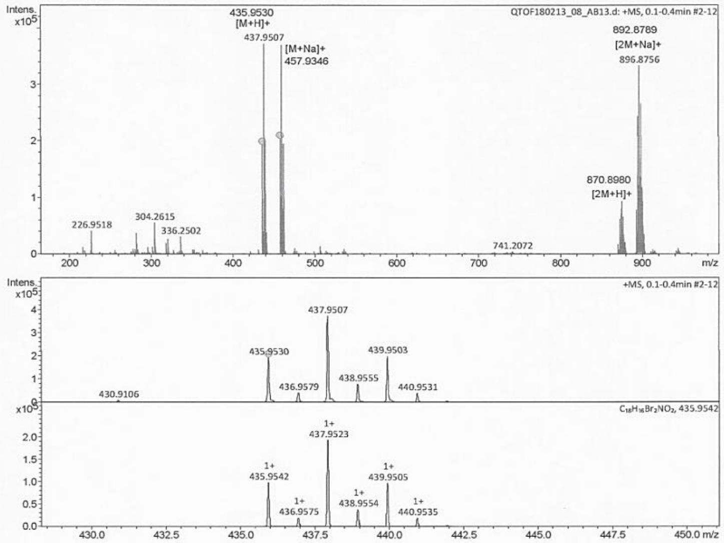

Meas. m/z	Ion Formula	m/z	err [ppm]	mSigma
435.9530	C18H16Br2NO2	435.9542	2.8	13.9
457.9346	C18H15Br2NNaO2	457.9362	3.4	23.1
870.8980	C36H31Br4N2O4	870.9012	3.6	23.3
892.8789	C36H30Br4N2NaO4	892.8831	4.8	77.9

9-Hydroxy-5*H*-indeno[1,2-*b*]indol-10-one (5a)

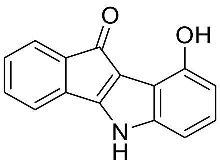

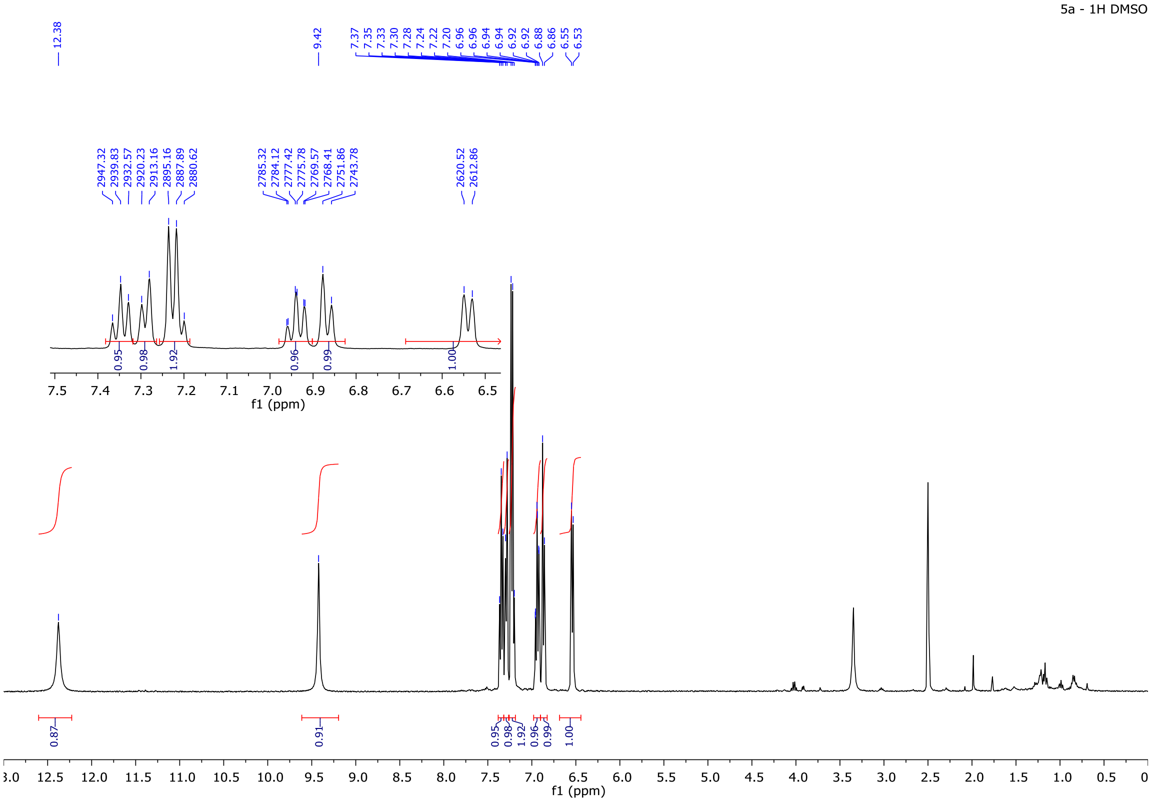

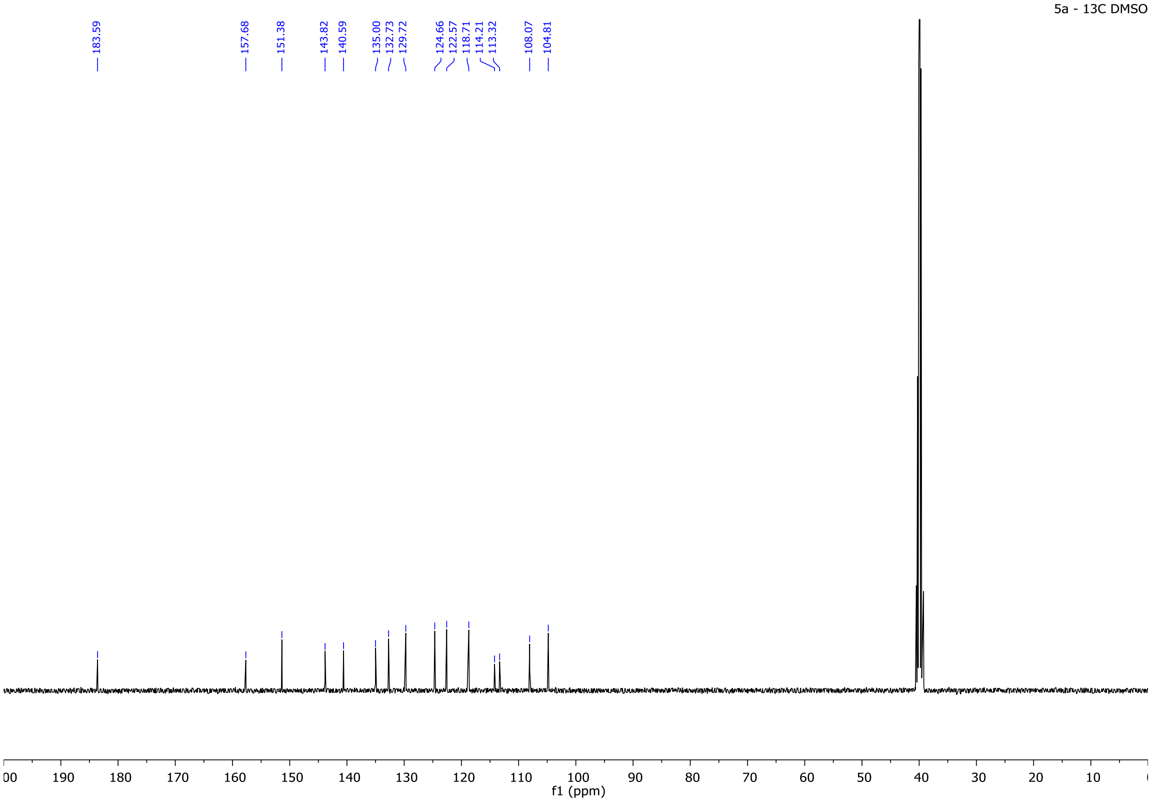

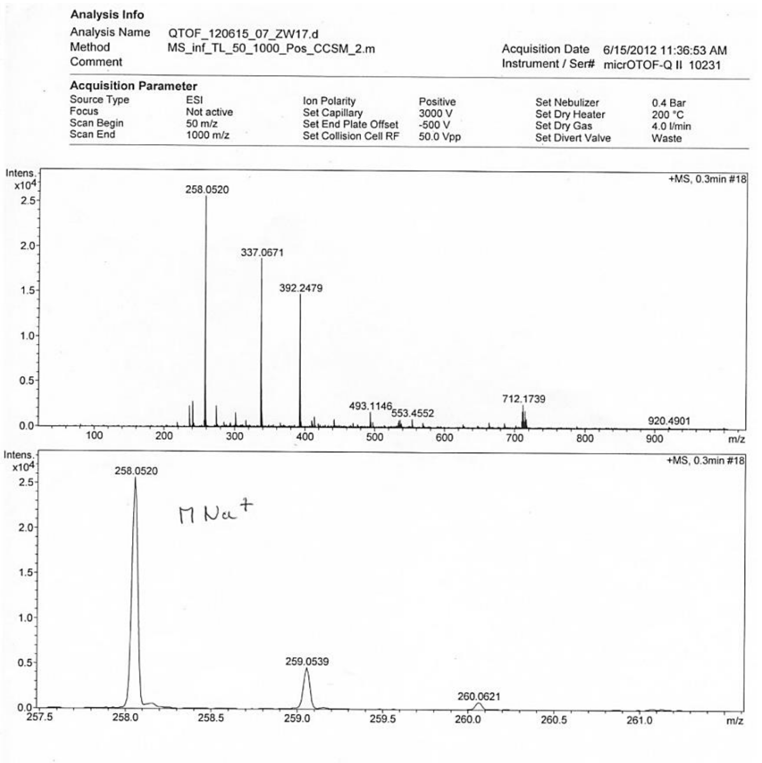

Meas. m/z	Formula	m/z	err [ppm]	mSigma
258.0520	C 15 H 9 N Na O 2	258.0525	2.0	13.6

5-Ethyl-9-hydroxy-5*H*-indeno[1,2-*b*]indol-10-one (5c)

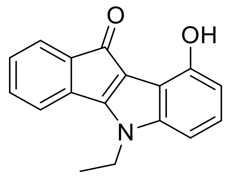

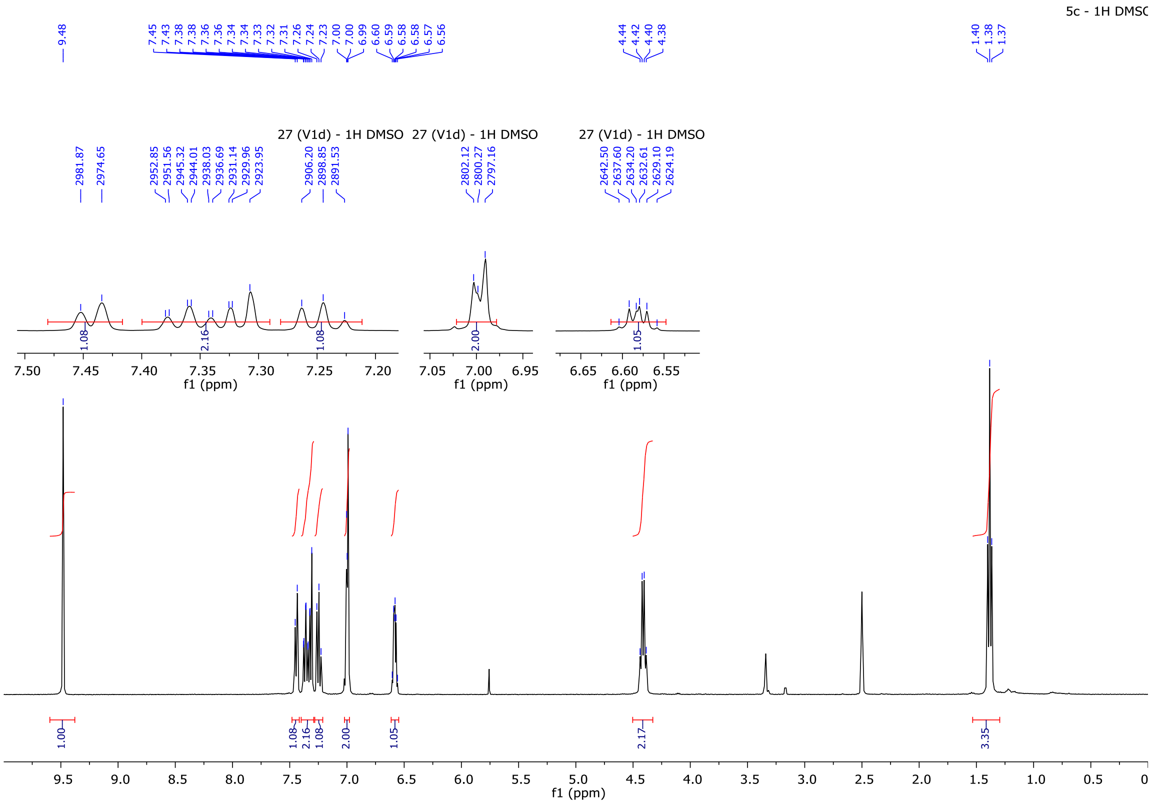

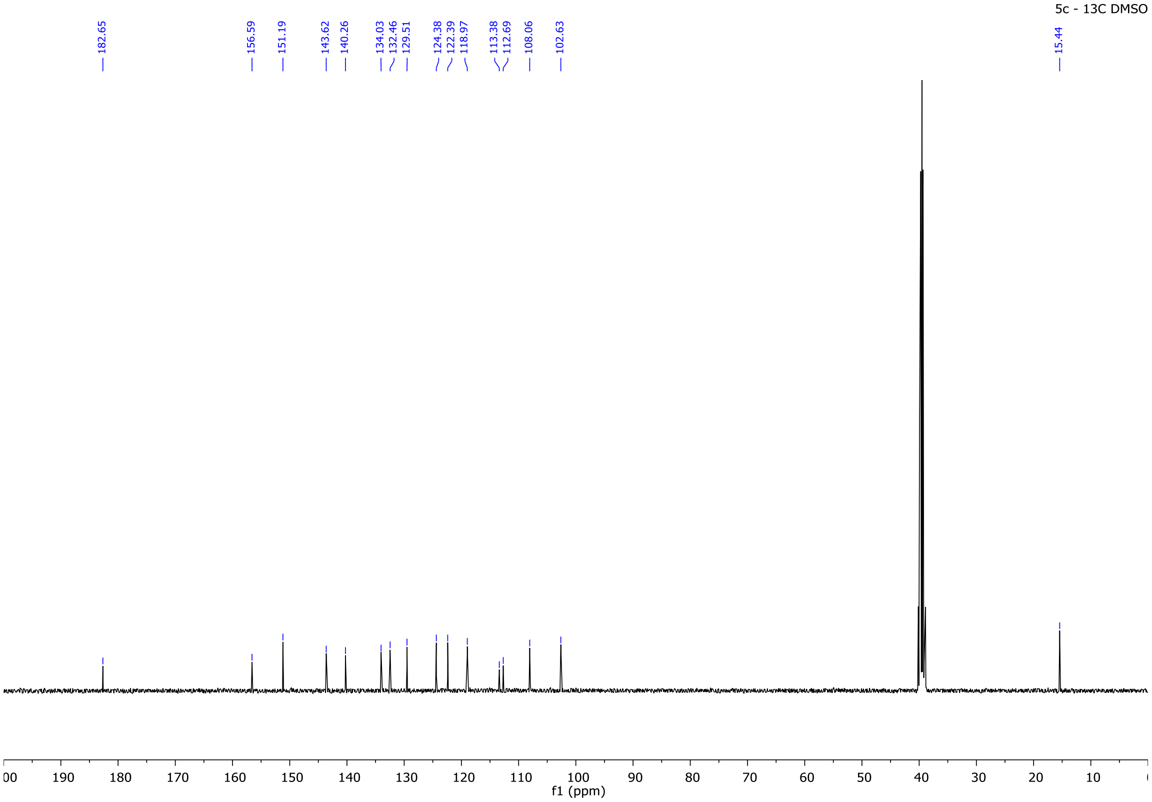

Analysis Info

Analysis Name	QTOF_120615_04_ZW14.d		
Method	MS_inf_TL_50_1000_Pos_CCSM_2.m	Acquisition Date	6/15/2012 11:00:57 AM
Comment		Instrument / Ser#	micrOTOF-Q II 10231

Acquisition Parameter

Source Type	ESI	Ion Polarity	Positive	Set Nebulizer	0.4 Bar		
Focus	Not active	Set Capillary	3000 V	Set Dry Heater	200 °C		
Scan Begin	50 m/z	Set End Plate Offset	-500 V	Set Dry Gas	4.0 l/min		
Scan End	1000 m/z	Set Collision Cell RF	50.0 Vpp	Set Divert Valve	Waste		

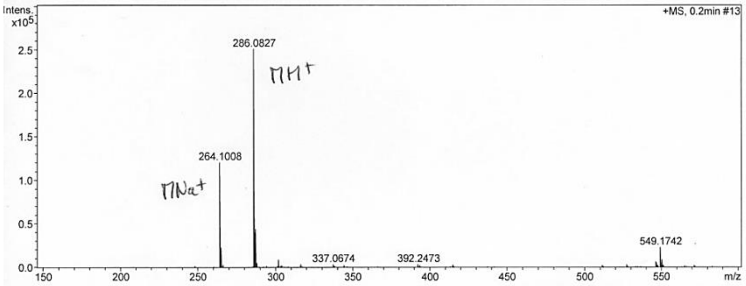

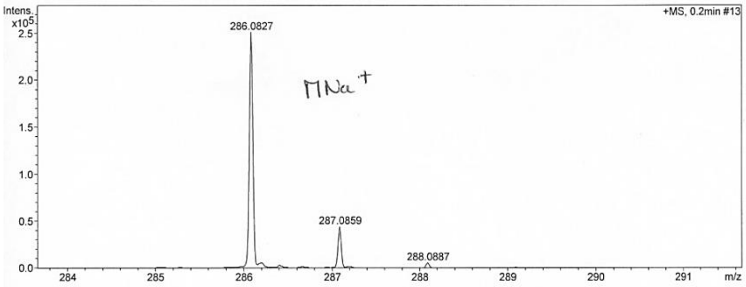

Meas. m/z	Formula	m/z	err [ppm]	mSigma
286.0827	C 17 H 13 N Na O 2	286.0838	4.1	6.5

9-Hydroxy-5-isopropyl-10-oxo-5,10-dihydroindeno[1,2-*b*]indole-1-carbonitrile (5f)

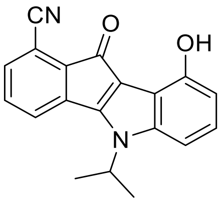

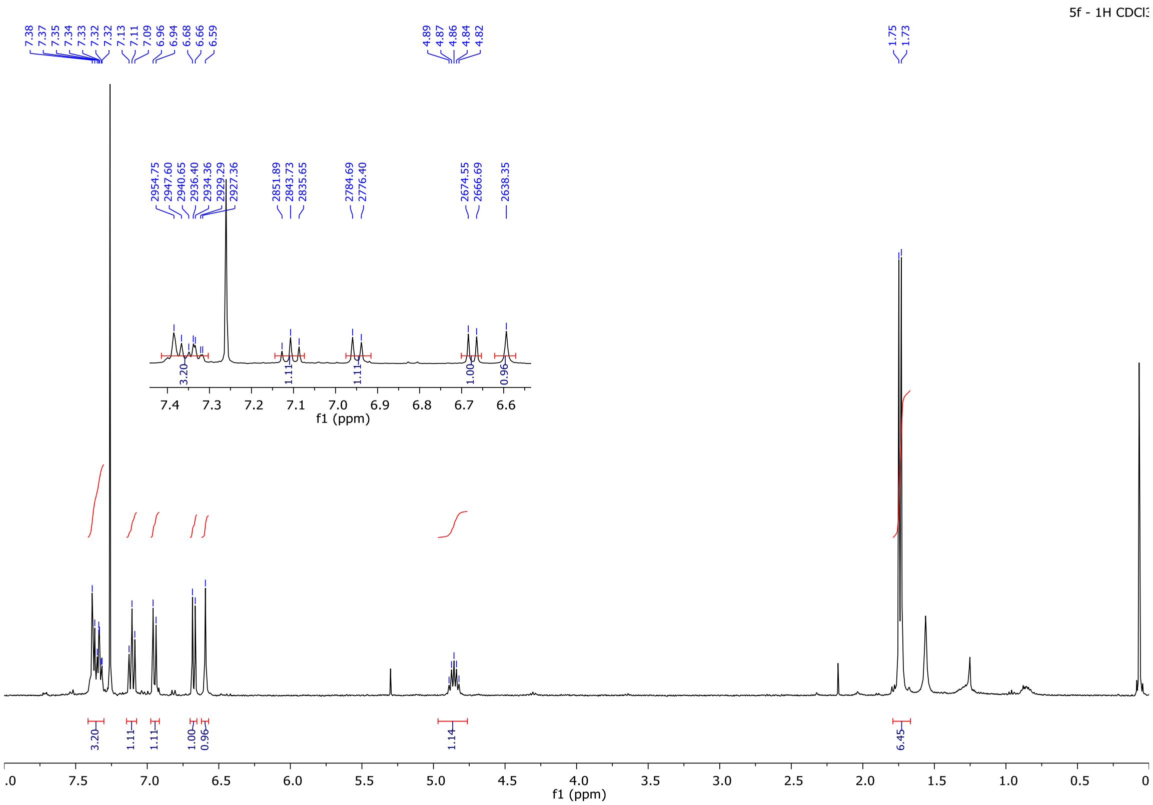

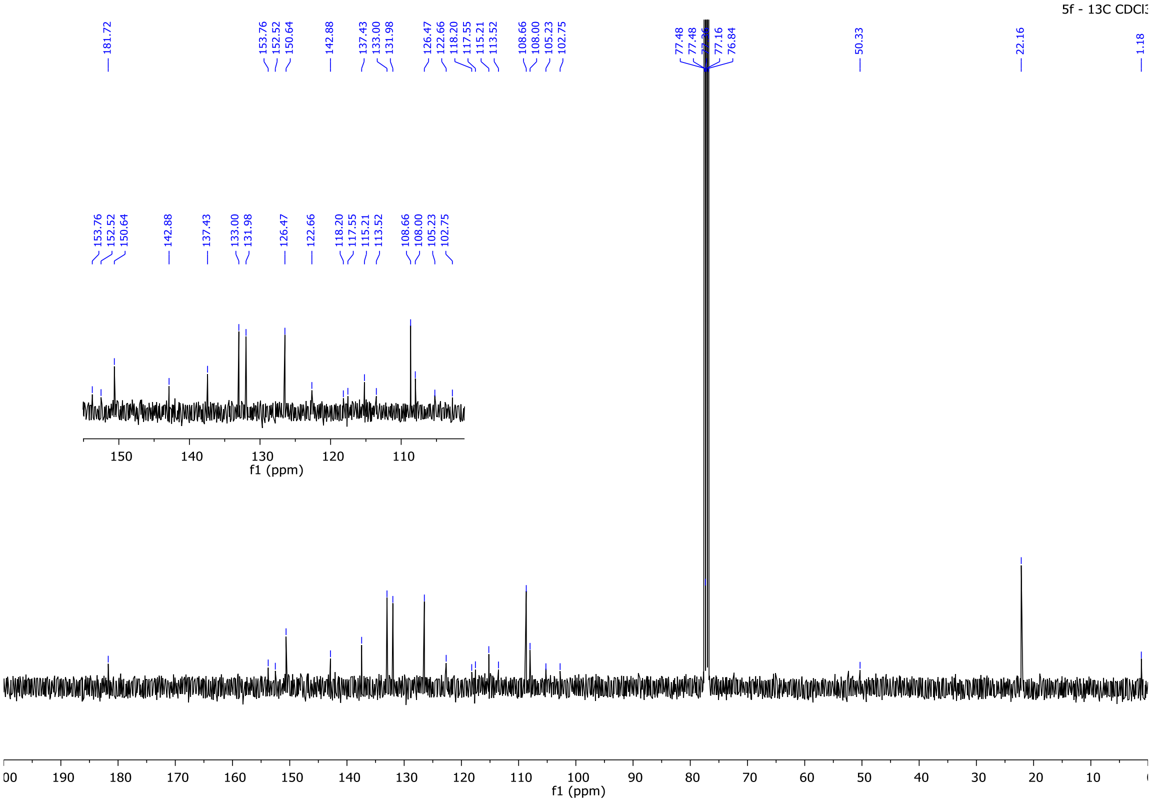

Analysis Info

Analysis Name Impact2_190708_06_CM3132A.d
Method Tune_pos_Standard.m
Comment

Acquisition Date 7/8/2019 3:13:07 PM
Instrument / Ser# impact II 1825265.10081

Acquisition Parameter

Source Type	ESI	Ion Polarity	Positive	Set Nebulizer	0.3 Bar
Focus	Active	Set Capillary	4500 V	Set Dry Heater	200 °C
Scan Begin	50 m/z	Set End Plate Offset	-500 V	Set Dry Gas	4.0 l/min
Scan End	1200 m/z	Set Collision Cell RF	750.0 Vpp	Set Divert Valve	Source

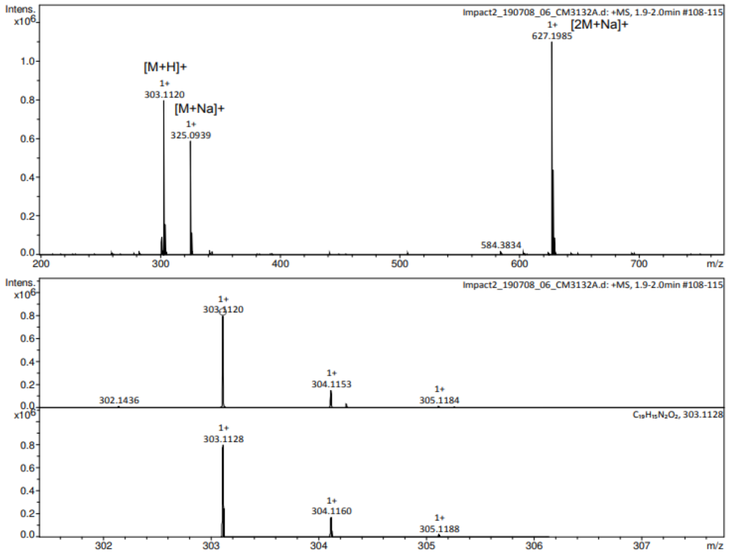

Meas. m/z	Ion Formula	m/z	err [ppm]	mSigma
303.1120	C19H15N2O2	303.1128	2.7	12.0
325.0939	C19H14N2NaO2	325.0947	2.6	12.9
627.1985	C38H28N4NaO4	627.2003	2.8	17.8

1,9-Dihydroxy-5-isopropyl-5*H*-indeno[1,2-*b*]indol-10-one (5g)

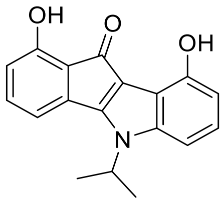

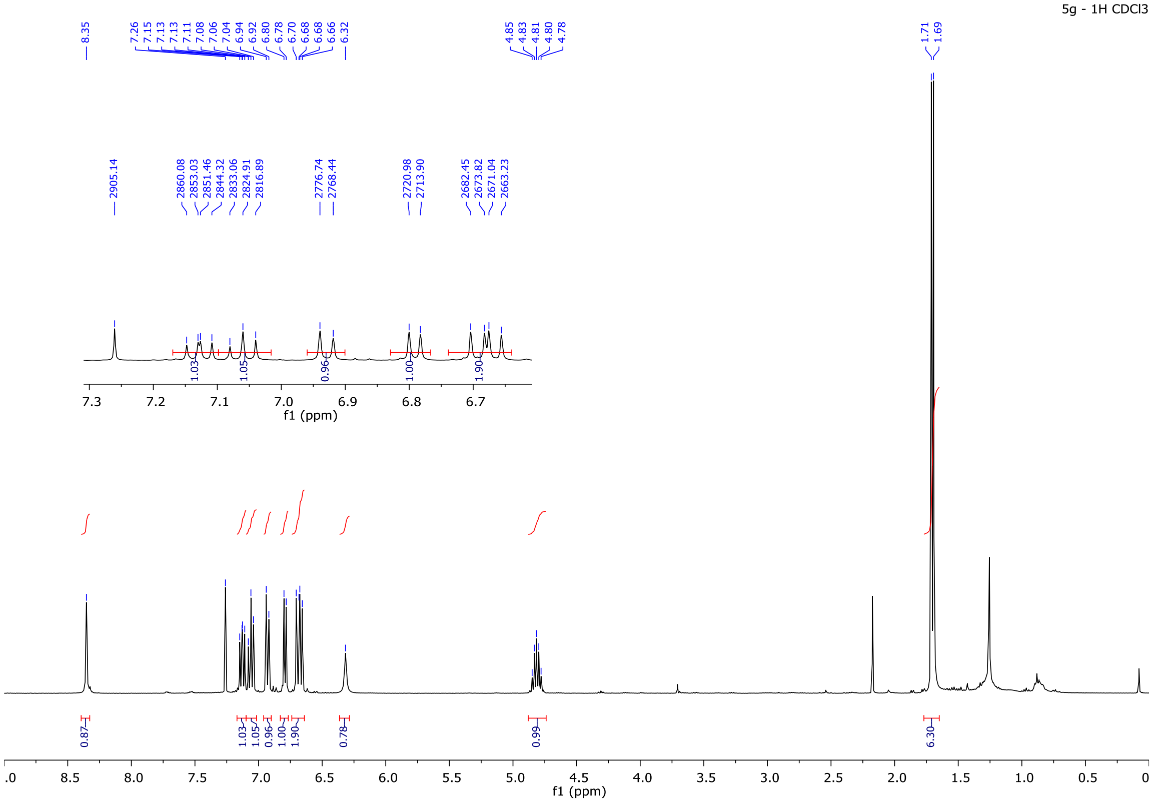

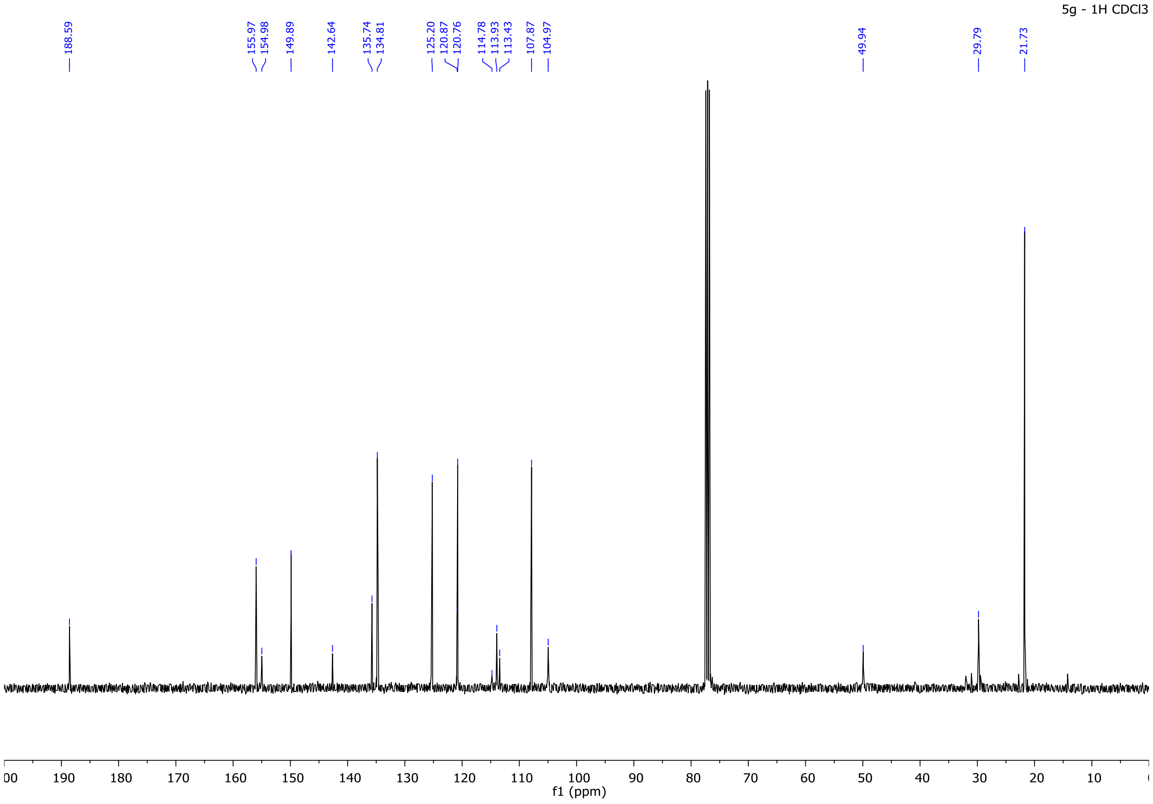

Analysis Info

Analysis Name	QTOF180213_05_CM4017A.d		
Method	2016_03_17_Infusion_50-1000_pos.m	Acquisition Date	2/13/2018 9:18:38 AM
Comment		Instrument / Ser#	micrOTOF-Q 228888.10231

Acquisition Parameter

Source Type	ESI	Ion Polarity	Positive	Set Nebulizer	0.4 Bar
Focus	Active	Set Capillary	1500 V	Set Dry Heater	200 °C
Scan Begin	50 m/z	Set End Plate Offset	-500 V	Set Dry Gas	4.0 l/min
Scan End	1000 m/z	Set Collision Cell RF	400.0 Vpp	Set Divert Valve	Waste

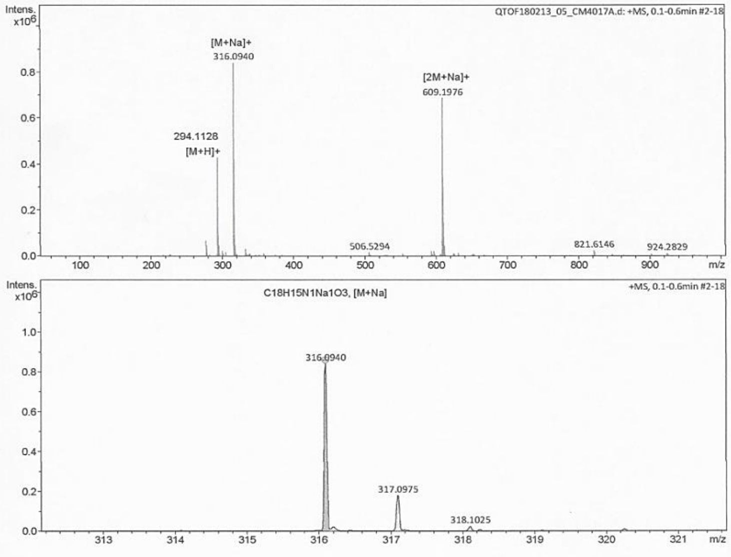

Meas. m/z	Ion Formula	Sum Formula	m/z	err [ppm]	mSigma	z	Adduct
294.1128	C18H16NO3	C18H15NO3	294.1125	-1.1	5.9	1+	M+H
316.0940	C18H15NNaO3		316.0944	1.3	8.2	1+	M+Na
609.1976	C36H30N2NaO6	C36H30N2O6	609.1996	3.3	21.6	1+	M+Na

9-Hydroxy-5-isopropyl-3-methoxy-5*H*-indeno[1,2-*b*]indol-10-one (5h)

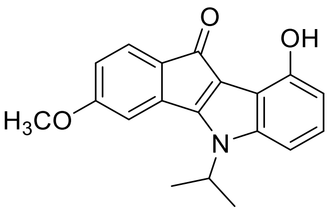

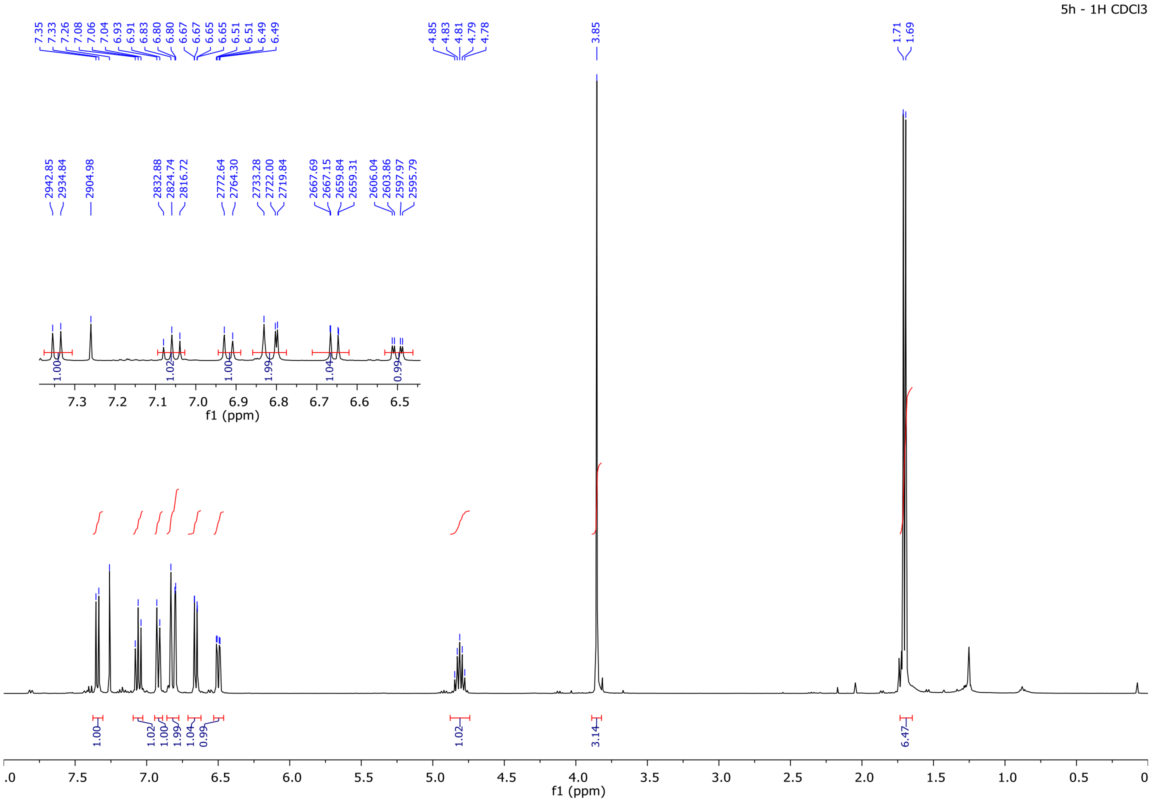

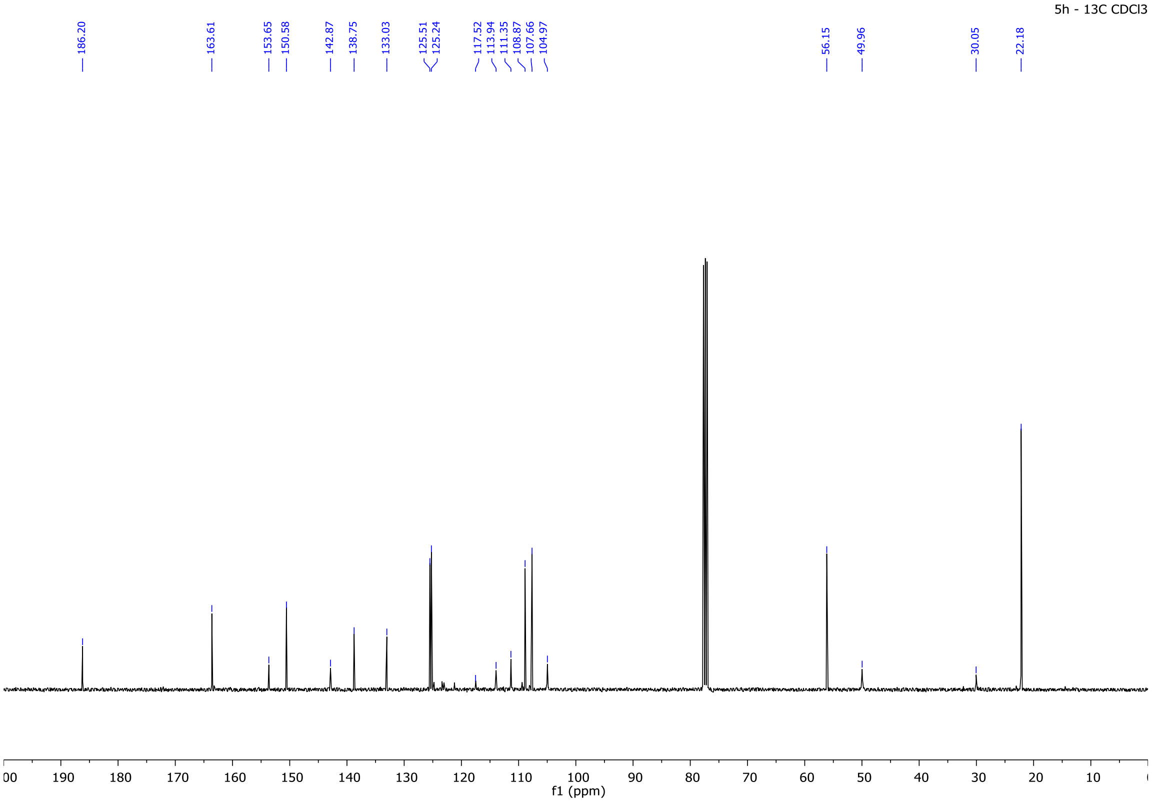

Analysis Name QTOF_130710_20_NA32.d
Method MS_inf_TL_50_1000_Pos_CCSM_2.m
Comment

Acquisition Date 7/10/2013 4:29:18 PM
Instrument / Ser# micrOTOF-Q II 10231

Acquisition Parameter

Source Type	ESI	Ion Polarity	Positive	Set Nebulizer	0.6 Bar
Focus	Not active	Set Capillary	1500 V	Set Dry Heater	200 °C
Scan Begin	50 m/z	Set End Plate Offset	-500 V	Set Dry Gas	4.0 l/min
Scan End	1000 m/z	Set Collision Cell RF	50.0 Vpp	Set Divert Valve	Waste

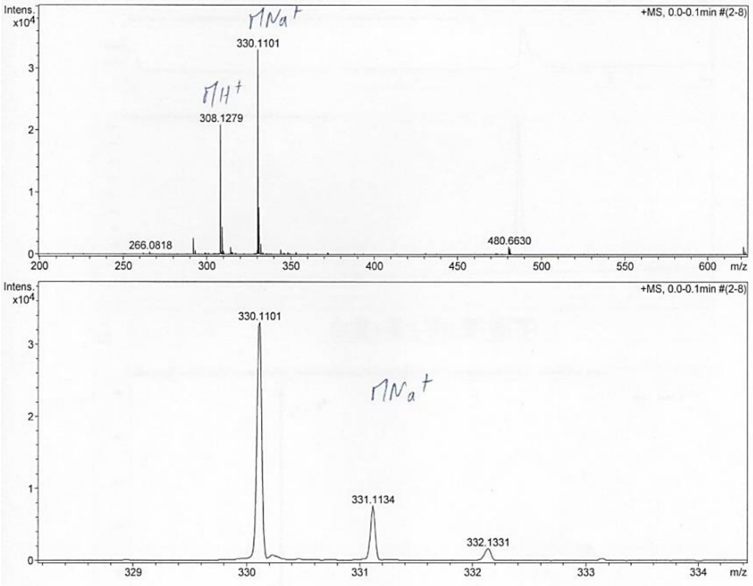

Meas. m/z	Formula	m/z	err [ppm]	mSigma
330.1101	C 19 H 17 N Na O 3	330.1101	-0.1	18.3

5*H*-Indeno[1,2-*b*]indole-6,9,10-trione (6a)

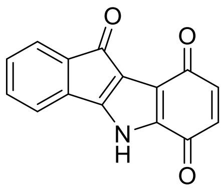

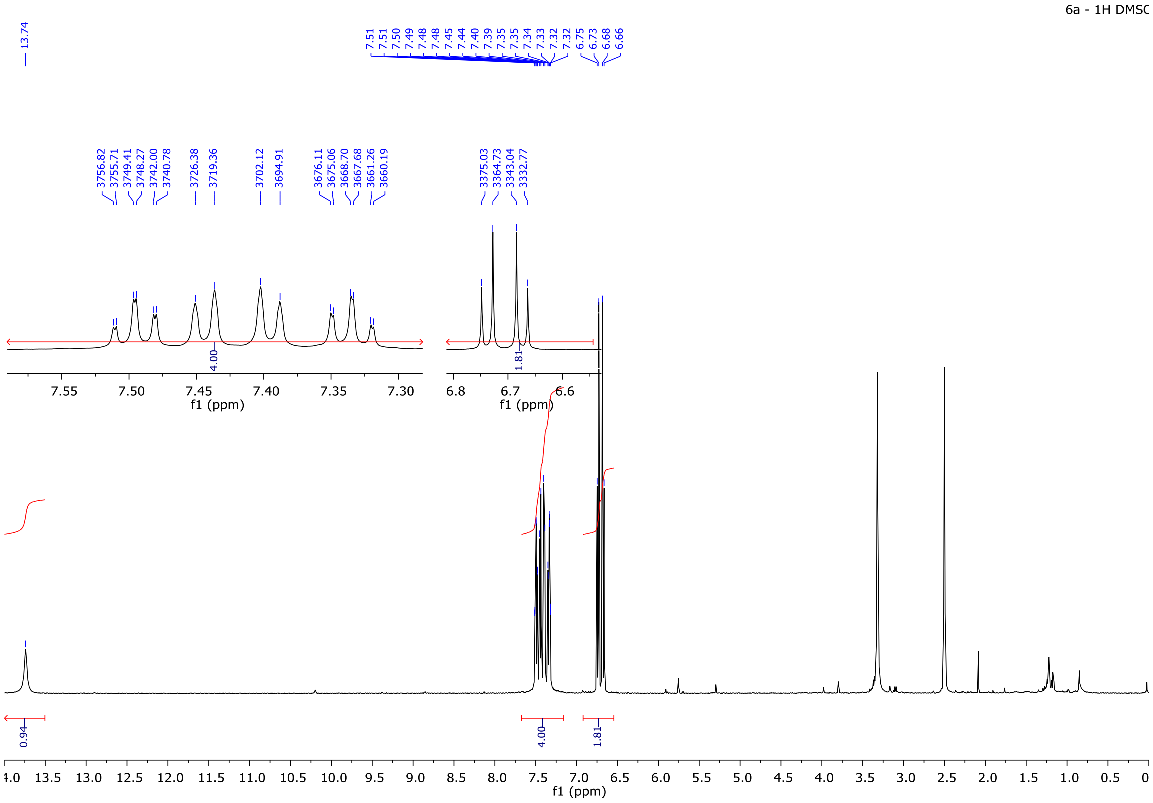

6a - 13C DMSO

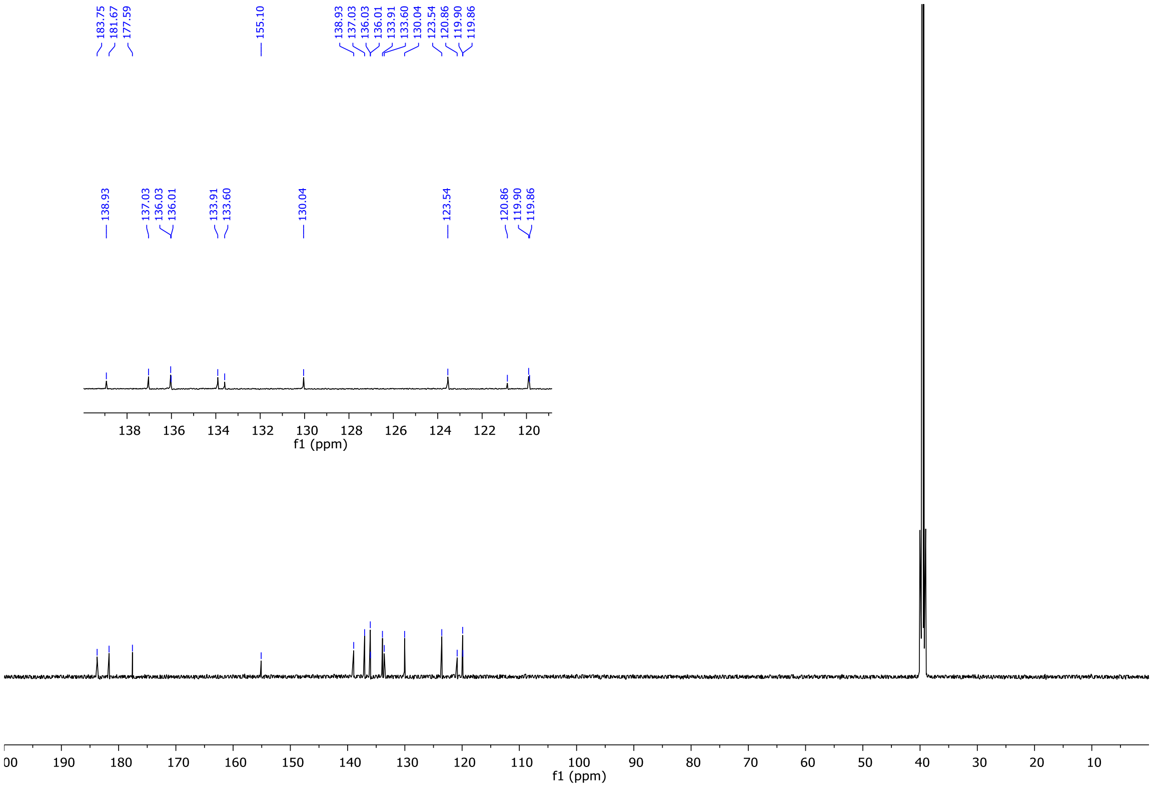

Analysis Info

Analysis Name	QTOF_120615_11_ZW18.d		
Method	MS_inf_TL_50_1000_Pos_CCSM_2.m	Acquisition Date	6/15/2012 11:51:31 AM
Comment		Instrument / Ser#	micrOTOF-Q II 10231

Acquisition Parameter

Source Type	ESI	Ion Polarity	Positive	Set Nebulizer	0.4 Bar		
Focus	Not active	Set Capillary	3000 V	Set Dry Heater	200 °C		
Scan Begin	50 m/z	Set End Plate Offset	-500 V	Set Dry Gas	4.0 l/min		
Scan End	1000 m/z	Set Collision Cell RF	50.0 Vpp	Set Divert Valve	Waste		

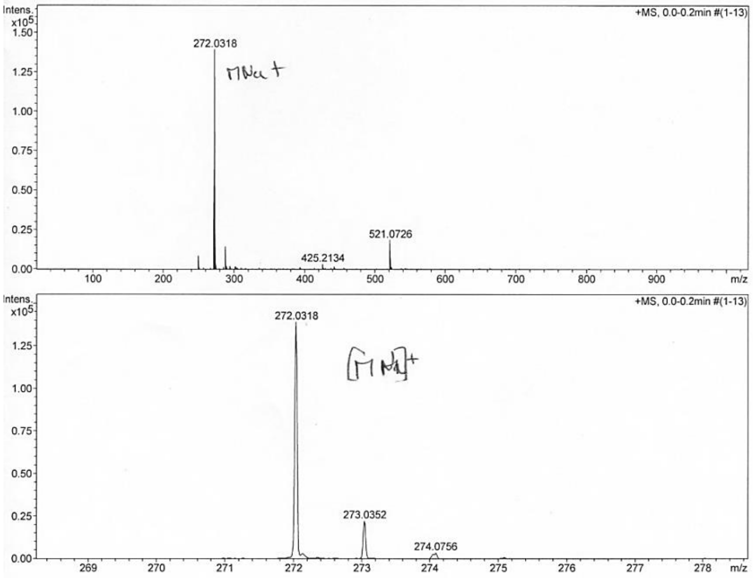

Meas. m/z	Formula	m/z	err [ppm]	mSigma
272.0318	C 15 H 7 N Na O 3	272.0318	0.1	11.3

5-Methyl-5*H*-indeno[1,2-*b*]indole-6,9,10-trione (6b)

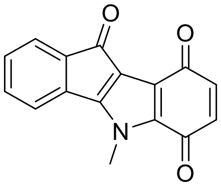

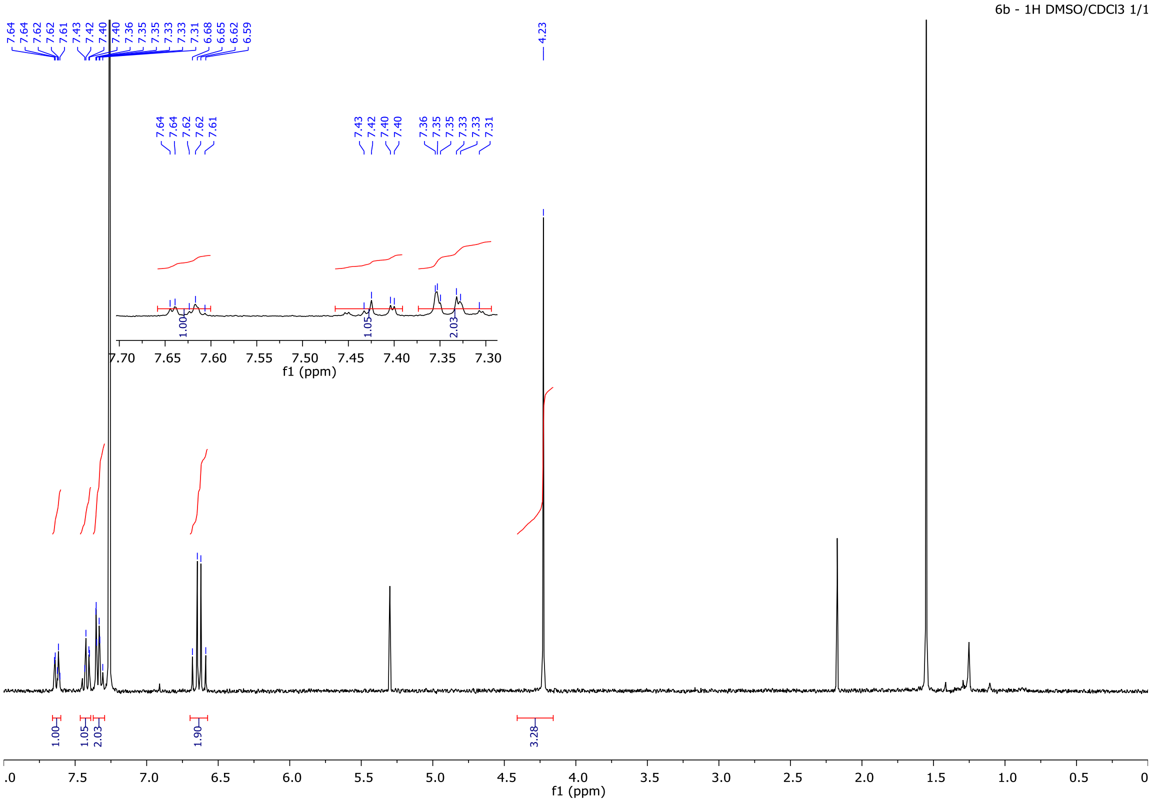

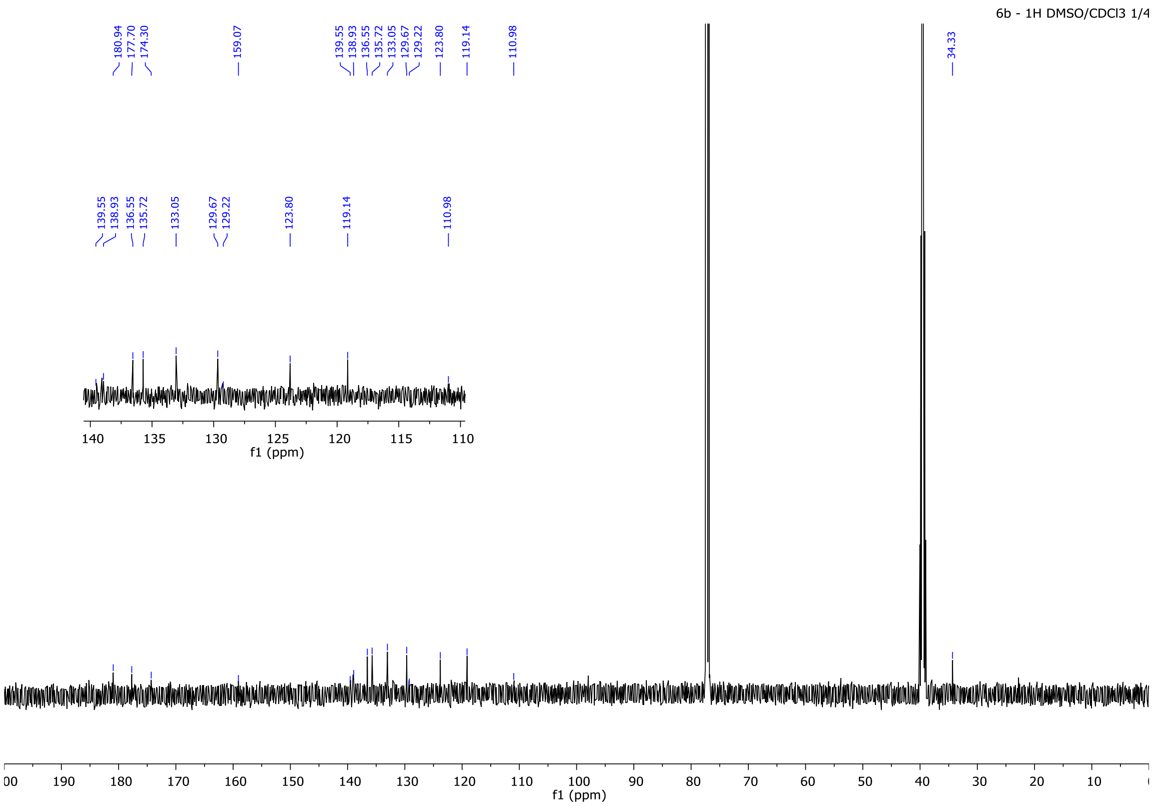

Analysis Info

Analysis Name	QTOF_120615_13_ZW15.d		
Method	MS_inf_TL_50_1000_Pos_CCSM_2.m	Acquisition Date	6/15/2012 11:58:03 AM
Comment		Instrument / Ser#	micrOTOF-Q II 10231

Acquisition Parameter

Source Type	ESI	Ion Polarity	Positive	Set Nebulizer	0.4 Bar
Focus	Not active	Set Capillary	3000 V	Set Dry Heater	200 °C
Scan Begin	50 m/z	Set End Plate Offset	-500 V	Set Dry Gas	4.0 l/min
Scan End	1000 m/z	Set Collision Cell RF	50.0 Vpp	Set Divert Valve	Waste

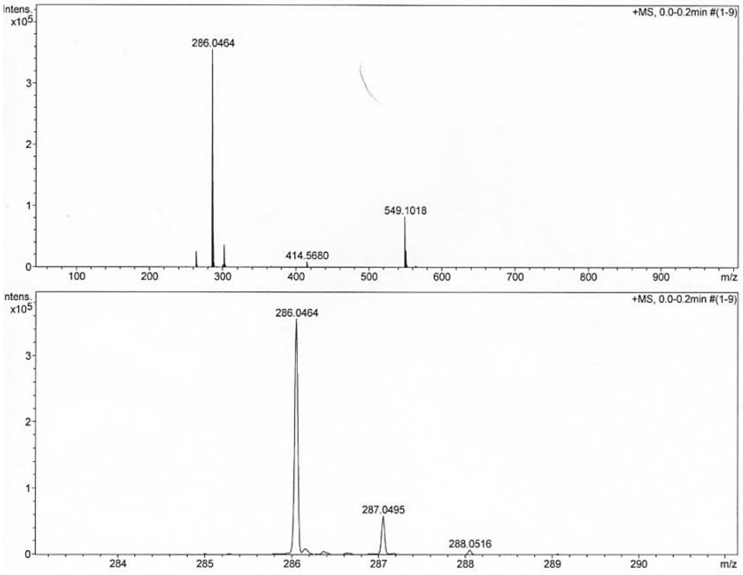

Meas. m/z	Formula	m/z	err [ppm]	mSigma
286.0464	C 16 H 9 N Na O 3	286.0475	3.6	6.7

5-Ethyl-5*H*-indeno[1,2-*b*]indole-6,9,10-trione (6c)

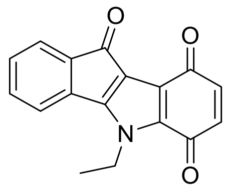

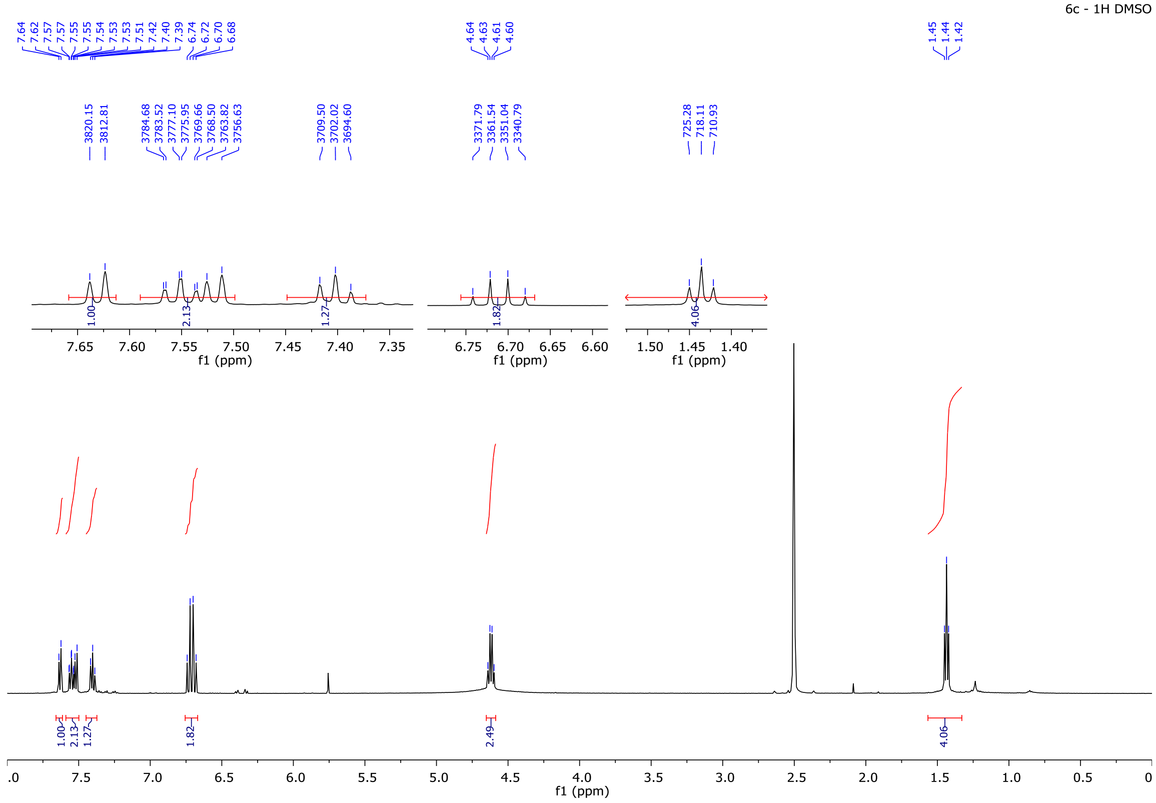

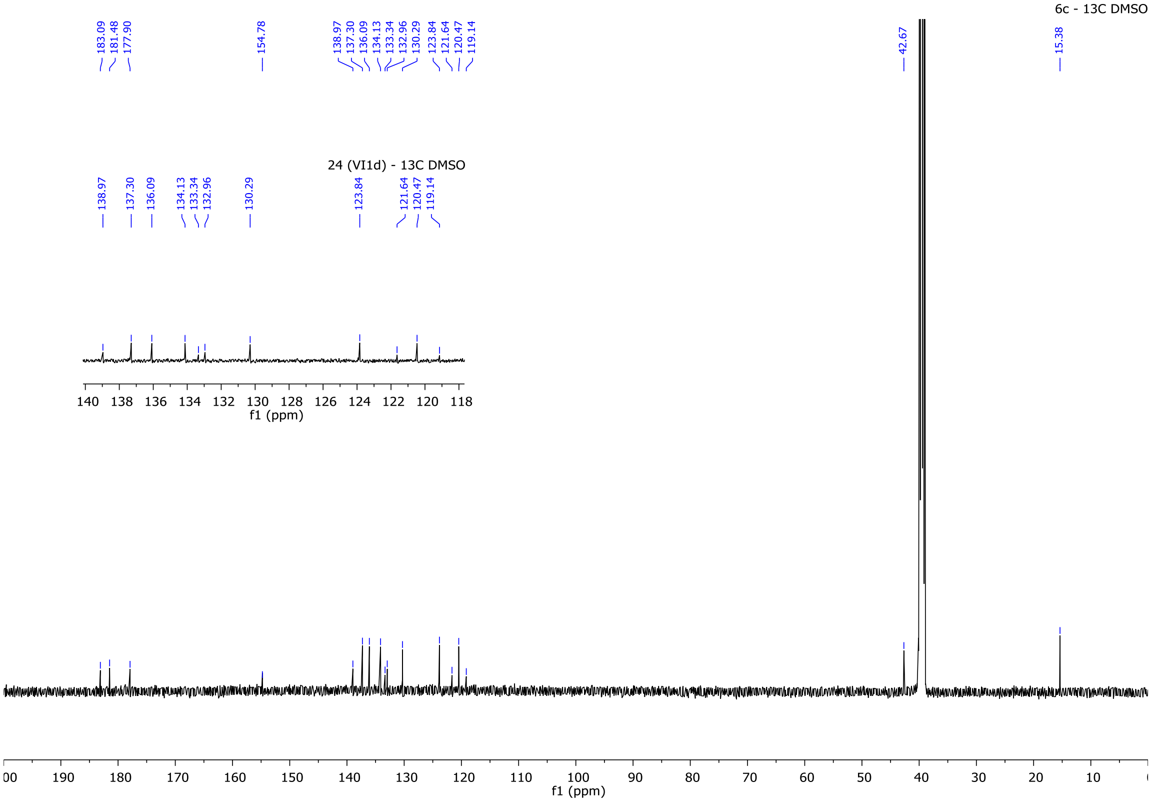

Analysis Info

Analysis Name	QTOF_120615_12_ZW16.d		
Method	MS_inf_TL_50_1000_Pos_CCSM_2.m	Acquisition Date	6/15/2012 11:54:19 AM
Comment		Instrument / Ser#	micrOTOF-Q II 10231

Acquisition Parameter

Source Type	ESI	Ion Polarity	Positive	Set Nebulizer	0.4 Bar
Focus	Not active	Set Capillary	3000 V	Set Dry Heater	200 °C
Scan Begin	50 m/z	Set End Plate Offset	-500 V	Set Dry Gas	4.0 l/min
Scan End	1000 m/z	Set Collision Cell RF	50.0 Vpp	Set Divert Valve	Waste

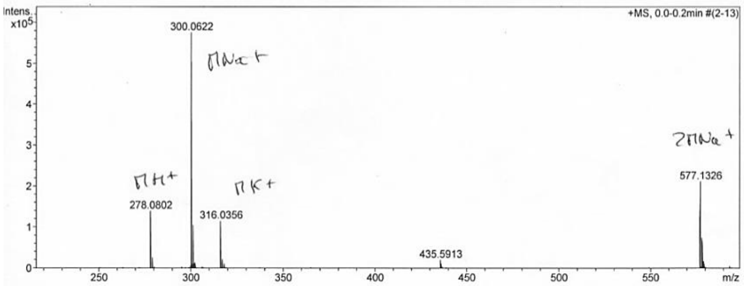

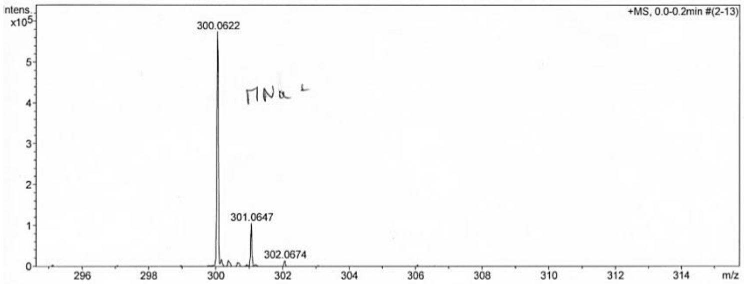

Meas. m/z	Formula	m/z	err [ppm]	mSigma
300.0622	C 17 H 11 N Na O 3	300.0631	3.0	2.3

1-Bromo-5-isopropyl-5*H*-indeno[1,2-*b*]indole-6,9,10-trione (6d)

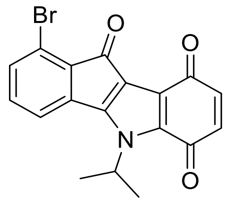

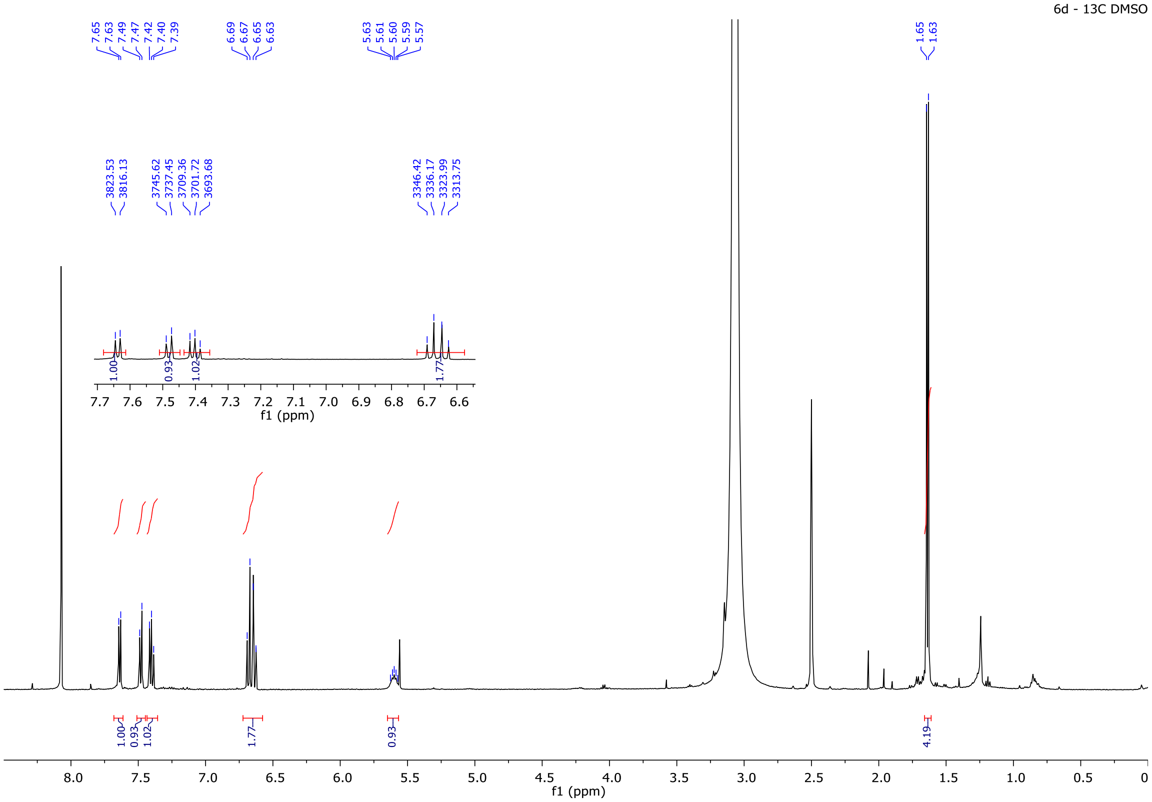

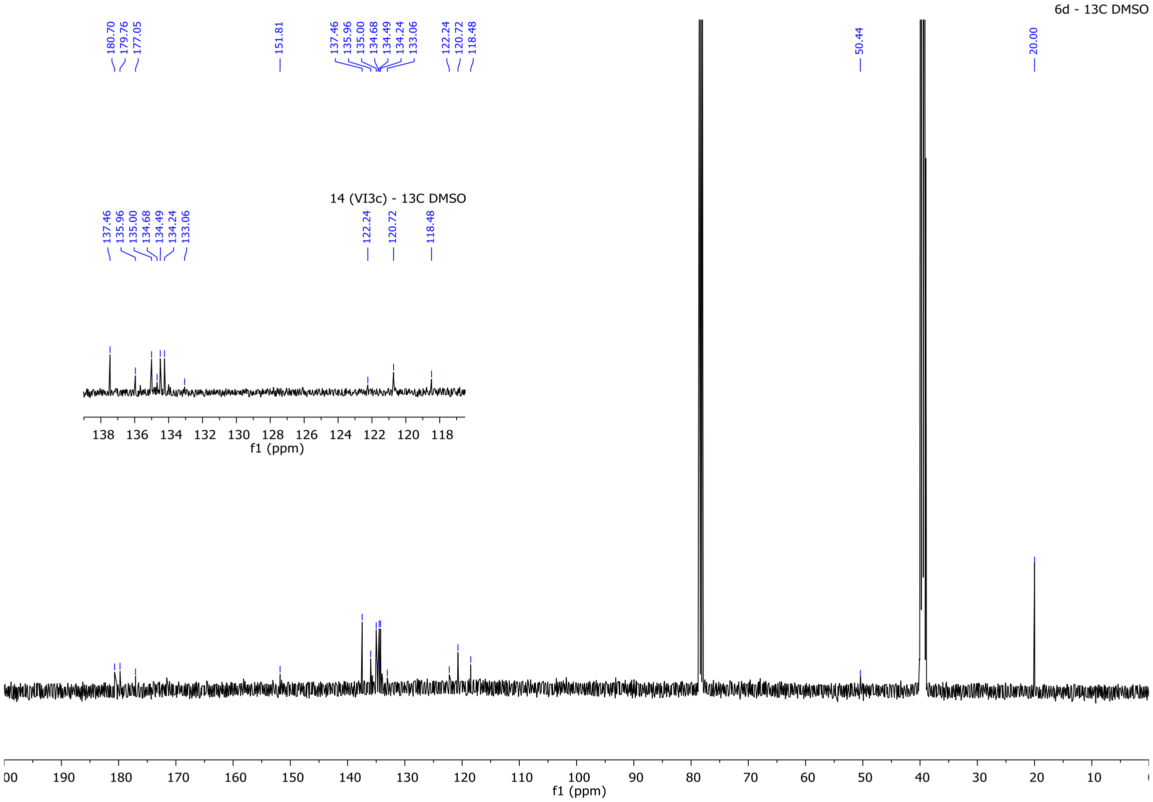

Analysis Info

Analysis Name	Impact2_190708_05_CM3131B.d		
Method	Tune_pos_Standard.m	Acquisition Date	7/8/2019 3:02:38 PM
Comment		Instrument / Ser#	impact II 1825265.10081

Acquisition Parameter

Source Type	ESI	Ion Polarity	Positive	Set Nebulizer	0.3 Bar
Focus	Active	Set Capillary	4500 V	Set Dry Heater	200 °C
Scan Begin	50 m/z	Set End Plate Offset	-500 V	Set Dry Gas	4.0 l/min
Scan End	1200 m/z	Set Collision Cell RF	750.0 Vpp	Set Divert Valve	Source

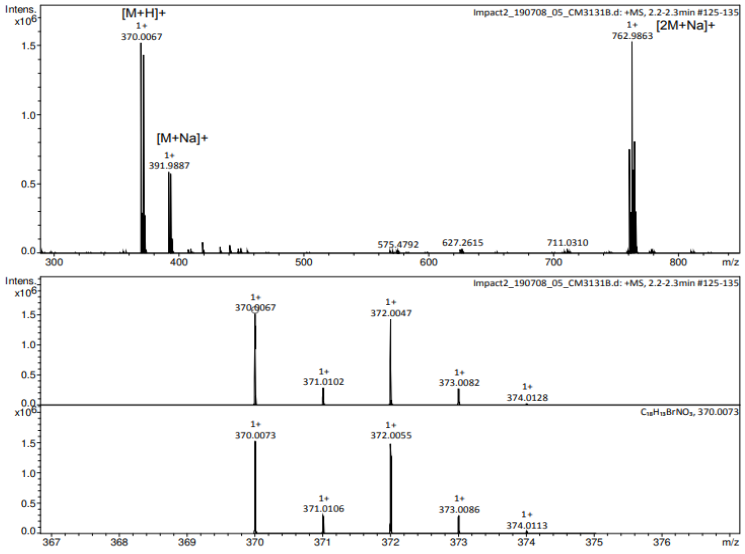

Meas. m/z	Ion Formula	m/z	err [ppm]	mSigma
370.0067	C18H13BrNO3	370.0073	1.8	26.8
391.9887	C18H12BrNNaO3	391.9893	1.4	14.4
760.9881	C36H24Br2N2NaO6	760.9893	1.7	10.3

5-Isopropyl-3-methoxy-5*H*-indeno[1,2-*b*]indole-6,9,10-trione (6e)

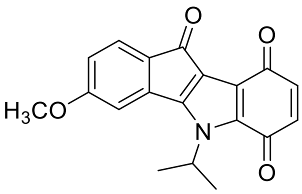

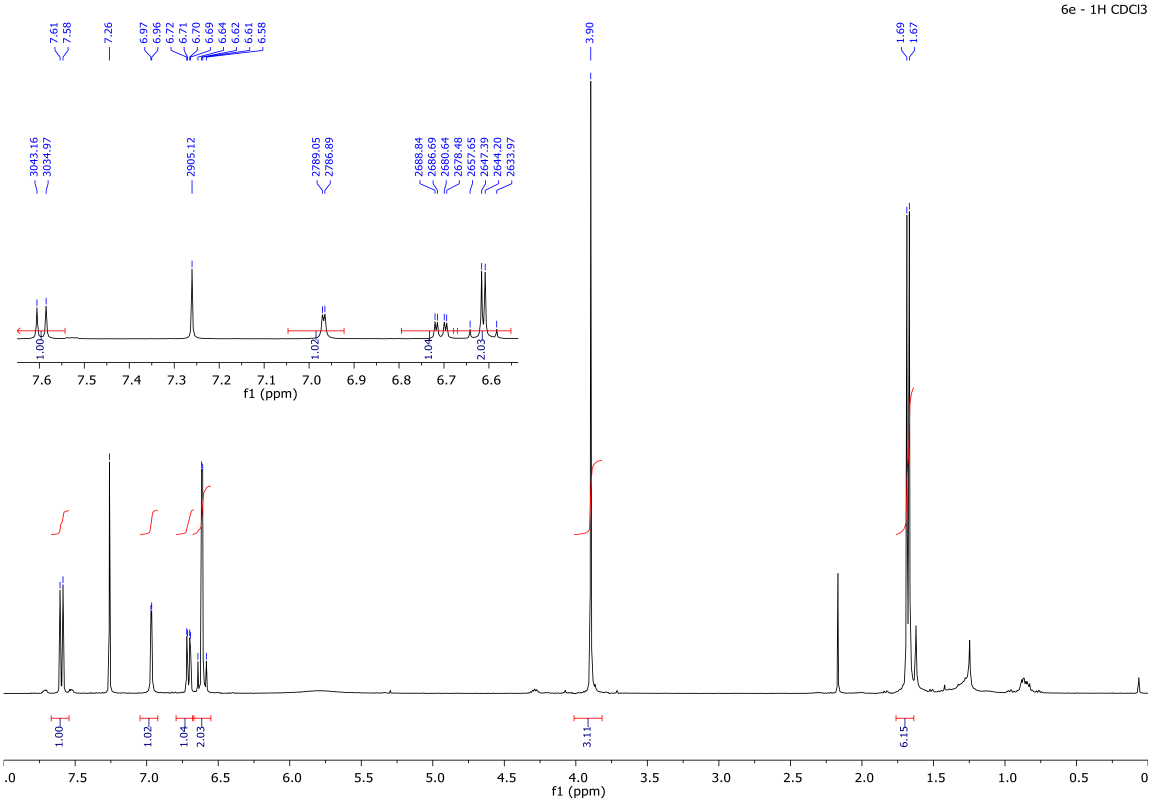

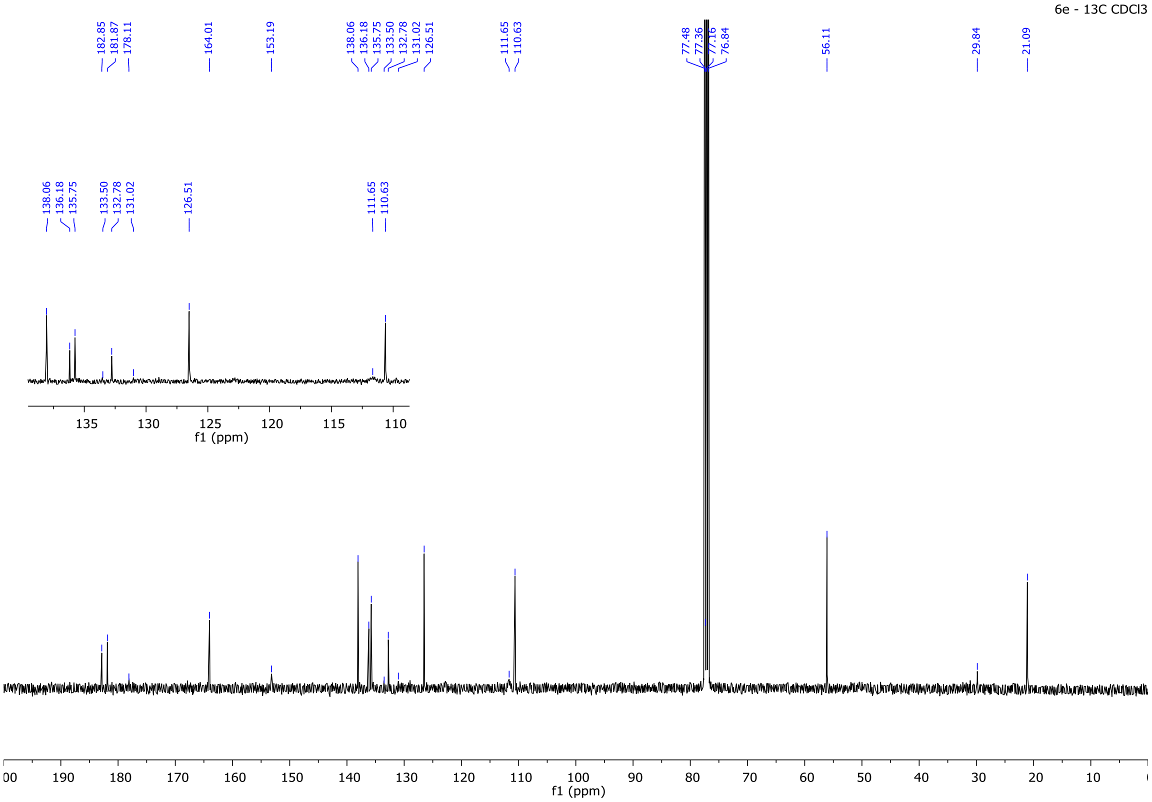
6e - 13C CDCl3
182.85
181.87
178.11
164.01
153.19
138.06
136.18
135.75
133.50
132.78
131.02
126.51
111.65
110.63
77.48
77.36
77.16
76.84
56.11
29.84
21.09
f1 (ppm)

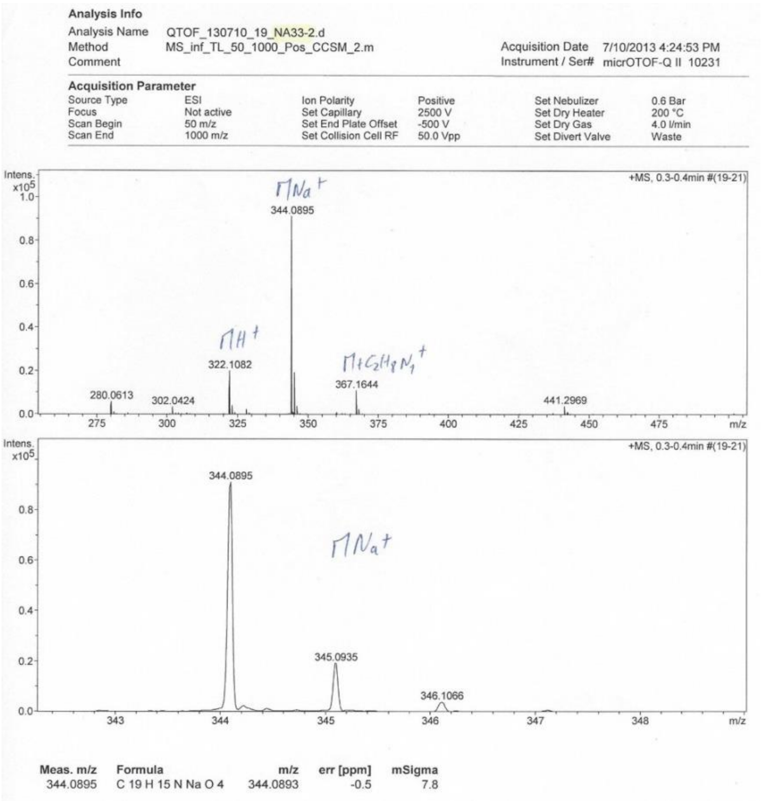
Analysis Info
Analysis Name QTOF_130710_19_NA33-2.d
Method MS_inf_TL_50_1000_Pos_CCSM_2.m
Comment
Acquisition Date 7/10/2013 4:24:53 PM
Instrument / Ser# micrOTOF-Q II 10231
Acquisition Parameter
Source Type ESI
Focus Not active
Scan Begin 50 m/z
Scan End 1000 m/z
Ion Polarity Positive
Set Capillary 2500 V
Set End Plate Offset -500 V
Set Collision Cell RF 50.0 Vpp
Set Nebulizer 0.6 Bar
Set Dry Heater 200 °C
Set Dry Gas 4.0 l/min
Set Divert Valve Waste
+MS, 0.3-0.4min #(19-21)
MNa+
344.0895
MH+
322.1082
M+C2H8N1+
367.1644
280.0613
302.0424
441.2969
345.0935
346.1066
Meas. m/z Formula m/z err [ppm] mSigma
344.0895 C 19 H 15 N Na O 4 344.0893 -0.5 7.8

5-Isopropyl-4-methoxy-5H-indeno[1,2-b]indole-6,9,10-trione (6f)

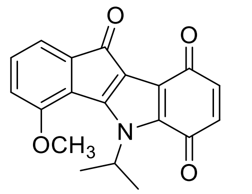

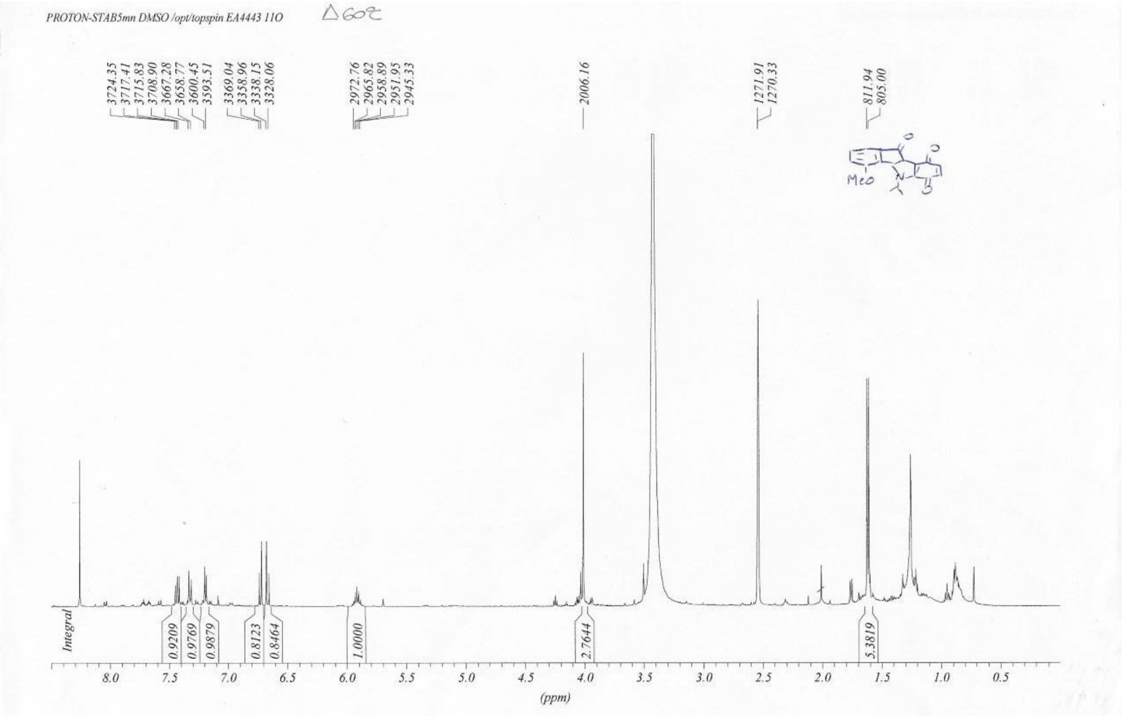

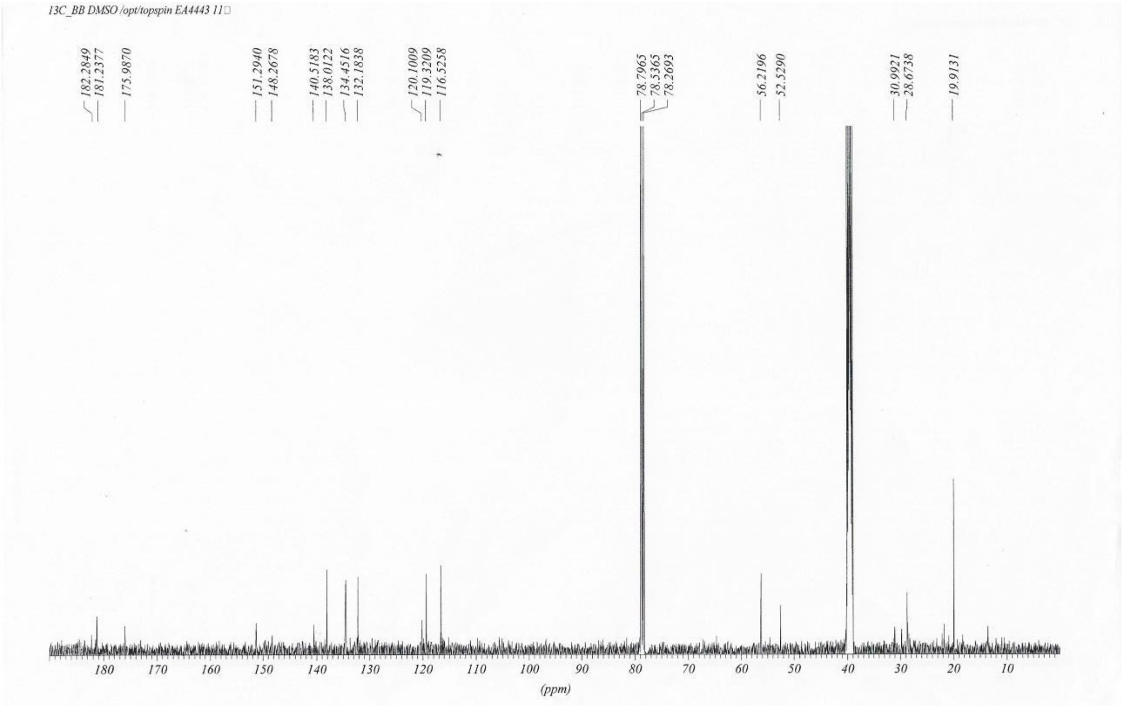

Supplement: Supplementary file 1 [file molecules-25-00097-s001.zip › molecules-635534-S2.pdf]
