# Supplementary material for: QSAR Model of Indeno[1,2-b]indole Derivatives and Identification of N-isopentyl-2-methyl-4,9-dioxo-4,9-Dihydronaphtho[2,3-b]furan-3-carboxamide as a Potent CK2 Inhibitor
Source: Molecules. 2019 Dec 26;25(1):97. doi: 10.3390/molecules25010097 (PMC6982966; doi:10.3390/molecules25010097)
Supplement: Supplementary file 1 [file molecules-25-00097-s001.zip › molecules-635534-S1.pdf]

Supplementary Materials

QSAR model of indeno[1,2b]indole derivatives and identification of *N*-isopentyl-2-methyl-4,9-dioxo-4,9-dihydronaphtho[2,3-*b*]furane-3-carboxamide as potent CK2 inhibitor

Samer Haidar[a,b,] , Christelle Marminon[c], Dagmar Aichele[a], Abdelhamid Nacereddine[c], Wael Zeinyeh[c], Abdeslem Bouzina[c,d], Malika Berredjem[d], Laurent Ettouati[c], Zouhair Bouaziz[c], Marc Le Borgne[c], Joachim Jose[a] *

[a] *Institut für Pharmazeutische und Medizinische Chemie, PharmaCampus, Westfälische Wilhelms-Universität Münster, Corrensstr. 48, 48149 Münster, Germany*

[b] *Faculty of Pharmacy, 17 April street, Damascus University, Damascus, Syria*

[c] *Université de Lyon, Université Claude Bernard Lyon 1, Faculté de Pharmacie - ISPB, EA 4446 Bioactive Molecules and Medicinal Chemistry, SFR Santé Lyon-Est CNRS UMS3453 - INSERM US7, 8 Avenue Rockefeller, F-69373, Lyon Cedex 8, France*

[d] *Laboratory of Applied Organic Chemistry, Synthesis of Biomolecules and Molecular Modelling Group, Badji-Mokhtar—Annaba University, Box 12, 23000 Annaba, Algeria*

* Corresponding author contact: Tel. 0049 251-8332200, email: joachim.jose@uni-muenster.de

Table of contents

Structures of indenoindoles used in the study S3

Synthetic pathways S4

General considerations S6

General procedure for the synthesis of dihydroxylylated derivatives **3** S7

General procedures for the synthesis of ketone derivatives **4** S9

General procedures for the synthesis of phenol derivatives **5** S14

General procedure for the synthesis of *para*-quinone derivatives **6** S17

References S19

Table S1. Structures of indenoindoles used in the study.

Cpd No.	Chemical structure	Cpd No.	Chemical structure	Cpd No.	Chemical structure
**4d**	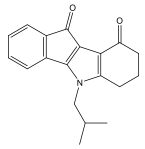	**4e**	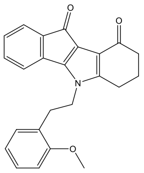	**4f**	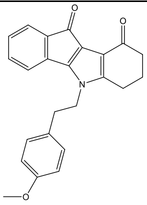
**4g**	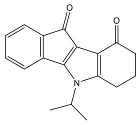	**4h**	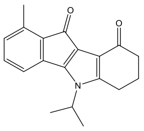	**4i**	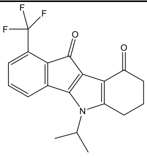
**4j**	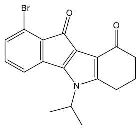	**4p**	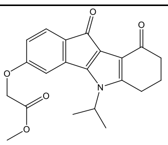	**4q**	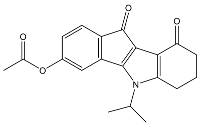
**4r**	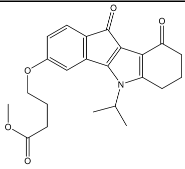	**4s**	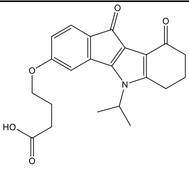	**4v**	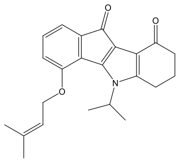
**4w**	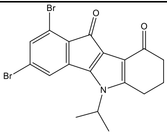	**4x**	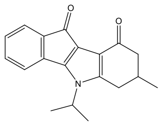	**4y**	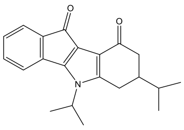
**5a**	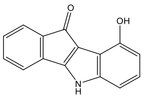	**5c**	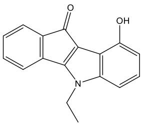	**5d**	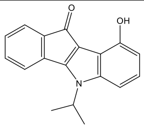
**5f**	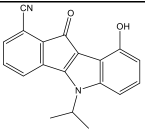	**5g**	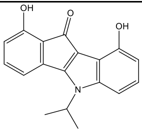	**5h**	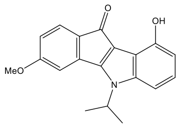
**5j**	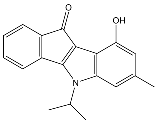	**5k**	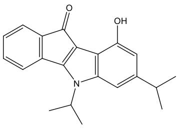	**6a**	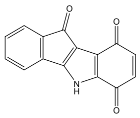
**6b**	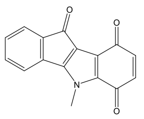	**6c**	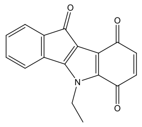	**6d**	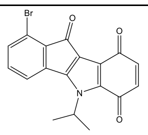
**6e**	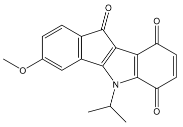	**6f**	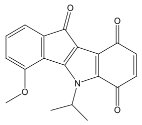	**6g**	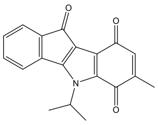

Synthetic pathways

Synthetic pathways of all compounds are gathered together in Scheme S1. New compounds and their intermediates are described herein.

5,6,7,8-Tetrahydroindeno[1,2-*b*]indole-9,10-diones **4** are usually prepared in two steps from the corresponding ninhydrin **1** and enaminone **2**, as previously described, [1, 2] *i.e.* condensation in methanol then dedihydroxylation with tetraethylthionylamide (TETA) in a mixture of acetic acid and DMF.

Derivatives **4b** and **4c** were prepared by *N*-alkylation of 5,6,7,8-tetrahydroindeno[1,2-*b*]indole-9,10-dione **4a** [2] in ethanol using potassium hydroxide as the base and alkyl iodide as the alkylating agent. Reflux two days was enough for the methyl derivative **4b**, but not for the ethyl one **4c**, which was heated two hours to 120 ℃ under microwave irradiation. Carbonitrile **4k** was obtained by microwave-assisted cyanation of bromide **4j** according to the described procedure [3]. *O*-alkylations of the 3-hydroxy derivative **4n** were performed with potassium carbonate in acetone at reflux. Methyl ester **4r** was hydrolysed with an aqueous potassium hydroxide in methanol into carboxylic acid **4s** [4].

Compounds **4** were aromatized with Pd-C in diphenylether at 250 ℃ for 3-6 h.[5] To avoid debromination, **5e** was oxidized with DDQ in dioxane [6] under microwave irradiation at 140 ℃ for 12 min. Finally, oxidations of phenols **5** to *para*-quinones **6** were achieved using Fremy's salt [7].

The procedures were already described for compounds **3a**, **3e**, **4a**, **4g**,[2] **3c**, **3d**, **3i**, **3l**, **4e**, **4f**, **4l**, **4t**, **4v** [1], **3j**, **3m**, **4u** [8], **3o**, **3p**, **4x**, **4y**, **5j**, **5k**, **6g** [9] , **4g** and **5d** [6].

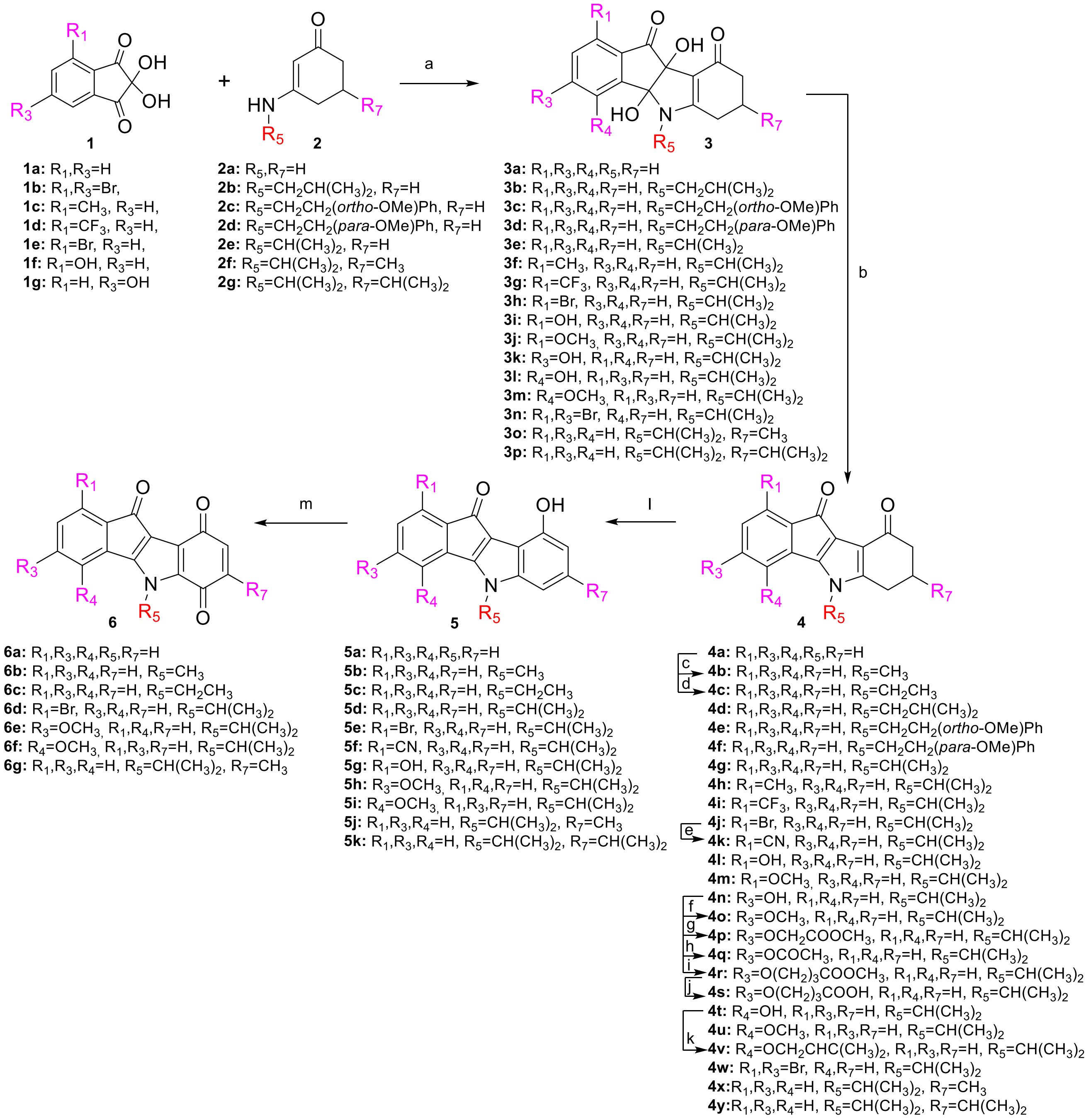

Conditions: (a) MeOH, rt; (b) $(NEt_2)_2SO$ (TETA), DMF, AcOH, rt; (c) KOH, EtOH, CH_3I, 70°C; (d) KOH, EtOH, CH_3CH_2I, 120°C m.w.; (e) CuCN, NMP, m.w.; (f) Me_2SO_4, K_2CO_3, acetone, reflux; (g) $ClCH_2COOCH_3$, K_2CO_3, acetone, refux; (h) $ClCOOCH_3$, K_2CO_3, acetone, reflux; (i) $Cl(CH_2)_3COOCH_3$, K_2CO_3, acetone, reflux; (j) NaOH, MeOH, rt; (k) $BrCH_2CHC(CH_3)_2$, K_2CO_3, acetone, refux; (l) 10% Pd-C, Ph_2O, reflux or DDQ, dioxane, mw 12 min., 140°C ; (m) Fremy's salt, KH_2PO_4, acetone/water or salcomine, DMF, O_2, rt.

Scheme S1. Synthesis of key intermediates and targeted compounds.

General considerations

All of the reagents were purchased from Sigma-Aldrich and ThermoFisher Scientific. Melting points were determined on a Electrothermal 9200 capillary apparatus. The IR spectra were recorded on a Perkin Elmer Spectrum Two IR Spectrometer. The ^{1}H and ^{13}C NMR spectra were recorded at 400 MHz on a Brücker DRX 400 spectrometer. Chemical shifts are expressed in ppm (δ) downfield from internal tetramethylsilane and coupling constants *J* are reported in hertz (Hz). The following abbreviations are used: s: singlet; d: doublet; t: triplet; dd: doubled doublet; q: quartet; qui:quintuplet; sept:septuplet; m: multiplet; Cquat: quaternary carbons. The mass spectra were performed by direct ionization (EI or CI) on a ThermoFinnigan MAT 95 XL apparatus. Elemental analysis was performed at the Centre de Microanalyse, CNRS, Solaize, France. Microwave experiments were carried out using BIOTAGE INITIATOR Microwave synthesizer 2.0 440W. Chromatographic separations were performed on silica gel columns by column chromatography (Kieselgel 300–400 mesh). All reactions were monitored by TLC on GF254 plates that were visualized under a UV lamp (254 nm). Evaporation of solvent was performed *in vacuo* with rotating evaporator. The purity of the final compounds (greater than 95%) was determined by uHPLC/MS on an Agilent 1290 system using a Agilent 1290 Infinity ZORBAX Eclipse Plus C18 column (2.1 x 50 mm, 1.8 µm particle size) with a gradient mobile phase of H_2O/CH_3CN (90:10, v/v) with 0.1% of formic acid to H_2O/CH_3CN (10:90, v/v) with 0.1% of formic acid at a flow rate of 0.5 mL/min, with UV monitoring at the wavelength of 254 nm with a runtime of 10 min.

General procedure for the synthesis of dihydroxylylated derivatives **3**

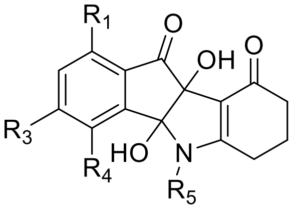

Figure S1. Structure of dihydroxylylated derivatives **3**

General procedure for the synthesis of 4b,9b-dihydroxy-4b,5,6,7,8,9b-hexahydroindeno[1,2-*b*]indole-9,10-diones

A solution of enaminone **2** (6.54 mmol) and ninhydrin **1** (6.54 mmol) in methanol (10 mL) was stirred at room temperature for 22 h. A precipitate of compound **3** was formed. It was recovered and washed with methanol. A second quantity was obtained from the filtrate by purification by silica gel column chromatography with CH_2Cl_2/acetone (1:2, v/v) as eluent.

4b,9b-Dihydroxy-5-isobutyl-4b,5,6,7,8,9b-hexahydroindeno[1,2-*b*]indole-9,10-dione (3b): yellow solid, 88% yield, mp 172 °C. IR *ν* cm^{-1}): 3400 (OH), 1733 (C=O), 1715 (C=O). ^{1}H NMR ($CDCl_3$, 400 MHz): δ 7.83-7.79 (m, 2H, Harom), 7.71 (m, 1H, Harom), 7.51 (m, 1H, Harom), 5.35 (bs, 1H, OH), 5.01 (bs, 1H, OH), 3.59 (m, 1H), 3.26 (m, 1H), 2.47 (m, 1H), 2.35-2.20 (m, 4H), 1.97 (m, 1H), 1.75 (m, 1H), 0.97 (m, 6H, 2CH_3). ^{13}C NMR + DEPT ($CDCl_3$, 100 MHz): δ 197.9 (C=O), 192.4 (C=O), 167.2 (Cquat), 148.0 (Cquat), 135.9 (CH), 135.4 (Cquat), 130.6 (CH), 125.0 (CH), 124.5 (CH), 105.5 (Cquat), 95.9 (Cquat), 83.1 (Cquat), 50.6 (CH_2), 36.5 (CH_2), 29.2 (CH), 24.0 (CH_2), 21.8 (CH_2), 20.7 (CH_3), 20.6 (CH_3). HRMS calcd for $C_{19}H_{22}NO_4$ $[M+H]^+$ 328.1543, found 328.1537.

4b,9b-Dihydroxy-5-isopropyl-1-methyl-4b,5,6,7,8,9b-hexahydroindeno[1,2-*b*]indole-9,10-dione (3f): Pale beige solid, 74% yield, mp 225 °C. IR *ν* cm^{-1}): 3000-2600 (OH), 1713 (C=O). ^{1}H NMR (DMSO-d_6, 400 MHz): δ 7.77 (d, 1H, *J* = 7.7 Hz, H-2), 7.67 (dd, 1H, *J* = 7.7 Hz *J* = 7.5 Hz, H-3), 7.36 (d, 1H, *J* = 7.5 Hz, H-4), 6.67 (s, 1H, OH), 5.59 (bs, 1H, OH), 4.57 (sept, 1H, *J* = 7.1 Hz, C<u>H</u>(CH_3)$_2$), 2.74-2.67 (m, 1H, H-6), 2.57 (s, 3H, CH_3), 2.52-2.47 (m, 1H, H-6), 2.09 (t, 2H, *J* = 6.3 Hz, H-8), 1.88-1.77 (m, 2H, H-7), 1.46 (d, 3H, *J* = 6.9 Hz, CH_3), 1.25 (d, 3H, *J* = 7.2 Hz, CH_3). ^{13}C NMR + DEPT (DMSO-d_6**,** 100 MHz): δ 198.8 (Cquat), 188.8 (Cquat), 164.5 (Cquat), 149.0 (Cquat), 137.4 (Cquat), 134.5 (CH), 132.1 (Cquat), 131.6 (CH), 121.8 (CH), 105.4 (Cquat), 95.0 (Cquat), 82.6 (Cquat), 44.8 (CH), 36.7 (CH_2), 24.2 (CH_2), 22.6 (CH_3), 22.2 (CH_3), 22.0 (CH_2), 18.0 (CH_3). HRMS calcd for $C_{19}H_{22}NO_4$ $[M+H]^+$ 328.1543, found 328.1540.

4b,9b-Dihydroxy-5-isopropyl-1-(trifluoromethyl)-4b,5,6,7,8,9b-hexahydroindeno[1,2-*b*]indole-9,10-dione (3g): Pale beige solid, 85% yield, mp 299 °C. IR *ν* cm^{-1}): 3331 (OH), 1738 (C=O). ^{1}H NMR (DMSO-d_6, 400 MHz): δ 8.25 (dd, 1H, *J* = 7.5 Hz, *J* = 1.4 Hz, H-2), 7.97-7.90 (m, 2H, H-3 and H-4), 6.94 (s, 1H, OH), 5.79 (s, 1H, OH), 4.60 (sept, 1H, *J* = 7.1 Hz, C<u>H</u>(CH_3)$_2$), 2.74-2.67 (m, 1H, H-6), 2.48-2.44 (m, 1H, H-6), 2.05 (t, 2H, *J* = 6.4 Hz, H-8), 1.84-1.74 (m, 2H, H-7), 1.43 (d, 3H, *J* = 6.9 Hz, CH_3), 1.25 (d, 3H, *J* = 7.2 Hz, CH_3). ^{13}C NMR + DEPT (DMSO-d_6, 100 MHz): δ 194.50 (Cquat), 188.72 (Cquat), 164.92 (Cquat), 149.65 (Cquat), 135.32 (CH), 131.56 (Cquat), 129.03

(CH), 127.90 ($^3J_{CF}$ = 5.9Hz, CH-2), 124.75 ($^2J_{CF}$ = 34 Hz, C-1), 122.83 ($^1J_{CF}$ = 273 Hz, CF_3), 105.00 (Cquat), 94.66 (Cquat), 82.78 (Cquat), 44.87 (CH), 36.74 (CH_2), 24.26 (CH_2), 22.62 (CH_3), 22.43 (CH_3), 21.98 (CH_2). HRMS calcd for $C_{19}H_{18}F_3NNaO_4$ $[M+Na]^+$ 404.1080 found 404.1081.

1-Bromo-4b,9b-dihydroxy-5-isopropyl-4b,5,6,7,8,9b-hexahydroindeno[1,2-*b*]indole-9,10-dione (3h): beige solid, 80% yield, mp 207 ℃. IR *ν* cm^{-1}): 3119 (OH), 1726 (C=O), 696 (C-Br). ^1H NMR (400 MHz, DMSO-d_6): δ 7.99 (d, 1H, *J* = 7.8 Hz, H-2), 7.80 (d, 1H, *J* = 7.6 Hz, H-4), 7.71 (t, 1H, *J* = 7.8 Hz, H-3), 6.89 (s, 1H, OH), 5.79 (se, 1H, OH), 4.59 (sept, 1H, *J* = 7.1 Hz, C<u>H</u>(CH_3)$_2$), 2.77-2.70 (m, 1H, H-8), 2.52-2.49 (m, 1H, H-8), 2.10 (t, 2H, *J* = 6.3 Hz, H-6), 1.87-1.79 (m, 2H, H-7), 1.46 (d, 3H, *J* = 7.1 Hz, CH_3), 1.27 (d, 3H, *J* = 7.3 Hz, CH_3). ^{13}C NMR (100 MHz, DMSO-d_6): δ 195.12 (C=O), 188.77 (C=O), 164.87 (Cquat), 150.84 (Cquat), 136.26 (CH), 134.81 (CH), 131.95 (Cquat), 123.87 (CH), 118.27 (Cquat), 104.93 (Cquat), 94.32 (Cquat), 83.09 (Cquat), 44.84 (CH), 36.73 (CH_2), 24.23 (CH_2), 22.58 (CH_3), 22.25 (CH_3), 21.94 (CH_2). HRMS calcd for $C_{18}H_{19}BrNO_4$ $[M+H]^+$ 392.0492, found: 392.0479.

3,4b,9b-Trihydroxy-5-isopropyl-4b,5,6,7,8,9b-hexahydroindeno[1,2-*b*]indole-9,10-dione (3k): grey solid, 88% yield, mp 236 ℃. IR *ν* cm^{-1}): 3070 (O-H), 1705 (C=O), 1600 (C=O). ^1H NMR (400 MHz, DMSO): δ 10.75 (s, 1H, OH), 7.57 (d, 1H, *J* = 8.4 Hz, H-1), 7.17 (d, 1H, *J* = 2.1 Hz, H-4), 6.99 (dd, 1H, *J* = 8.4 Hz, *J* = 2.1 Hz, H-2), 6.65 (s, 1H, OH), 5.57 (s, 1H, OH), 4.45 (m, 1H, NC<u>H</u>(CH_3)$_2$), 2.71-2.66 (m, 1H, CH_2), 2.54-2.50 (m, 1H, CH_2), 2.08 (t, 2H, *J* = 6.2 Hz, CH_2), 1.89-1.74 (m, 2H, CH_2), 1.46 (d, 3H, *J* = 6.8 Hz, CH_3), 1.27 (d, 3H, *J* = 7.1 Hz, CH_3). ^{13}C NMR + DEPT (100 MHz, DMSO): δ 196.86 (C=O), 189.85 (C=O), 165.41 (Cquat), 165.15 (Cquat), 152.22 (Cquat), 127.54 (Cquat), 126.39 (CH), 119.58 (CH), 110.42 (Cquat), 106.70 (CH), 96.26 (Cquat), 84.11 (Cquat), 45.81 (CH), 37.65 (CH_2), 25.22 (CH_2), 23.55 (CH_3), 23.04 (CH_3), 23.00 (CH_2). HRMS calcd for $C_{18}H_{19}NNaO_5$ $[M+Na]^+$ 352.1155, found 352.1151.

1,3-Dibromo-4b,9b-dihydroxy-5-isopropyl-4b,5,6,7,8,9b-hexahydroindeno[1,2-*b*]indole-9,10-dione (3n): yellow solid, 78% yield, mp 197 ℃. IR *ν* cm^{-1}): 3320 (OH), 1724 (C=O), 692 (C-Br). ^1H NMR (400 MHz, $CDCl_3$): δ 7.71 (d, 1H, *J* = 1.3 Hz, H-2), 7.62 (d, 1H, *J* = 1.3 Hz, H-4), 7.26 (bs, 1H, OH), 6.07 (bs, 1H, OH), 4.30 (sept, 1H, *J* = 6.9 Hz, C<u>H</u>(CH_3)$_2$), 2.23-2.46 (m, 2H, CH_2), 2.02-2.06 (m, 2H, CH_2), 1.67-1.82 (m, 2H, CH_2), 1.29 (d, 3H, *J* = 7.1 Hz, CH_3), 1.13 (d, 3H, *J* = 7.1 Hz, CH_3). ^{13}C NMR + DEPT (100 MHz, $CDCl_3$): δ 193.91 (C=O), 190.47 (C=O), 165.00 (Cquat), 151.61 (Cquat), 131.02 (Cquat), 136.97 (CH), 129.16 (Cquat), 125.84 (CH), 120.31 (Cquat), 105.29 (Cquat), 93.67 (C-OH), 82.65 (C-OH), 45.17 (CH), 35.9 (CH_2), 24.39 (CH_2), 22.50 (CH_3), 22.04 (CH_3), 21.48 (CH_2). HRMS calcd for $C_{18}H_{18}Br_2NO_4$ $[M+H]^+$ 469.9597, found: 469.9595.

General procedures for the synthesis of ketone derivatives **4**

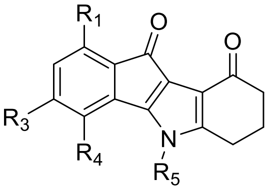

Figure S2. Structure of ketone derivatives **4**.

General procedures for the synthesis of 5,6,7,8-tetrahydroindeno[1,2-*b*]indole-9,10-diones

Method A: A solution of dihydroxylated compound **3** (1.6 mmol) and tetraethylthionylamide (TETA) (3.2 mmol) in 5 mL of DMF and 1 mL of AcOH was stirred at room temperature for 22 h. The solution was then poured into 160 mL of ice and water and stirred for 1 h. The resulting precipitate was filtered, washed with water and dried to give a first quantity of **4**. The filtrate was then concentrated in vacuum and the residue diluted with H_2O. The solution was basified with $NaHCO_3$. The organic layer was extracted with CH_2Cl_2, dried over sodium sulfate and concentrated in vacuum to give a second quantity of **4** which was purified by silica gel column chromatography with CH_2Cl_2/acetone (1:3, v/v) as eluent.

Method B: A mixture of ketone **4a** [2] (0.5 g, 2.11 mmol), potassium hydroxide (0.193 g, 3.45 mmol) and methyl iodide (0.217 mL, 3.45 mmol) in ethanol (25 mL) was heated to 70 °C for two days. Then it was poured into cooled water (150 mL). The resulting precipitate was filtered off, washed with cold water and dried to give the title compound **4b**.

Method C: To a mixture of ketone **4a** [2] (0.5 g, 2.11 mmol) and potassium hydroxide (0.189 g, 3.37 mmol) in ethanol (20 mL), in a sealed vessel, was added ethyl iodide (0.271 mL, 3.37 mmol). The mixture was heated to 120 °C for two hours under microwave irradiation and then it was poured into cooled water (150 mL). The resulting precipitate was filtered off, washed with cold water and dried to give the title compound **4c**.

Method D: A mixture of hydroxyketone **4n** (0.68 mmol, 1eq.), alkylbromide or dimethylsulfate (2.03 mmol, 3 eq.) and K_2CO_3 (2.03 mmol, 3 eq.) in 20 mL of acetone was refluxed for 6 h. The solvent was then evaporated and the residue purified by column chromatography with CH_2Cl_2/acetone (5:1, v/v) as eluent.

Method E: To a solution of ester **4q** (100 mg, 0.253 mmol) in methanol (2 mL) was added a solution of NaOH (12.1 mg, 0.303 mmol) in water (1.01 mL). The mixture was stirred at room temperature for 4 h. Then, the mixture was neutralized with acetic acid and the organic compound extracted with ethyl acetate. The organic layer was dried over Na_2SO_4 and the solvent evaporated to dryness to obtain the title compound.

Method F: A mixture of aryl bromide **4j** (100 mg, 0.28 mmol), CuCN (33 mg, 0.36 mmol), and 0.5 mL of *N*-methyl-2-pyrrolidone (NMP) was subjected to microwave irradiation (200 W) under 1 bar pressure for 20 min. After irradiation, the mixture was cooled to room temperature, diluted with 20 mL of ice water and extracted with ethyl

acetate. The organic layer was dried over sodium sulfate, concentrated and the residue purified by silica gel column chromatography using ethyl acetate/dichloromethane (25:1, v/v) as the eluent.

5-Methyl-5,6,7,8-tetrahydroindeno[1,2-*b*]indole-9,10-dione (4b): prepared according to method B. Orange solid, 96% yield, mp 250 ℃. IR (cm^{-1}): 1697 (C=O), 1659 (C=O). ^{1}H NMR (DMSO-*d*$_6$, 300 MHz): δ 7.38-7.30 (m, 2H, Harom), 7.28 (dt, 1H, *J* = 7.1 Hz, *J* = 0.9 Hz, Harom), 7.16 (td, 1H, *J* = 7.1 Hz, *J* = 1.7 Hz, Harom), 3.74 (s, 3H, CH_3), 2.78 (t, 2H, *J* = 6.3 Hz, CH_2), 2.37-2.31 (m, 2H, CH_2), 2.04 (qui, 2H, *J* = 6.3 Hz, CH_2). ^{13}C NMR + DEPT (DMSO-*d*$_6$, 100 MHz): δ 191.30 (C=O), 183.49 (C=O), 153.02 (Cquat), 151.86 (Cquat), 138.08 (Cquat), 134.56 (Cquat), 132.83 (CH), 128.42 (CH), 122.96 (CH), 118.13 (CH), 118.11 (Cquat), 116.62 (Cquat), 38.89 (CH_2), 32.51 (CH_3), 22.44 (CH_2), 21.09 (CH_2). HRMS calcd for $C_{16}H_{14}NO_2$ $[M+H]^+$ 252.1019, found 252.1012.

5-Ethyl-5,6,7,8-tetrahydroindeno[1,2-*b*]indole-9,10-dione (4c): prepared according to method C. Orange solid, 85% yield, mp 260 ℃. IR (cm^{-1}): 1692 (C=O), 1657 (C=O). ^{1}H NMR (DMSO-*d*$_6$, 300 MHz): δ 7.37-7.27 (m, 3H, Harom), 7.18 (m, 1H, *J* = 7.3 Hz, Harom), 4.17 (q, 2H, *J* = 7.2 Hz, NCH_2), 2.83 (t, 2H, *J* = 6.1 Hz, CH_2), 2.35 (t, 2H, *J* = 6.3 Hz, CH_2), 2.05 (qui, 2H, *J* = 6.3 Hz, CH_2), 1.35 (t, 3H, *J* = 7.2 Hz, CH_3). ^{13}C NMR + DEPT (DMSO-*d*$_6$, 100 MHz): δ 191.33 (C=O), 183.46 (C=O), 152.15 (Cquat), 151.06 (Cquat), 138.05 (Cquat), 134.31 (Cquat), 133.00 (CH), 128.46 (CH), 123.03 (CH), 118.53 (Cquat), 118.02 (CH), 116.77 (Cquat), 40.51 (CH_2), 37.69 (CH_2), 22.56 (CH_2), 20.93 (CH_2), 15.71 (CH_3). HRMS calcd for $C_{17}H_{16}NO_2$ $[M+H]^+$ 266.1176, found 266.1167.

5-Isobutyl-5,6,7,8-tetrahydroindeno[1,2-*b*]indole-9,10-dione (4d): prepared according to method A. Orange solid, 99% yield, mp 197 ℃. IR *ν* cm^{-1}): 1696 (C=O), 1655 (C=O). ^{1}H NMR ($CDCl_3$, 400 MHz): δ 7.41 (d, 1H, *J* = 7.0 Hz, 1H, Harom), 7.20 (td, 1H, *J* = 7.5 Hz, *J* = 1.2 Hz, Harom), 7.09 (td, 1H, *J* = 7.5 Hz, *J* = 1.0 Hz, Harom), 6.86 (d, 1H, *J* = 7.3 Hz, Harom), 3.73 (d, 2H, *J* = 7.8 Hz, $NCH_2CH(CH_3)_2$), 2.71 (t, 2H, *J* = 6.1 Hz, CH_2), 2.48-2.45 (m, 2H, CH_2), 2.16-2.07 (m, 3H, $NCH_2\underline{CH}(CH_3)_2$ and CH_2), 0.99 (d, 6H, *J* = 6.5 Hz, $2CH_3$). ^{13}C NMR + DEPT ($CDCl_3$, 100 MHz): δ 192.37 (C=O), 184.29 (C=O), 152.83 (Cquat), 150.23 (Cquat), 138.98 (Cquat), 135.22 (Cquat), 132.32 (CH), 128.36 (CH), 123.86 (CH), 1198.98 (Cquat), 117.70 (Cquat), 117.31 (CH), 53.21 (CH_2), 37.94 (CH_2), 30.21 (CH), 23.25 (CH_2), 22.47 (CH_2), 20.00 ($2CH_3$). HRMS calcd for $C_{19}H_{20}NO_2$ $[M+H]^+$ 294.1489, found 294.1488.

5-Isopropyl-1-methyl-5,6,7,8-tetrahydroindeno[1,2-*b*]indole-9,10-dione (4h): prepared according to method A. Orange solid, 80% yield, mp 235 ℃. IR *ν* cm^{-1}): 1693 (C=O), 1654 (C=O). ^{1}H NMR **(**DMSO-*d*$_6$, 400 MHz): δ 7.24-7.28 (m, 2H, H-2 and H-4), 6.99 (m, 1H, H-3), 4.78 (sept, 1H, *J* = 6.6 Hz, $\underline{CH}(CH_3)_2$), 2.94 (t, 2H, *J* = 6.1 Hz, H-6), 2.48 (s, 3H, CH_3), 2.38 (m, 2H, H-8), 2.10-2.04 (m, 2H, H-7), 1.57 (d, 6H, *J* = 7.0 Hz, CH_3). ^{13}C NMR + DEPT (DMSO-*d*$_6$, 100 MHz): δ 191.28 (Cquat), 185.26 (Cquat), 150.39 (2Cquat), 137.44 (Cquat), 135.20 (Cquat), 133.48 (Cquat), 132.16 (CH), 131.67 (CH), 119.82 (Cquat), 117.49 (CH), 116.68 (Cquat), 49.06 (CH), 37.63 ($2CH_2$), 22.80 (CH_2), 21.30 ($2CH_3$), 16.87 (CH_3). HRMS calcd for $C_{19}H_{19}NNaO_2$ $[M+Na]^+$ 316.1308, found 316.1303.

5-Isopropyl-1-(trifluoromethyl)-5,6,7,8-tetrahydroindeno[1,2-*b*]indole-9,10-dione (4i): prepared according to method A. Red solid, 79% yield, mp 222 ℃. IR *ν* cm^{-1}): 1711 (C=O), 1668 (C=O). ^{1}H NMR **(**DMSO-*d*$_6$, 400 MHz): δ 7.69 (d, 1H, *J* = 7.5 Hz, H-2), 7.57 (t, 1H, *J* = 7.8 Hz, H-3), 7.45 (d, 1H, *J* = 7.8 Hz, H-4), 4.80 (sept, 1H, *J* = 7.5 Hz,

CH(CH$_3$)$_2$), 2.94 (t, 2H, J = 6.0 Hz, H-6), 2.36 (dd, 2H, J = 7.3 Hz, J = 5.4 Hz, H-8), 2.09-2.02 (m, 2H, H-7), 1.55 (d, 6H, J = 7.0 Hz, CH$_3$). ^{13}C NMR + DEPT (DMSO-d_6, 100 MHz): δ 191.31 (C=O), 179.77 (C=O), 151.60 (Cquat), 150.07 (Cquat), 147.98 (Cquat), 137.05 (Cquat), 135.16 (Cquat), 133.89 (CH), 129.99 (Cquat), 125.80 ($^2J_{CF}$ = 34 Hz, C-1), 124.55 ($^3J_{CF}$ = 5.9 Hz, C-2), 123.26 ($^1J_{CF}$ = 274 Hz, CF$_3$), 122.91 (CH), 49.60 (CH), 37.60 (2CH$_2$), 22.79 (CH$_2$), 21.48 (2CH$_3$). HRMS calcd for $C_{19}H_{16}F_3NNaO_2$ [M+Na]$^+$ 370.1025, found 370.1022.

1-Bromo-5-isopropyl-5,6,7,8-tetrahydroindeno[1,2-*b*]indole-9,10-dione (4j): prepared according to method A. Orange solid, 69 % yield, mp 236 ℃. IR cm^{-1}): 1704 (C=O), 1662 (C=O), 725 (C-Br) cm^{-1}. ^{1}H NMR (DMSO-d_6, 400 MHz): δ 7.46 (d, 1H, J = 7.1 Hz, H-4), 7.36 (d, 1H, J = 8.1 Hz, H-4), 7.32 (dd, 1H, J = 8.1 Hz, J = 7.1 Hz, H-3), 4.72 (sept, 1H, J = 7.0 Hz, CH(CH$_3$)$_2$), 2.97 (t, 1H, J = 5.9 Hz, H-6), 2.87-2.85 (m, 1H, H-6), 2.40 (t, 2H, J = 6.8 Hz, H-8), 2.11-2.07 (m, 2H, H-7), 1.58 (d, 6H, J = 6.8 Hz, CH$_3$). ^{13}C NMR + DEPT (DMSO-d_6, 100 MHz): δ 191.23 (C=O), 180.71 (C=O), 151.32 (Cquat), 149.02 (Cquat), 140.76(Cquat), 137.38 (Cquat), 134.26 (CH), 133.46 (Cquat), 133.13 (CH), 118.96 (CH), 117.83 (Cquat), 116.92 (Cquat), 49.10 (CH), 41.63 (CH$_2$), 37.58 (CH$_2$), 22.72 (CH$_2$), 21.32 (2CH$_3$). HRMS calcd for $C_{18}H_{16}BrNNaO_2$ [M+Na]$^+$ 380.0257, found: 380.0245.

5-Isopropyl-5,6,7,8-tetrahydroindeno[1,2-*b*]indole-9,10-dione-1-carbonitrile (4k): prepared according to method F from **4j**. Orange solid, 72% yield, mp > 300 ℃. IR (*ν* cm^{-1}): 2227 (CN), 1709 (C=O), 1674 (C=O). ^{1}H NMR (DMSO-d_6, 400 MHz): δ 7.71 (dd, 1H, J = 6.0 Hz, J = 2.5 Hz, H-2), 7.60-7.55 (m, 2H, H-3 and H-4), 4.81 (sept, 1H, J = 7.0 Hz, CH(CH$_3$)$_2$), 2.98 (t, 2H, J = 6.0 Hz, H-6), 2.42-2.39 (m, 2H, H-8), 2.12-2.07 (m, 2H, H-7), 1.58 (d, 6H, J = 7.0 Hz, CH$_3$). ^{13}C NMR + DEPT (DMSO-d_6, 100 MHz): δ 191.3 (C=O), 179.8 (C=O), 151.8 (Cquat), 150.4 (Cquat), 150.2 (Cquat), 139.7 (Cquat), 135.7 (Cquat), 133.9 (CH), 131.2 (CH), 123.0 (CH), 117.3 (Cquat), 115.5 (CN), 106.3 (Cquat), 49.7 (CH), 37.5 (CH$_2$), 22.7 (2CH$_2$), 21.4 (2CH$_3$). HRMS calcd for $C_{19}H_{17}N_2O_2$ [M+H]$^+$ 305.1285, found 305.1282.

3-Hydroxy-5-isopropyl-5,6,7,8-tetrahydroindeno[1,2-*b*]indole-9,10-dione (4n): prepared according to method A. Red solid, 87% yield, mp 308 ℃. IR (*ν* cm^{-1}): 3322 (OH), 1702 (C=O), 1639 (C=O).^{1}H NMR (DMSO-d_6, 400 MHz): δ 10.34 (s, 1H, OH), 7.18 (d, 1H, J = 8.0 Hz, H-1), 6.84 (d, 1H, J = 1.7 Hz, H-4), 6.48 (dd, 1H, J = 8.0 Hz, J = 1.7 Hz, H-2), 4.71 (sept, 1H, J = 7.0 Hz, NCH(CH$_3$)$_2$), 2.93 (t, 2H, J = 6.0 Hz, CH$_2$), 2.38-2.35 (m, 2H, CH$_2$), 2.12-2.06 (m, 2H, CH$_2$), 1.57 (d, 6H, J = 7.0 Hz, 2CH$_3$). ^{13}C NMR + DEPT (DMSO-d_6, 100 MHz): δ 192.32 (C=O), 184.20 (C=O), 162.74 (Cquat), 151.05 (Cquat), 150.17 (Cquat), 138.17 (Cquat), 129.68 (Cquat), 125.91 (CH), 121.81 (Cquat), 117.58 (Cquat), 112.54 (CH), 110.17 (CH), 49.81 (CH), 42.30 (CH$_2$), 38.61 (CH$_2$), 23.70 (CH$_2$), 22.28 (2CH$_3$). HRMS calcd for $C_{18}H_{17}NNaO_3$ [M+Na]$^+$ 318.1101, found 318.1102.

5-Isopropyl-3-methoxy-5,6,7,8-tetrahydroindeno[1,2-*b*]indole-9,10-dione (4o): prepared according to method D. Red solid, 89% yield, mp 195 ℃. IR (*ν* cm^{-1}): 2810 (OCH$_3$), 1704 (C=O), 1694 (C=O).^{1}H NMR (DMSO-d_6, 400 MHz): δ 7.29 (d, 1H, J = 8.1 Hz, H-1), 6.90 (d, 1H, J = 2.0 Hz, H-4), 6.67 (dd, 1H, J = 8.1 Hz, J = 2.0 Hz, H-2), 4.78 (sept, 1H, J = 7.0 Hz, NCH(CH$_3$)$_2$), 3.86 (s, 3H, OCH$_3$), 2.95 (t, 2H, J = 6.0 Hz, CH$_2$), 2.39-23.35 (m, 2H, CH$_2$), 2.09-2.04 (m, 2H, CH$_2$), 1.56 (d, 6H, J = 7.0 Hz, 2CH$_3$). ^{13}C NMR + DEPT (DMSO-d_6, 100 MHz): δ 192.34 (C=O), 183.88 (C=O), 164.09 (Cquat), 151.42 (Cquat), 150.24 (Cquat), 137.67 (Cquat), 131.39 (Cquat), 125.65 (CH), 121.79

(Cquat), 117.80 (Cquat), 109.92 (CH), 109.80 (CH), 56.73 (OCH_3), 50.20 (CH), 38.59 (2CH_2), 23.75 (CH_2), 22.33 (2CH_3). HRMS calcd for $C_{19}H_{19}NNaO_3$ $[M+Na]^+$ 332.1257, found 332.1266.

(5-Isopropyl-9,10-dioxo-5,6,7,8,9,10-hexahydroindeno[1,2-*b*]indol-3-yloxy)acetic acid methyl ester (4p): prepared according to method D. Orange solid, 75% yield, mp 215 °C. IR (*v* cm^{-1}): 2847 (OCH_3), 1756 (C=O), 1698 (C=O), 1659 (C=O). ^{1}H NMR (DMSO-d_6, 400 MHz): δ 7.28 (d, 1H, *J* = 8.1 Hz, H-1), 6.93 (bs, 1H, H-4), 6.64 (dd, 1H, *J* = 8.1 Hz, *J* = 2.1 Hz, H-2), 4.94 (s, 2H, OCH_2), 4.80 (sept, 1H, *J* = 6.9 Hz, NC<u>H</u>(CH_3)$_2$), 3.75 (s, 3H, OCH_3), 2.96 (t, 2H, *J* = 6.0 Hz, CH_2), 2.40-2.36 (m, 2H, CH_2), 2.10-2.05 (m, 2H, CH_2), 1.57 (d, 6H, *J* = 7.0 Hz, 2 CH_3). ^{13}C NMR + DEPT (DMSO-d_6, 100 MHz): δ 192.44 (C=O), 183.77 (C=O), 169.71 (C=O), 162.32 (Cquat), 151.59 (Cquat), 150.27 (Cquat), 137.77 (Cquat), 132.01 (Cquat), 125.50 (Cquat), 125.49 (CH), 117.88 (Cquat), 110.99 (CH), 109.98 (CH), 65.92 (OCH_2), 52.89 (OCH_3), 49.51 (CH), 38.58 (2CH_2), 23.76 (CH_2), 22.36 (2CH_3). HRMS calcd for $C_{21}H_{21}NNaO_5$ $[M+Na]^+$: 390.1312; found 390.1313.

5-Isopropyl-9,10-dioxo-5,6,7,8,9,10-hexahydroindeno[1,2-*b*]indol-3-yl acetate (4q): prepared according to method D. Orange solid, 72% yield, mp 225 °C. IR (*v* cm^{-1}): 1760 (C=O), 1704 (C=O), 1663 (C=O). ^{1}H NMR **(**$CDCl_3$, 400 MHz): δ 7.42 (d, 1H, *J* = 7.8 Hz, H-1), 6.84 (d, 1H, *J* = 1.8 Hz, H-4), 6.79 (dd, 1H, *J* = 7.8 Hz, *J* = 1.8 Hz , H-2), 4.56 (sept, 1H, *J* = 7.1 Hz, NC<u>H</u>(CH_3)$_2$), 2.84 (t, 2H, *J* = 6.1 Hz, CH_2), 2.48-2.45 (m, 2H, CH_2), 2.31 (s, 3H, CH_3), 2.19-2.13 (m, 2H, CH_2), 1.61 (d, 6H, *J* = 7.0 Hz, 2CH_3). ^{13}C NMR + DEPT ($CDCl_3$, 100 MHz): δ 192.24 (C=O), 183.23 (C=O), 169.12 (C=O), 153.85 (Cquat), 150.06 (Cquat), 149.37 (Cquat), 137.08 (Cquat), 136.17 (Cquat), 124.76 (CH), 122.00 (Cquat), 120.07 (CH), 118.01 (Cquat), 113.54 (CH), 49.62 (CH), 37.87 (2CH_2), 23.33 (CH_2), 22.15 (2CH_3), 21.32 (CH_3). HRMS calcd for $C_{20}H_{19}NNaO_4$ $[M+Na]^+$ 360.1206, found 360.1199.

4-(5-Isopropyl-9,10-dioxo-5,6,7,8,9,10-hexahydroindeno[1,2-*b*]indol-3-yloxy)butyric acid methyl ester (4r): prepared according to method D. Orange solid, 77% yield, mp 154 °C. IR (*v* cm^{-1}): 1732 (C=O), 1699 (C=O), 1663 (C=O). ^{1}H NMR ($CDCl_3$, 400 MHz): δ 7.36 (d, 1H, *J* = 8.1 Hz, H-1), 6.66 (d, 1H, *J* = 2.1 Hz, H-4), 6.44 (dd, 1H, *J* = 8.1 Hz, *J* = 2.1 Hz, H-2), 4.56 (sept, 1H, *J* = 7.0 Hz, NC<u>H</u>(CH_3)$_2$), 4.01 (t, 2H, *J* = 6.2 Hz, OCH_2), 3.69 (s, 3H, OCH_3), 2.85 (t, 2H, *J* = 6.2 Hz, CH_2), 2.53 (t, 2H, *J* = 7.2 Hz, CH_2), 2.46 (m, 2H, CH_2), 2.18-2.06 (m, 4H, 2CH_2), 1.62 (d, 6H, *J* = 7.1 Hz, 2CH_3). ^{13}C NMR + DEPT ($CDCl_3$, 100 MHz): 192.55 (C=O), 184.10 (C=O), 173.76 (C=O), 162.62 (Cquat), 149.94 (Cquat), 149.21 (Cquat), 148.83 (Cquat), 137.61 (Cquat), 131.77 (Cquat), 125.60 (CH), 118.03 (Cquat), 109.78 (CH), 108.84 (CH), 67.46 (OCH_2), 52.04 (OCH_3), 49.70 (CH), 38.10 (CH_2), 30.72 (CH_2), 24.75 (CH_2), 23.84 (CH_2), 23.57 (CH_2), 22.24 (2CH_3). HRMS calcd for $C_{23}H_{25}NNaO_5$ $[M+Na]^+$ 418.1625, found 418.1607.

4-(5-Isopropyl-9,10-dioxo-5,6,7,8,9,10-hexahydroindeno[1,2-*b*]indol-3-yloxy)butyric acid (4s): prepared according to method E from **4r**. Orange solid, 75% yield, mp 222 °C. IR (*v* cm^{-1}): 1732 (C=O), 1696 (C=O). ^{1}H NMR (DMSO-d_6, 400 MHz): *δ* 12.19 (s, 1H, OH), 7.28 (d, 1H, *J* = 8.1 Hz, H-1), 6.91 (s, 1H, H-4), 6.66 (dd, 1H, *J* = 8.1 Hz, *J* = 1.8 Hz, H-2), 4.78 (m, 1H, NC<u>H</u>(CH_3)$_2$), 4.08 (t, 2H, *J* = 6.3 Hz, OCH_2), 2.95 (t, 2H, *J* = 6.0 Hz, CH_2), 2.44 (d, 2H, *J* = 7.2 Hz, CH_2), 2.37 (m, 2H, CH_2), 2.08 (m, 2H, CH_2), 2.02-1.98 (m, 2H, CH_2), 1.57 (d, 6H, *J* = 7.0 Hz, 2 CH_3). ^{13}C NMR + DEPT (DMSO-d_6, 100 MHz): δ 192.29 (C=O), 183.83 (C=O), 174.96 (C=O), 163.36 (Cquat),

159.99 (Cquat), 151.34 (Cquat), 150.33 (Cquat), 137.71 (Cquat), 131.35 (Cquat), 125.63 (CH), 117.88 (Cquat), 110.32 (CH), 110.08 (CH), 68.20 (OCH_2), 50.32 (CH), 38.60 (CH_2), 30.95 (CH_2), 25.01 (CH_2), 23.96 (CH_2), 23.77 (CH_2), 22.37 (2 CH_3). HRMS calcd for $C_{22}H_{23}NNaO_5$ $[M+Na]^+$ 404.1468, found 404.1457.

1,3-Dibromo-5-isopropyl-5,6,7,8-tetrahydroindeno[1,2-*b*]indole-9,10-dione (4w): prepared according to method A. Orange solid, 84% yield, mp 276 ℃. IR *ν* cm^{-1}): 1713 (C=O), 774 (C-Br) cm^{-1}. ^{1}H NMR **(**DMSO-d_6, 400 MHz): δ 7.56 (d, 1H, *J* = 1.4 Hz, H-2), 7.54 (d, 1H, *J* = 1.4 Hz, H-4), 4.81 (sept, 1H, *J* = 7.0 Hz, <u>CH</u>(CH_3)$_2$), 2.95 (t, 2H, *J* = 7.0, CH_2), 2.35 (t, 2H, *J* = 6.1 Hz, CH_2), 2.02-2.08 (m, 2H, CH_2), 1.54 (d, 6H, *J* = 7.0 Hz, CH_3). ^{13}C NMR + DEPT (DMSO-d_6, 100 MHz): δ 190.78 (C=O), 179.40 (C=O), 151.36 (Cquat), 147.43 (Cquat), 138.50 (Cquat), 134.11 (CH), 132.41 (Cquat), 126.53 (Cquat), 120.88 (CH), 120.22 (Cquat), 117.94 (Cquat), 117.12 (Cquat), 49.79 (CH), 37.30 (CH_2), 23.11 (CH_2), 22.52 (CH_2), 21.15 (2CH_3). HRMS calcd for $C_{18}H_{16}Br_2NO_2$ $[M+H]^+$ 435.9542, found 435.9530.

General procedures for the synthesis of phenol derivatives **5**

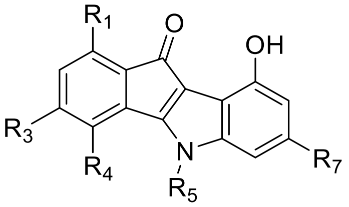

Figure S3. Structure of phenol derivatives **5**.

General procedures for the synthesis of 9-hydroxy-5*H*-indeno[1,2-*b*]indol-10-ones

Method A: To a solution of ketone **4** (1.95 mmol) in Ph_2O (7.5 mL) was added 10% Pd/C (3.90 mmol). Then, the mixture was heated to reflux for 3-6 h. After cooling, 25 mL of MeOH were added and the solution filtered on celite. Evaporation of the solvent left a residue which was purified by silica gel column chromatography.

Method B: A sealed vessel, charged with ketone **4** (0.61 mmol), 2,3-dichloro-5,6-dicyano-1,4-benzoquinone (DDQ) (0.79 mmol) and dioxane (16 mL) was heated to 140 °C for 12 min under microwave irradiation. The mixture was then filtered. The filtrate was concentrated and the residue was purified by silica gel column chromatography using dichloromethane/cyclohexane (1:1.5, v/v) as the eluent.

9-Hydroxy-5*H*-indeno[1,2-*b*]indol-10-one (5a): prepared according to method A. The product was purified by chromatography on neutralized silica gel, using EtOAc/cyclohexane/triethylamine (50:1:1, v/v/v) as the eluent. Dark red solid, 45% yield, mp 311 °C. IR (ν cm^{-1}): 3360 (NH, OH), 1655 (C=O). ^1H NMR (DMSO-d_6, 400 MHz): δ 12.38 (s, 1H, NH), 9.42 (s, 1H, OH), 7.35 (t, 1H, J = 7.2 Hz, Harom), 7.29 (d, 1H, J = 7.2 Hz, Harom), 7.24-7.20 (m, 2H, Harom), 6.94 (td, 1H, J = 7.9 Hz, J = 1.2 Hz, Harom), 6.87 (d, 1H, J = 8.1 Hz, Harom), 6.54 (d, 1H, J = 7.7 Hz, Harom). ^{13}C NMR + DEPT (DMSO-d_6, 100 MHz): δ 183.59 (C=O), 157.68 (Cquat), 151.38 (Cquat), 143.82 (Cquat), 140.59 (Cquat), 135.00 (Cquat), 132.73 (CH), 129.72 (CH), 124.66 (CH), 122.57 (CH), 118.71 (CH), 114.21 (Cquat), 113.32 (Cquat), 108.07 (CH), 104.81 (CH). HRMS calcd for $C_{15}H_9NNaO_2$ $[M+Na]^+$ 258.0525, found 258.0520.

9-Hydroxy-5-methyl-5*H*-indeno[1,2-*b*]indol-10-one (5b): prepared according to method A. The product was purified by silica gel chromatography using dichloromethane/methanol (99:1, v/v) as the eluent. Orange solid, 42% yield, mp 223 °C. IR (ν cm^{-1}): 3230 (OH), 1654 (C=O). ^1H NMR (DMSO-d_6, 400 MHz): δ 9.48 (s, 1H, OH), 7.50 (dt, 1H, J = 7.2 Hz, J = 0.9 Hz, Harom), 7.34 (td, 1H, J = 7.4 Hz, J = 1.3 Hz, Harom), 7.31-7.29 (m, 1H, Harom), 7.23 (dt, 1H, J = 7.4 Hz, J = 1.0 Hz, Harom), 7.00 (t, 1H, J = 7.6 Hz, Harom), 6.94 (dd, 1H, J = 8.3 Hz, J = 1.0 Hz, Harom), 6.58 (dd, 1H, J = 7.6 Hz, J = 1.0 Hz, Harom), 3.94 (s, 3H, CH_3). ^{13}C NMR + DEPT (DMSO-d_6, 100 MHz): δ 182.66 (C=O), 157.49 (Cquat), 151.05 (Cquat), 144.76 (Cquat), 140.31 (Cquat), 134.27 (Cquat), 132.28 (CH), 129.49 (CH), 124.27 (CH), 122.34 (CH), 119.12 (CH), 113.11 (Cquat), 112.53 (Cquat), 108.14 (CH), 102.75 (CH), 31.92 (CH_3). HRMS calcd for $C_{16}H_{11}NNaO_2$ $[M+Na]^+$ 272.0682, found 272.0693.

5-Ethyl-9-hydroxy-5*H*-indeno[1,2-*b*]indol-10-one (5c): prepared according to method A. The product was purified by silica gel chromatography using dichloromethane/methanol (99:1, v/v) as the eluent. Orange solid, 49% yield, mp 202 °C. IR (ν cm^{-1}): 3429 (OH), 1662 (C=O). ^{1}H NMR (DMSO-d_6, 400 MHz): δ 9.48 (s, 1H, OH), 7.49 (d, 1H, *J* = 6.8 Hz, Harom), 7.36 (td, 1H, *J* = 7.4 Hz, Harom), 7.32 (d, 1H, *J* = 7.2 Hz, Harom), 7.25 (t, 1H, *J* = 7.3 Hz, Harom), 7.01-6.98 (m, 2H, Harom), 6.60-6.56 (m, 1H, Harom), 4.41 (q, 2H, *J* = 7.2 Hz, CH_2), 1.38 (t, 3H, *J* = 7.2 Hz, CH_3). ^{13}C NMR + DEPT (DMSO-d_6, 100 MHz): δ 182.65 (C=O), 156.59 (Cquat), 151.19 (Cquat), 143.62 (Cquat), 140.26 (Cquat), 134.03 (Cquat), 132.46 (CH), 129.51 (CH), 124.38 (CH), 122.39 (CH), 118.97 (CH), 113.38 (Cquat), 112.69 (Cquat), 108.06 (CH), 102.63 (CH), 39.93 (CH_2), 15.44 (CH_3). HRMS calcd for $C_{17}H_{13}NNaO_2$ [M+Na]$^+$ 286.0838, found 286.0827.

1-Bromo-9-Hydroxy-5-isopropyl-5*H*-indeno[1,2-*b*]indol-10-one (5e): prepared according to method B. Orange solid, mp 187 °C, 44 % yield, IR (ν cm^{-1}): 3391 (OH), 1673 (C=O), 726 (C-Br). ^{1}H NMR ($CDCl_3$, 400 MHz): δ 7.25 (dd, 1H, *J* = 8.3 Hz, *J* = 0.8 Hz, Harom), 7.19 (dd, 1H, *J* = 7.3 Hz, *J* = 0.8 Hz, Harom), 7.10 (dd, 1H, *J* = 8.3 Hz, *J* = 7.3 Hz, Harom), 7.07 (t, 1H, *J* = 8.1 Hz, Harom), 6.94 (d, 1H, *J* = 8.3 Hz, Harom), 6.86 (s, 1H, OH), 6.65 (d, 1H, *J* = 7.8 Hz, Harom), 4.85 (sept, 1H, *J* = 7.1 Hz, <u>CH</u>(CH_3)$_2$), 1.73 (d, 6H, *J* = 7.0 Hz, 2CH_3). ^{13}C NMR + DEPT ($CDCl_3$, 100 MHz): δ 184.02 (C=O), 152.99 (Cquat), 150.47 (2Cquat), 142.87 (Cquat), 138.81 (Cquat), 136.65 (Cquat), 134.58 (CH), 133.20 (CH), 125.75 (CH), 119.10 (CH), 116.59 (Cquat), 113.60 (Cquat), 107.88 (CH), 104.99 (CH), 50.07 (CH), 21.88 (2CH_3). HRMS calcd for $C_{18}H_{15}BrNO_2$ [M+H]$^+$ 356.0281, found 356.0274.

9-Hydroxy-5-isopropyl-10-oxo-5,10-dihydroindeno[1,2-*b*]indole-1-carbonitrile (5f): prepared according to method A. The product was purified by silica gel chromatography using dichloromethane/acetone (100:6, v/v) as the eluent. Red solid, 59% yield, mp 301 °C, degradation. IR (ν cm^{-1}): 3402 (OH), 2232 (CN), 1671 (C=O). ^{1}H NMR ($CDCl_3$, 400 MHz): δ 7.41-7.32 (m, 3H, Harom), 7.12 (t, 1H, *J* = 8.1 Hz, Harom), 6.96 (d, 1H, *J* = 8.3 Hz, Harom), 6.68 (d, 1H, *J* = 7.8 Hz, Harom), 6.60 (s, 1H, OH), 4.87 (sept, 1H, *J* = 6.8 Hz, <u>CH</u>(CH_3)$_2$), 1.76 (d, 6H, *J* = 6.8 Hz, CH_3). ^{13}C NMR + DEPT ($CDCl_3$, 100 MHz): δ 181.72 (C=O), 153.76 (Cquat), 152.52 (Cquat), 150.64 (Cquat), 142.88 (Cquat), 137.43 (Cquat), 133.00 (CH), 131.98 (CH), 126.47 (2CH), 122.66 (CH), 115.21 (Cquat), 108.66 (CH), 108.00 (Cquat), 105.23 (Cquat), 102.75 (Cquat), 50.33 (CH), 22.16 (2CH_3). HRMS calcd for $C_{19}H_{14}N_2NaO_2$ [M+Na]$^+$ 325.0947, found 325.0939.

1,9-Dihydroxy-5-isopropyl-5*H*-indeno[1,2-*b*]indol-10-one (5g): prepared according to method A from **4l** [1]. The product was purified by silica gel chromatography using dichloromethane/methanol (99:1, v/v) as the eluent. Red solid, 73% yield, mp 186 °C. IR (ν cm^{-1}): 3371 (se, OH), 1731 (C=O), 1650 (C=O). ^{1}H NMR ($CDCl_3$, 400 MHz): δ 8.36 (s, 1H, OH), 7.14 (dd, 1H, *J* = 8.6 Hz, *J* = 7.2 Hz, Harom), 7.07 (t, 1H, *J* = 8.2 Hz, Harom), 6.94 (d, 1H, *J* = 8.3 Hz, Harom), 6.80 (d, 1H, *J* = 7.3 Hz, Harom), 6.70 (d, 1H, *J* = 8.6 Hz, Harom), 6.68 (d, 1H, *J* = 7.8 Hz, H-8), 6.33 (se, 1H, OH), 4.82 (sept, 1H, *J* = 6.8 Hz, <u>CH</u>(CH_3)$_2$), 1.71 (d, 6H, *J* = 7.1 Hz, 2CH_3). ^{13}C NMR + DEPT ($CDCl_3$, 100 MHz): δ 188.59 (C=O), 155.97 (Cquat), 154.98 (Cquat), 149.89 (Cquat), 142.64 (Cquat), 135.74 (Cquat), 134.80 (CH), 125.19 (CH), 120.86 (Cquat), 120.76 (CH), 114.77 (Cquat), 113.93 (CH), 113.42 (Cquat), 107.86 (CH), 104.97 (CH), 49.92 (CH), 21.71 (2CH_3). HRMS calcd for $C_{18}H_{16}NO_3$ [M+H]$^+$ 294.1125, found: 294.1128.

9-Hydroxy-5-isopropyl-3-methoxy-5*H*-indeno[1,2-*b*]indol-10-one (5h): prepared according to method A. The product was purified by silica gel chromatography using dichloromethane/methanol (99:1, v/v) as the eluent. Orange solid, mp 208 °C, 62% yield, IR (*ν* cm^{-1}): 3387 (OH), 1659 (C=O). ^{1}H NMR ($CDCl_3$, 400 MHz): δ 7.34 (d, 1H, *J* = 8.1 Hz, H-1), 7.05 (t, 1H, *J* = 8.0 Hz, H-7), 6.91 (d, 1H, *J* = 8.1 Hz, H-6), 6.83 (s, 1H, OH), 6.80 (d, 1H, *J* = 2.0 Hz, H-4), 6.65 (d, 1H, *J* = 7.6 Hz, H-8), 6.50 (dd, 1H, *J* = 8.0 Hz, *J* = 2.0 Hz, H-2), 4.81 (m, 1H, NC<u>H</u>(CH_3)$_2$), 3.85 (s, 3H, CH_3), 1.70 (d, 6H, *J* = 7.1 Hz, 2CH_3). ^{13}C NMR + DEPT ($CDCl_3$, 100 MHz): δ 186.20 (C=O), 163.62 (Cquat), 153.66 (Cquat), 150.57 (Cquat), 142.87 (Cquat), 138.74 (Cquat), 133.03 (Cquat), 125.50 (CH), 125.24 (CH), 117.49 (Cquat), 113.93 (Cquat), 111.34 (CH), 108.86 (CH), 107.65 (CH), 104.96 (CH), 56.13 (OCH_3), 49.94 (CH), 22.16 (2CH_3). HRMS calcd for $C_{19}H_{17}NNaO_3$ $[M+Na]^+$ 330.1101, found 330.1101.

9-Hydroxy-5-isopropyl-4-methoxy-5*H*-indeno[1,2-*b*]indol-10-one (5i): prepared according to method A from **4u** [8]. The product was purified by silica gel chromatography using dichloromethane/methanol (95:5, v/v) as the eluent. Orange solid, mp 225 °C, 53 % yield, IR (*ν* cm^{-1}): 3471 (OH), 1662 (C=O). ^{1}H NMR (DMSO-d_6, 400 MHz): δ 9.04 (s, 1H, OH), 7.31 (dd, 1H, *J* = 8.6 Hz, *J* = 7.0 Hz, H-2), 7.20 (dd, 1H, *J* = 8.6 Hz, *J* = 0.8 Hz, H-1), 7.16 (d, 1H, *J* = 8.3 Hz, H-6), 7.04 (dd, 1H, *J* = 6.8 Hz, *J* = 0.8 Hz, H-3), 6.98 (t, 1H, *J* = 8.1 Hz, H-7), 6.59 (1H, d, *J* = 7.8 Hz, H-8), 5.89-5.85 (m, 1H, H-iPr), 3.99 (s, 3H, OCH_3), 1.66 (d, 6H, *J* = 7.0 Hz, 2CH_3). ^{13}C NMR + DEPT (DMSO-d_6, 100 MHz): δ 182.63 (C=O), 157.52 (Cquat), 150.90 (Cquat), 150.53 (Cquat), 141.76 (Cquat), 141.68 (Cquat), 131.78 (CH), 123.82 (CH), 120.76 (Cquat), 118.75 (CH), 115.84 (CH), 113.91 (Cquat), 112.81 (Cquat), 107.57 (CH), 106.10 (CH), 56.36 (OCH_3), 50.38 (CH), 20.88 (2CH_3). HRMS calcd for $C_{19}H_{17}NNaO_3$ $[M+Na]^+$ 330.1101, found 330.1102.

General procedure for the synthesis of *para*-quinone derivatives **6**

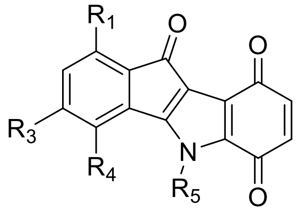

Figure S4. Structure of *para*-quinone derivatives **6**.

General procedure for the synthesis of 5*H*-indeno[1,2-*b*]indole-6,9,10-triones

An aqueous solution (30 mL) of Frémy's salt (4 mmol) and KH_2PO_4 (0.24 mmol) was added to a solution of 9-hydroxyindeno[1,2-*b*]indol-10-one **5** (1 mmol) in acetone (30 mL). The oxidative effect of Frémy's salt is much more pronounced if added in small portions during the reaction. The reaction mixture was stirred at room temperature for a variable time, extracted with CH_2Cl_2, dried over Na_2SO_4 and concentrated under vacuum. The crude residue was purified by column chromatography on silica gel CH_2Cl_2/Acetone (98:2, v / v) as eluent.

5*H*-Indeno[1,2-*b*]indole-6,9,10-trione (6a): dark red solid, 67% yield, mp 349 ℃. IR (*ν* cm^{-1}): 3222 (NH), 1696 (C=O), 1685 (C=O), 1649 (C=O). ^1H NMR (DMSO-d_6, 300 MHz): δ 13.74 (bs, 1H, NH), 7.49 (td, 1H, *J* = 7.4 Hz, *J* = 1.1 Hz, Harom), 7.45 (d, 1H, *J* = 7.0 Hz, Harom), 7.40 (d, 1H, *J* = 7.2 Hz, Harom), 7.34 (td, 1H, *J* = 7.4 Hz, *J* = 1.1 Hz, Harom), 6.73 (d, 1H, *J* = 10.3 Hz, AB-system, H-7 or H-8), 6.66 (d, 1H, *J* = 10.3 Hz, AB-system, H-7 or H-8). ^{13}C NMR + DEPT (DMSO-d_6, 125 MHz): δ 183.75 (C=O), 181.67 (C=O), 177.59 (C=O), 155.10 (Cquat), 138.93 (Cquat), 137.03 (CH), 136.03 (CH), 136.01 (Cquat), 133.91 (CH), 133.60 (Cquat), 130.04 (CH), 123.54 (CH), 120.86 (Cquat), 119.90 (CH), 119.86 (Cquat). HRMS calcd for $C_{15}H_7NNaO_3$ $[M+Na]^+$ 272.0318, found 272.0318.

5-Methyl-5*H*-indeno[1,2-*b*]indole-6,9,10-trione (6b): orange solid, 65% yield, mp 254 ℃. IR (*ν* cm^{-1}): 1710 (C=O), 1697 (C=O), 1653 (C=O). ^1H NMR ($CDCl_3$, 300 MHz): δ 7.63 (m, 1H, Harom), 7.43 (m, 1H, Harom), 7.37-7.30 (m, 2H, Harom), 6.66 (d, 1H, *J* = 10.3 Hz, AB-system, H-7 or H-8), 6.60 (d, 1H, *J* = 10.3 Hz, AB-system, H-7 or H-8), 4.23 (s, 3H, CH_3). ^{13}C NMR + DEPT (DMSO-d_6/$CDCl_3$ 1/1, 125 MHz): δ 180.94 (C=O), 177.70 (C=O), 174.30 (C=O), 159.07 (Cquat), 139.55 (Cquat), 138.93 (Cquat), 136.55 (CH), 135.72 (CH), 133.05 (Cquat), 132.0 (CH), 129.67 (Cquat), 129.22 (CH), 123.80 (CH), 119.14 (CH), 110.98 (Cquat), 34.33 (CH_3). HRMS calcd for $C_{16}H_9NNaO_3$ $[M+Na]^+$ 286.0475, found 286.0464.

5-Ethyl-5*H*-indeno[1,2-*b*]indole-6,9,10-trione (6c): orange solid, 70% yield, mp 263 ℃. IR (*ν* cm^{-1}): 1712 (C=O), 1660 (C=O), 1648 (C=O). ^1H NMR (DMSO-d_6, 300 MHz): δ 7.63 (d, 1H, *J* = 7.3 Hz, Harom), 7.54 (td, 1H, *J* = 7.4 Hz, *J* = 1.6 Hz, Harom), 7.52 (d, 1H, *J* = 7.2 Hz, Harom), 7.40 (t, 1H, *J* = 7.4 Hz, Harom), 6.73 (d, 1H, *J* = 10.3 Hz, AB-system, H-7 or H-8), 6.69 (d, 1H, *J* = 10.3 Hz, AB-system, H-7 or H-8), 4.62 (q, 2H, *J* = 7.2 Hz, $\underline{CH_2}CH_3$), 1.43 (t, 3H, *J* = 7.2 Hz, $CH_2\underline{CH_3}$). ^{13}C NMR + DEPT (DMSO-d_6, 125 MHz): δ 183.09 (C=O), 181.48 (C=O), 177.90 (C=O), 154.78 (Cquat), 138.97 (Cquat), 137.30 (CH), 136.09 (CH), 134.13 (CH), 133.34 (Cquat), 132.96 (Cquat),

130.29 (CH), 123.84 (CH), 121.64 (Cquat), 120.47 (CH), 119.14 (Cquat), 42.67 (CH_2), 15.38 (CH_3). HRMS calcd for $C_{17}H_{11}NNaO_3$ [M+Na]$^+$ 300.0631, found 300.0622.

1-Bromo-5-isopropyl-5*H*-indeno[1,2-*b*]indole-6,9,10-trione (6d): orange solid, 35% yield, mp 285 °C. IR (ν cm^{-1}): 1719 (C=O), 1662 (C=O), 1649 (C=O). ^{1}H NMR (DMSO-d_6 + $CDCl_3$ drop, 500 MHz): δ 7.68 (d, 1H, *J* = 7.6 Hz, H-4), 7.52 (d, 1H, *J* = 8.2 Hz, H-2), 7.44 (t, 1H, *J* = 8.0 Hz, H-3), 6.72 (d, 1H, *J* = 10.1 Hz, AB syst., H-7 or H-8), 6.68 (d, 1H, *J* = 10.1 Hz, AB syst., H-7 or H-8), 5.64 (m, 1H, CH(CH_3)$_2$), 1.68 (d, 6H, *J* = 6.9 Hz, CH_3). ^{13}C NMR + DEPT (DMSO-d_6 + $CDCl_3$ drop, 125 MHz): δ 180.70 (C=O), 179.76 (C=O), 177.06 (C=O), 151.81 (Cquat), 137.46 (CH), 135.96 (Cquat), 135.00 (CH), 134.68 (Cquat), 134.49 (CH), 134.24 (CH), 134.00 (Cquat), 133.06 (Cquat), 122.24 (Cquat), 120.72 (CH), 118.48 (Cquat), 50.38 (CH), 19.98 (2CH_3). HRMS calcd for $C_{18}H_{12}BrNNaO_3$ [M+Na]$^+$ 391.9893, found 391.9887.

5-Isopropyl-3-methoxy-5*H*-indeno[1,2-*b*]indole-6,9,10-trione (6e): orange solid, 39% yield, mp 232 °C. IR (ν cm^{-1}): 1704 (C=O), 1659 (C=O), 1642 (C=O). ^{1}H NMR ($CDCl_3$, 400 MHz): δ 7.60 (d, 1H, *J* = 8.2 Hz, H-4), 6.97 (d, 1H, *J* = 2.1 Hz, H-1), 6.71 (dd, 1H, *J* = 8.2 Hz, *J* = 2.1 Hz, H-3), 6.63 (d, 1H, *J* = 10.2 Hz, AB syst.), 6.60 (d, 1H, *J* = 10.2 Hz, AB syst.), 5.85-5.55 (m, 1H, NCH(CH_3)$_2$), 3.90 (s, 3H, OCH_3), 1.67 (d, 6H, *J* = 7.0 Hz, 2CH_3). ^{13}C NMR + DEPT ($CDCl_3$, 100 MHz): δ 183.05 (C=O), 181.87 (C=O), 178.10 (C=O), 164.01 (Cquat), 153.19 (Cquat), 138.06 (2CH), 136.18 (Cquat), 135.75 (CH), 133.54 (Cquat), 132.78 (Cquat), 131.12 (Cquat), 126.51 (CH), 111.65 (Cquat), 110.63 (CH), 56.11 (OCH_3), 29.84 (CH), 21.09 (2CH_3). HRMS calcd for $C_{19}H_{15}NNaO_4$ [M+Na]$^+$ 344.0893, found 344.0895.

5-Isopropyl-4-methoxy-5*H*-indeno[1,2-*b*]indole-6,9,10-trione (6f): red solid, 22% yield, mp 264 °C. IR (ν cm^{-1}): 1715 (C=O), 1664 (C=O), 1642 (C=O). ^{1}H NMR (DMSO-d_6, 500 MHz, 60 °C): δ 7.43 (dd, 1H, *J* = 8.5 Hz, *J* = 6.9 Hz, H-2), 7.33 (d, 1H, *J* = 8.5 Hz, H-1), 7.19 (dd, 1H, *J* = 6.9 Hz, H-4), 6.73 (d, 1H, *J* = 10.1 Hz, AB syst.), 6.67 (d, 1H, *J* = 10.1 Hz, AB syst.), 5.92 (sept, 1H, *J* = 6.9 Hz, NCH(CH_3)$_2$), 4.01 (s, 3H, OCH_3), 1.62 (d, 6H, *J* = 6.9Hz, 2CH_3). ^{13}C NMR + DEPT (DMSO-d_6, 125 MHz, 60 °C): δ 182.28 (C=O), 181.24 (C=O), 175.99 (C=O), 151.29 (Cquat), 148.27 (Cquat), 140.52 (Cquat), 138.01 (2CH), 134.45 (Cquat), 134.44 (CH), 132.18 (Cquat), 120.10 (Cquat), 119.32 (CH), 116.52 (CH), 116.52 (Cquat), 56.22 (OCH_3), 52.53 (CH), 19.91 (2CH_3). HRMS calcd for $C_{19}H_{15}NNaO_4$ [M+Na]$^+$ 344.0893.

References for supporting information

1. Jabor Gozzi, G., et al., *Converting potent indeno[1,2-b]indole inhibitors of protein kinase CK2 into selective inhibitors of the breast cancer resistance protein ABCG2.* J Med Chem, 2015. **58**(1): p. 265-77.
2. Hemmerling H J., R.G., *Partially Saturated Indeno[1,2-b]indole Derivatives via Deoxygenation of Heterocyclic α-Hydroxy-N,O-hemiaminals.* Synthesis, 2009(6): p. 985-999
3. Liangzhen Cai , X.L., Xiaochun Tao & Dong Shen *Efficient Microwave Assisted Cyanation of Aryl Bromide.*

   Synthetic Comm 2003. **34**(7): p. 1215-1221.
4. Opsenica, D., et al., *Cholic acid derivatives as 1,2,4,5-tetraoxane carriers: structure and antimalarial and antiproliferative activity.* J Med Chem, 2000. **43**(17): p. 3274-82.
5. Ambroise Poumaroux, Z.B., Monique Domard, and Houda Fillion, *Regiospecific Hetero Diels-Alder Synthesis of Pyrido[2,3-b]- and Pyrido[3,2-b]carbazole-5,11-diones.* Heterocycles, 1997. **45**(3): p. 585-596.
6. Hundsdorfer, C., et al., *Indeno[1,2-b]indole derivatives as a novel class of potent human protein kinase CK2 inhibitors.* Bioorg Med Chem, 2012. **20**(7): p. 2282-9.
7. Zimmer H., L.D.C., Horgan S.W., *Oxidations with potassium nitrosodisulfonate (Fremy's radical). The Teuber reaction.* Chem Rev 1971. **71**(2): p. 229-246.
8. Bloch, S., et al., *Inhibition of Shiga toxin-converting bacteriophage development by novel antioxidant compounds.* J Enzyme Inhib Med Chem, 2018. **33**(1): p. 639-650.
9. Alchab, F., et al., *Synthesis, Biological Evaluation and Molecular Modeling of Substituted Indeno[1,2-b]indoles as Inhibitors of Human Protein Kinase CK2.* Pharmaceuticals, 2015. **8**(2): p. 279-302.
