# Supplementary material for: QSAR Model of Indeno[1,2-b]indole Derivatives and Identification of N-isopentyl-2-methyl-4,9-dioxo-4,9-Dihydronaphtho[2,3-b]furan-3-carboxamide as a Potent CK2 Inhibitor
Source: Molecules. 2019 Dec 26;25(1):97. doi: 10.3390/molecules25010097 (PMC6982966; doi:10.3390/molecules25010097)
Supplement: Supplementary file 1 [file molecules-25-00097-s001.zip › SMILES.pdf]

Table 1. Chemical structures of the indeno[1,2-*b*]indoles used for the training set in SMILES format.

Nr.	Chemical structure	Nr.	Chemical structure
**4e**	O=C1C2=C(N(CCC3=CC=CC=C3OC)C(CCC4)=C2C4=O)C5=C1C=CC=C5	**4y**	O=C1C2=C(N(C(C)C)C(CC(C(C)C)C3)=C2C3=O)C4=C1C=CC=C4
**4f**	O=C1CCCC2=C1C(C3=O)=C(N2CCC4=CC=C(OC)C=C4)C5=C3C=CC=C5	**5d**	O=C1C2=C(N(C(C)C)C3=C2C(O)=CC=C3)C4=C1C=CC=C4
**4g**	O=C1C2=C(N(C(C)C)C(CCC3)=C2C3=O)C4=C1C=CC=C4	**5g**	CC(C)N(C1=C2C(O)=CC=C1)C(C3=C4C(O)=CC=C3)=C2C4=O
**4h**	O=C1C2=C(N(C(C)C)C(CCC3)=C2C3=O)C4=C1C(C)=CC=C4	**5h**	O=C1C2=C(N(C(C)C)C3=C2C(O)=CC=C3)C4=C1C=CC(OC)=C4
**4p**	O=C1C2=C(N(C(C)C)C(CCC3)=C2C3=O)C4=C1C=CC(OCC(OC)=O)=C4	**5j**	O=C1C2=C(N(C(C)C)C3=C2C(O)=CC(C)=C3)C4=C1C=CC=C4
**4r**	O=C1C2=C(N(C(C)C)C(CCC3)=C2C3=O)C4=C1C=CC(OCCCC(OC)=O)=C4	**5k**	O=C1C2=C(N(C(C)C)C3=C2C(O)=CC(C(C)C)=C3)C4=C1C=CC=C4
**4s**	O=C1C2=C(N(C(C)C)C(CCC3)=C2C3=O)C4=C1C=CC(OCCCC(O)=O)=C4	**6b**	O=C1C2=C(N(C)C(C(C=C3)=O)=C2C3=O)C4=C1C=CC=C4
**4v**	O=C1C2=C(N(C(C)C)C(CCC3)=C2C3=O)C4=C1C=CC=C4OC/C=C(C)\C	**6d**	O=C1C2=C(N(C(C)C)C(C(C=C3)=O)=C2C3=O)C4=C1C(Br)=CC=C4
**4w**	O=C1C2=C(N(C(C)C)C(CCC3)=C2C3=O)C4=C1C(Br)=CC(Br)=C4	**6f**	O=C1C2=C(N(C(C)C)C(C(C=C3)=O)=C2C3=O)C4=C1C=CC=C4OC
**4x**	O=C1C2=C(N(C(C)C)C(CC(C)C3)=C2C3=O)C4=C1C=CC=C4	**6g**	O=C1C2=O=C1C2=C(N(C(C)C)C(C(C(C)=C3)=O)=C2C3=O)C4=C1C=CC=C4=C4

Table 2. Chemical structures of the indeno[1,2-*b*]indoles used for the test set in SMILES format.

Nr.	Chemical structure	Nr.	Chemical structure
**4d**	O=C1C2=C(N(CC(C)C)C(CCC3)=C2C3=O)C4=C1C=CC=C4	**5c**	O=C1C2=C(N(CC)C3=C2C(O)=CC=C3)C4=C1C=CC=C4
**4i**	O=C1C2=C(N(C(C)C)C(CCC3)=C2C3=O)C4=C1C(C(F)(F)F)=CC=C4	**5f**	O=C1C2=C(N(C(C)C)C3=C2C(O)=CC=C3)C4=C1C(C#N)=CC=C4
**4j**	O=C1C2=C(N(C(C)C)C(CCC3)=C2C3=O)C4=C1C(Br)=CC=C4	**6a**	O=C1C2=C(NC(C(C=C3)=O)=C2C3=O)C4=C1C=CC=C4
**4q**	O=C1C2=C(N(C(C)C)C(CCC3)=C2C3=O)C4=C1C=CC(OC(C)=O)=C4	**6c**	O=C1C2=C(N(CC)C(C(C=C3)=O)=C2C3=O)C4=C1C=CC=C4
**5a**	O=C1C2=C(NC3=C2C(O)=CC=C3)C4=C1C=CC=C4	**6e**	O=C1C2=C(N(C(C)C)C(C(C=C3)=O)=C2C3=O)C4=C1C=CC(OC)=C4
